# Host and Symbiont Cell Cycle Coordination Is Mediated by Symbiotic State, Nutrition, and Partner Identity in a Model Cnidarian-Dinoflagellate Symbiosis

**DOI:** 10.1128/mBio.02626-19

**Published:** 2020-03-10

**Authors:** Trevor R. Tivey, John Everett Parkinson, Virginia M. Weis

**Affiliations:** a Department of Integrative Biology, Oregon State University, Corvallis, Oregon, USA; b Department of Integrative Biology, University of South Florida, Tampa, Florida, USA; Pennsylvania State University; University of Hawaii at Manoa

**Keywords:** Aiptasia, *Exaiptasia pallida*, Symbiodiniaceae, cell proliferation, colonization, coral, symbiosis

## Abstract

The cell cycle is a critical component of cellular proliferation, differentiation, and response to stress, yet its role in the regulation of intracellular symbioses is not well understood. To explore host-symbiont cell cycle coordination in a marine symbiosis, we employed a model for coral-dinoflagellate associations: the tropical sea anemone Aiptasia (Exaiptasia pallida) and its native microalgal photosymbionts (Breviolum minutum and Breviolum psygmophilum). Using fluorescent labeling and spatial point-pattern image analyses to characterize cell population distributions in both partners, we developed protocols that are tailored to the three-dimensional cellular landscape of a symbiotic sea anemone tentacle. Introducing cultured symbiont cells to symbiont-free adult hosts increased overall host cell proliferation rates. The acceleration occurred predominantly in the symbiont-containing gastrodermis near clusters of symbionts but was also observed in symbiont-free epidermal tissue layers, indicating that the presence of symbionts contributes to elevated proliferation rates in the entire host during colonization. Symbiont cell cycle progression differed between cultured algae and those residing within hosts; the endosymbiotic state resulted in increased S-phase but decreased G_2_/M-phase symbiont populations. These phenotypes and the deceleration of cell cycle progression varied with symbiont identity and host nutritional status. These results demonstrate that host and symbiont cells have substantial and species-specific effects on the proliferation rates of their mutualistic partners. This is the first empirical evidence to support species-specific regulation of the symbiont cell cycle within a single cnidarian-dinoflagellate association; similar regulatory mechanisms likely govern interpartner coordination in other coral-algal symbioses and shape their ecophysiological responses to a changing climate.

## Abstract (precis)

Biomass regulation is critical to the overall health of cnidarian-dinoflagellate symbioses. Despite the central role of the cell cycle in the growth and proliferation of cnidarian host cells and dinoflagellate symbionts, there are few studies that have examined the potential for host-symbiont coregulation. This study provides evidence for the acceleration of host cell proliferation when in local proximity to clusters of symbionts within cnidarian tentacles. The findings suggest that symbionts augment the cell cycle of not only their enveloping host cells but also neighboring cells in the epidermis and gastrodermis. This provides a possible mechanism for rapid colonization of cnidarian tissues. In addition, the cell cycles of symbionts differed depending on nutritional regime, symbiotic state, and species identity. The responses of cell cycle profiles to these different factors implicate a role for species-specific regulation of symbiont cell cycles within host cnidarian tissues.

## INTRODUCTION

Mutualistic endosymbiotic relationships occupy foundational positions in both terrestrial and marine ecosystems. In these intracellular associations, where symbionts grow and proliferate within host cells, interpartner coordination is essential to maintaining a dynamic, balanced biomass ratio. Symbioses between cnidarians (such as corals, jellies, and sea anemones) and dinoflagellate microalgae (family Symbiodiniaceae; formerly genus *Symbiodinium*) are the trophic and structural foundation of coral reef ecosystems (1). They provide a dramatic example of the importance of regulatory mechanisms for maintaining interpartner homeostasis. Under environmental stress, such associations become dysregulated, leading to dysbiosis and subsequent bleaching, where the cnidarian hosts lose the majority of their photosynthetic symbionts. Without symbiont-derived photosynthate, hosts suffer reduced fitness, increased susceptibility to infectious disease, and greater mortality rates (2–4). Globally, coral reef ecosystems are gravely threatened by bleaching episodes due to rising sea surface temperatures (5). It is therefore crucial to understand how cnidarian-dinoflagellate symbioses are regulated at the cellular level. In a healthy association, there are a variety of mechanisms that help maintain balanced host-symbiont ratios, including: expulsion of symbionts via exocytosis, host cell apoptosis, host cell necrosis, and host cell detachment (6); symbiont degradation via host autophagic degradation and symbiont apoptosis or necrosis (7); and host and symbiont cell cycle regulation (8). Although coordination between host and symbiont cell cycles is a fundamental aspect of terrestrial and protozoan symbioses (9–12), the role of the cell cycle in the regulation of marine cnidarian-dinoflagellate symbioses remains largely unexplored (13).

**FIG 1:**
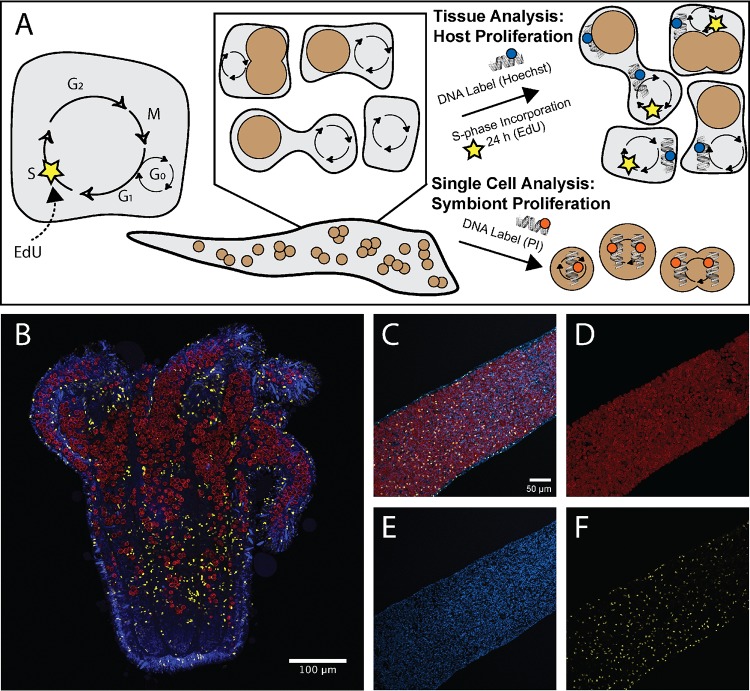
Fluorescent labeling techniques used to correlate host nucleus proliferation and symbionts in Aiptasia-Symbiodiniaceae symbiosis. (A) Fluorescent labels were used to identify proliferating populations of host cells and cell cycle populations of symbiont cells. The presence of EdU incorporation over 24 h marked host cell proliferation, whereas all host nuclei were labeled with Hoechst stain. Symbionts were isolated from hosts or cultures and were labeled with propidium iodide to identify cell cycle populations based on DNA content. (B) Tiled section of a symbiotic Aiptasia sea anemone. Symbiont location was captured using chlorophyll autofluorescence shown in red. Cnidarian host nuclei were labeled with Hoechst stain (blue), and proliferating host nuclei were labeled with EdU-AF555 (yellow) to capture cell populations that have incorporated new DNA during S phase. (C to F) Host cell proliferation analyses were performed using fluorescently labeled Aiptasia tentacles (C) to allow for quantification and location mapping of symbionts (D), cnidarian host nuclei (E), and proliferating host nuclei (F).

In eukaryotes, cells are either quiescent or undergoing a pattern of cell growth, DNA replication, and cell division known as the cell cycle. Cells form two daughter cells through the steps of G_1_ phase (cell growth and replication preparation), S phase (DNA replication), G_2_ phase (cell growth and DNA damage checkpoint), and M phase (mitosis) (Fig. 1A). Though many factors influence cell cycle progression and subsequent proliferation rate, certain conditions, such as nutritional state, commonly arrest and augment cell growth in G_1_ phase prior to DNA replication (14, 15). In photoautotrophic cells, the light/dark cycle generally synchronizes DNA replication and cell division (16, 17). The symbiotic state adds complexity to the regulation of host and symbiont cell cycles. Partners may modulate the nutrients, growth factors, metabolic state, and/or toxins they provide one another, which will influence cell cycle progression (18–20). In the cnidarian-dinoflagellate symbiosis, direct cell cycle modulators have not been discovered, and the mechanisms that govern cell cycle regulation remain unclear.

Several studies have characterized the cell cycles of cnidarian cell populations (21, 22) and examined proliferation during development and regeneration (23, 24). For example, gastrodermal cells are slower to proliferate than epidermal cells, which must rapidly regenerate new mucocytes and venomous cnidocytes for defense and prey capture. In contrast to the multicellular cnidarians, dinoflagellates are haploid and unicellular. Photosynthetic species divide in response to the diel light/dark cycle. Dinoflagellates exhibit additional structural complexity in their DNA: chromosomes remain condensed throughout the cell cycle and lack nucleosomes and functional histones, and the cells use extranuclear spindles to accommodate the unusual persistence of the nuclear envelope during mitosis (25–27). The cell cycles of cnidarians have been most closely examined in nonsymbiotic taxa such as the hydrozoan *Hydra* spp. (21, 28, 29), while those of dinoflagellates have been studied in the free-living, heterotrophic Crypthecodinium cohnii (30–34). This focus on nonsymbiotic organisms has left a gap in our understanding of how interactions between symbiotic species may influence cell cycle dynamics in each partner. Characterizing these dynamics is critical because the cnidarian-dinoflagellate mutualism occupies a foundational role in building coral reefs, and changes at the cellular level have broad implications for how these ecosystems may persist under ongoing climate change.

The Aiptasia-Symbiodiniaceae mutualism is a model system for the study of coral-dinoflagellate cell biology. The sea anemone Aiptasia (Exaiptasia pallida) falls within class Anthozoa alongside corals, and it has been used extensively to study cnidarian cellular and molecular processes involved in the onset, maintenance, and breakdown of symbiosis (35–37). Similarly to corals, Aiptasia forms nutritional endosymbioses with several species of Symbiodiniaceae whereby the unicellular algae reside inside host gastrodermal cells within vesicles called symbiosomes. Unlike many corals, Aiptasia can be maintained symbiont free (aposymbiotic), enabling comparisons between hosts with and without symbionts. The primary symbiont of Aiptasia across the globe is Breviolum minutum (ITS2 type B1), though it can be found associating with Breviolum psygmophilum (ITS2 type B2) and certain other Symbiodiniaceae in the western Atlantic (38, 39). Smith and Muscatine (40) examined the nutritional regulation of G_1_ phase in *B. minutum in hospite* (within the host Aiptasia polyp) and found that transfer of nutrients such as nitrogen and phosphorus from host to symbiont cells constrains symbiont cell cycle progression. They also found that the host cell environment removes the light/dark cell division patterns found in cultured Symbiodiniaceae cells. A variety of studies have characterized Symbiodiniaceae cultures and isolates under different growth conditions, along with their proliferation and growth (41–45). In *Breviolum* spp., increased growth rates have been measured in cultures compared to freshly isolated symbionts (40), and growth variation among species has been observed under shared culture conditions (46). The division and proliferation of Aiptasia cells have also been studied previously (47–49); however, the relationship between the two partners requires further investigation.

A key challenge in studying the cell biology of the Aiptasia-Symbiodiniaceae mutualism and other anthozoan mutualisms is the small host-to-symbiont cell size ratio. The cytoplasm of a typical symbiont-containing host gastrodermal cell is almost completely filled by 1 to 5 Symbiodiniaceae, which are ∼10 μm in diameter (see reference 13), in contrast to symbiotic hydroid cells, which are much larger and accommodate ≥25 symbionts at a time. This makes determining boundaries between Aiptasia cells difficult, and it is nearly impossible to visually match a host nucleus with the symbionts contained within that cell at tissue-level scales (e.g., across a whole Aiptasia tentacle). In addition to this challenge, Symbiodiniaceae cells *in hospite* possess a thick internal cell wall and a peripheral chloroplast with a wide photosynthetic absorption range that results in high autofluorescence during microscopy. Together, these algal characteristics make it difficult for the penetration and visualization of intracellular fluorescent probes even after fixation (50–52).

As a result of these challenges, there have been only a few studies performed *in situ* within symbiotic anthozoan tissues that have examined the proliferation of either the cnidarian hosts (47–49, 53), their symbionts (54, 55), or both partners (23). Many more symbiont proliferation studies have been performed at an organismal level after substantial manipulations such as macerations or homogenization (40, 44, 45, 56–61). Of these studies, just two have examined host and symbiont cell cycles simultaneously and revealed evidence of coordination between partners. In the temperate sea anemone Anthopleura elegantissima, hosts with elevated G_2_/M-phase cell populations contain symbiont populations with corresponding elevated G_2_/M-phase cell populations (57). During primary polyp development in the coral Stylophora pistillata, the coordinated proliferation of host gastrodermal cells and symbiont cells results in a dramatic increase in Symbiodiniaceae cell density and a switch to apoptotic postmitotic control in the host (23).

Here, we describe cell cycle progression in the cnidarian host Aiptasia and its dinoflagellate symbionts under different symbiotic states and nutritional regimes. We used a variety of imaging techniques, including novel analysis of fluorescence images designed to circumvent some of the challenges inherent in Aiptasia-Symbiodiniaceae microscopy (Fig. 1A). We compared anemones under two symbiosis conditions: during recolonization of hosts by symbionts and in the stable aposymbiotic state. Using fluorescent labeling and confocal microscopy, we first investigated whether the presence of symbionts had an effect on the cell cycle and proliferation of Aiptasia host cells (Fig. 1B). We then further explored the interaction effect between symbiotic state and host nutritional state on the cell cycles of two symbiotic *Breviolum* species that are found naturally in Aiptasia. Our results suggest that Aiptasia alters its cell cycle progression based on host-to-symbiont biomass ratios and that the symbionts regulate their cell cycles in a nutrition-dependent and species-specific manner. These results provide a broader understanding of how cell populations of hosts and symbionts respond to each other and to the environment through their cell cycle dynamics.

## RESULTS

### Host cells proliferate faster when in close proximity to colonizing symbionts.

**FIG 2:**
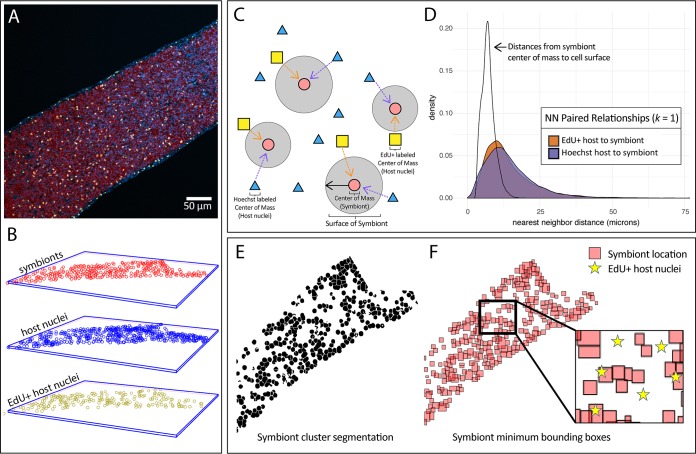
Spatial analysis of proliferating host nuclei (EdU^+^) and symbionts during colonization. (A) Confocal z-stack images were taken of host tentacles during colonization by symbionts. Host cells were differentiated with Hoechst stain (blue) to label nuclei and with EdU-AF555 (yellow) to label proliferating nuclei, and symbiont cells were differentiated by chlorophyll autofluorescence (red). (B) The centers of mass of host nuclei (blue), proliferating host nuclei (yellow), and symbionts (red) were located for each tentacle in three-dimensional space. (C) For each tentacle, the distances from each center of mass to the nearest neighbor (NN) of another group were determined. (D) NN distances were combined across tentacles, and density plots of these distances were made for the first NN. The distribution of distances from a symbiont center of mass to its cell surface is provided for context. (E) In a separate analysis, the segmented symbiont objects from each tentacle were used to determine the presence/absence of symbionts in specific locations. A representative z-slice from a z-stack image shows symbiont segmentation in a tentacle. (F) 3D symbiont objects created from symbiont segmentation were used to construct 3D minimum bounding boxes representing the height, width, and length of each symbiont. A representative 2D z-slice of these 3D boxes in a tentacle is displayed for clarity. The number of EdU-positive nuclei (yellow stars) was quantified inside and outside corresponding symbiont-containing locations and compared to a null hypothesis where one would expect proliferating nuclei to be found in equal proportion inside and outside these symbiont-containing volumes.

**FIG 3:**
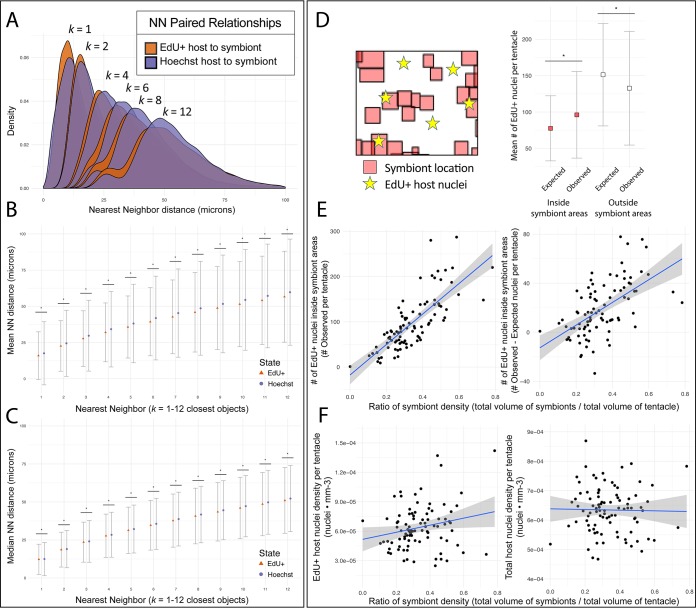
NN distributions and overlap of host nuclei and symbiont-containing areas during colonization. (A) After the distances from each center of mass to the nearest neighbor (NN) of another group were determined in three dimensions, NN distances were combined across tentacles. Density plots of these distances are shown for the first NN *k* = 1 up to the 12th closest NN *k* = 12. (B and C) Statistical comparisons were made between the means (B) and medians (C) of these distributions. Error bars indicate standard deviation (B) and median absolute deviation (C), and asterisks indicate significance (Mann-Whitney *U* test, *P* < 0.0004). (D) Using a separate method, segmented symbiont objects were used to determine the presence or absence of symbionts in specific locations of the tentacle. The number of EdU-positive nuclei was quantified inside (red) and outside (white) corresponding symbiont-containing locations and compared to a null hypothesis where one would expect proliferating nuclei to be found in equal proportion inside and outside these symbiont-containing volumes. Asterisks indicate significance (paired *t* test; *P* < 1 × 10^−5^). (E) To further examine correlation in local tissue proliferation, the observed numbers of proliferating EdU^+^ nuclei within symbiont-containing locations were plotted against a ratio of symbiont density for each imaged tentacle (*n* = 95, *R* = 0.60). The differences between the observed and expected EdU^+^ nuclei in symbiont-containing areas were also plotted against a ratio of symbiont density (*R* = 0.32). (F) To examine the nonlocalized effect of symbiont presence on tentacle proliferation, the total tentacle densities (i.e., both inside and outside symbiont regions) of EdU^+^ host nuclei (*R* = 0.04) and nonproliferative host nuclei (*R* = 0.00) were plotted against a ratio of symbiont density.

To test for localized host cell cycle patterns and their relationship to the presence of symbionts, we sampled Aiptasia tentacles during colonization and visualized all host nuclei with Hoechst stain, proliferating host nuclei with 5-ethynyl-2′-deoxyuridine (EdU), and symbiont cells with chlorophyll autofluorescence (Fig. 2). The total number of symbiont cells in these partially colonized tentacles ranged from 100 to 1,700 cells per tentacle, with an average cell density of 5.0 × 10^5^ cells/mm^3^. To compare our symbiont densities with previous studies that examined host and/or symbiont proliferation using area, we estimated symbionts per area and found densities to be between 3.2 × 10^3^ cells/mm^2^ and 1.2 × 10^4^ cells/mm^2^. These estimates closely match symbiont densities of Aiptasia during bleaching recovery and are 10-fold higher than the densities of successful 2-day symbiont inoculations in Aiptasia (47, 48, 62). Using a nearest-neighbor (NN) analysis, we found the first-order (*k* = 1) NN distances from both the proliferating-host-cell group and the all-host-cell group to symbiont cells (Fig. 2C). To provide context for the proximity of this distance, we measured distances from each symbiont center of mass to its cell surface and found the average and median distances to be 7 μm (Fig. 2D). The majority of host nuclei proliferation (54.2%) occurred within 13 μm of a symbiont’s center of mass, i.e., within 6 μm of the surface of a symbiont on average. At *k* = 1, proliferating host nuclei were distributed closer to symbionts than were all nuclei when means (16.0 μm versus 17.6 μm; *t* test, *P* < 2.2 × 10^−16^) (Fig. 3B) and medians (12.3 μm versus 12.5 μm; Mann-Whitney *U* test, *P = *0.0004) (Fig. 3C) were compared. At every subsequent neighbor pair tested up to the 12th pair (*k* = 12) (Fig. 3A), the mean NN distances of the proliferating-host-cell group were nearer to the symbiont centers than were the distances of the all-host-cell group (22.7 to 56.7 μm versus 24.5 to 59.8 μm; *t* test, all *P* < 2.2 × 10^−16^) (Fig. 3B), as were the median distances (18.6 to 51.0 μm versus 19.0 to 52.2 μm; Mann-Whitney *U* test, all *P* < 6.02 × 10^−11^) (Fig. 3C).

Proliferating host nuclei appeared predominantly within symbiotic rather than aposymbiotic regions of tentacles (Fig. 3D). Compared to the null hypothesis of neutral dispersal, the enrichment of proliferating host cells near symbiont clusters was significant (χ-square test, *P* < 1 × 10^−5^; paired *t* test, *P* < 1 × 10^−5^) (Fig. 3D). Symbiont density had a strong positive correlation with the number of proliferating host cells found within symbiotic regions (*R* = 0.5986, *F* = 135.2, *P* < 2.2 × 10^−16^) (Fig. 3E). Increased symbiont density correlated with a larger positive difference between the observed and the expected number of host nuclei under the null hypothesis of neutral dispersal (*R* = 0.3184, *F* = 43.04, *P* < 3.44 × 10^−9^) (Fig. 3E). Symbiont density weakly correlated with the total density of proliferative host cells (*R* = 0.037, *F* = 4.49, *P* = 0.037) (Fig. 3F) and did not correlate with total host cell density (*R* = 0.000, *F* = 0.03, *P* = 0.854) (Fig. 3F) or the total number of proliferating host cells found in both symbiotic and aposymbiotic regions (*R* = −0.0105, *F* = 0.07, *P* = 0.779).

### Host cells proliferate faster in symbiotic anemones than in aposymbiotic anemones.

**FIG 4:**
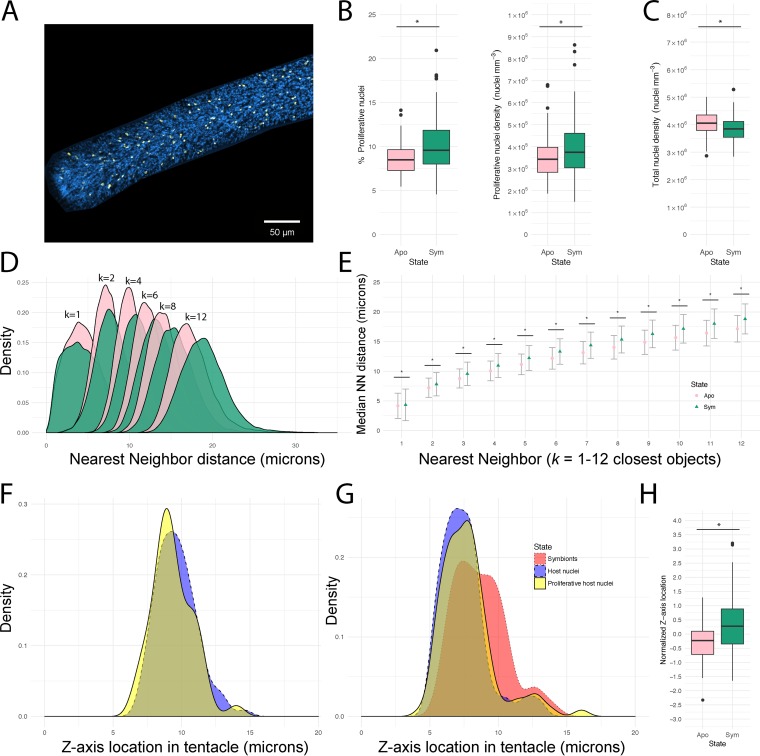
Comparative analysis of host EdU-positive nuclei in aposymbiotic and symbiotic sea anemones. (A) Confocal z-stack images were taken of aposymbiotic tentacles in order to compare to proliferation during colonization by symbionts. Host cells were differentiated with Hoechst stain (blue) to label nuclei, with EdU-AF555 (yellow) to label proliferating nuclei. (B and C) The percentage and density of host proliferative nuclei (B) and total host nuclei (C) were compared across symbiotic states. Asterisks indicate significant differences (two-sample *t* test, *P* < 0.05). (D) For each tentacle, the distances from each center of mass of a proliferative cell were measured to the nearest-neighbor (NN) nonproliferative cell. A density plot compares the distributions of NN distances in aposymbiotic (pink) and symbiotic (green) Aiptasia tentacles. (E) Median NN distances of aposymbiotic and symbiotic NN distributions. Error bars represent median absolute deviation; asterisks indicate significant differences (Mann-Whitney *U* test, *P* < 2.2 × 10^−16^). (F and G) Distributions of cell centers of mass were plotted to visualize *z*-axis location in aposymbiotic (F) and symbiotic (G) tentacles. The epidermal tissue layer is represented by the left peak of Hoechst stain-labeled host cells (blue) whereas the gastrodermal tissue layer is right-shifted as evident from the location of symbionts (red) in symbiotic tentacles. (H) To compare proliferative nucleus *z*-axis locations, distributions were normalized for each tentacle by subtracting the distribution medians of host nuclei from the medians of proliferative nuclei. A positive normalized *z*-axis location therefore represents a rightward shift toward the gastrodermis for proliferative nuclei. Asterisks indicate significant differences (two-sample *t* test, *P* < 1 × 10^−6^).

To further characterize the influence of symbiosis on host cell proliferation rates, we compared recolonized and aposymbiotic anemones (Fig. 4A). Relative to symbiotic tentacles, aposymbiotic tentacles featured fewer proliferating host nuclei as measured by both proportion (8.6% proliferating versus 10.2% proliferating; two-sample *t* test, *P* = 8.7 × 10^−5^) (Fig. 4B) and density (3.5 × 10^5^ cells/mm^3^ versus 3.9 × 10^5^ cells/mm^3^; two-sample *t* test, *P* = 0.02) (Fig. 4B). This decrease in proliferating cells in aposymbiotic animals was not a result of decreased host cell density, as aposymbiotic anemones had higher cell density (4.1 × 10^6^ cells/mm^3^ versus 3.8 × 10^6^ cells/mm^3^; *P* = 0.002) (Fig. 4C) and smaller estimated cell diameters (∼7.80 μm versus ∼7.95 μm; two-sample *t* test, *P* = 0.002). Proliferating host nuclei were located closer to all host nuclei in aposymbiotic tentacles (NN *k* = 1 to 12, 4.2 to 17.4 μm versus 4.6 to 19.0 μm; *P* < 2.2 × 10^−16^) (Fig. 4D). Aposymbiotic tentacles also featured smaller median NN distances between proliferating and total host cells (NN *k* = 1 to 12, 4.2 to 17.1 μm versus 4.3 to 18.8 μm; Mann-Whitney *U* test, *P* < 2.2 × 10^−16^) (Fig. 4E). These data reflect the higher cell density in aposymbiotic anemones and suggest a change of location in proliferation toward the nucleus-dense tissue of the epidermis. To test for this potential spatial shift in tissue proliferation, we compared the positional location of proliferating nuclei from the surface of the tentacle (e.g., *z* = 5 μm) (Fig. 4F and G) to the deeper gastrodermis (e.g., *z* = 10 μm) (Fig. 4F and G). As expected, aposymbiotic anemones collectively had a proliferative peak within shallow *z*-axis locations correlating with the epidermis (Fig. 4F). In symbiotic anemones, however, the proliferating host cells were shifted toward the deeper, symbiont-containing gastrodermal layer (two-sample *t* test, *P* < 7 × 10^−5^) (Fig. 4G and H). When the location of symbionts was used to estimate the epidermal-gastrodermal border for each image, gastrodermal tissues of symbiotic anemones ended up containing 41.7% of proliferating cells and 56.6% of total cells measured.

### Symbiodiniaceae cell cycles respond in a species-specific manner to symbiotic and nutritional states.

**FIG 5:**
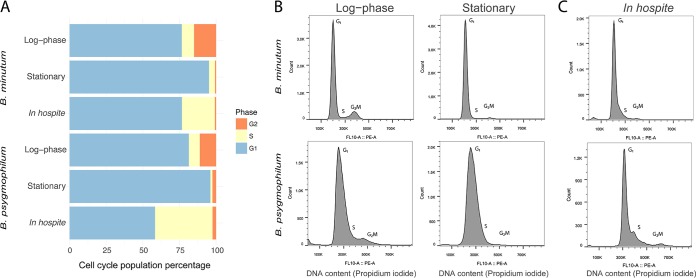
Cell cycles of *B. minutum* and *B. psygmophilum* in culture and *in hospite*. (A) The cell cycles of two *Breviolum* species in culture and *in hospite* were analyzed under different nutritional states using propidium iodide staining of DNA and flow cytometry. Stacked horizontal bar graphs represent cell cycle percentages (blue = G_1_, yellow = S, orange = G_2_/M) of *Breviolum* cultures and isolates. The log-phase and stationary cell cultures of *B. minutum* and *B. psygmophilum* were compared to their respective cell cycles *in hospite*. (B and C) Representative cell cycle profiles are shown for log-phase and stationary *Breviolum* cultures (B) and *Breviolum* populations isolated from host Aiptasia strains H2 (*B. minutum*) and JK (*B. psygmophilum*) (C). Cellular DNA content was measured using propidium iodide, and fluorescence was captured in the FL10: PE-A emission channel after doublet discrimination. Units represent relative propidium iodide fluorescence. *B. psygmophilum* had right-shifted G_1_ peaks representing increased DNA content compared to *B. minutum*. G_2_ peaks for both species were found at double the fluorescence (i.e., 2× DNA content) of the G_1_ peaks.

Using flow cytometry, we examined cell cycle dynamics in two species of Symbiodiniaceae both *ex hospite* and *in hospite* under different nutritional regimes. Algal cultures and isolates were fixed and labeled with propidium iodide to enable cell cycle profiling based on DNA content. In the cultures of both species, proliferating cells (those cell populations in S phase and G_2_/M phase) were elevated in treatments with freshly replaced, nutrient-replete f/2 medium compared to older, nutrient-limited medium, which instead contained elevated G_1_-phase populations (two-sample *t* tests, *P* < 0.05 in all cases) (Fig. 5A; see also Table S2 in the supplemental material). Representative cell cycle profiles are provided to show these differences (Fig. 5B). These profiles also revealed that the average genomic content of the G_1_ peak is larger in *B. psygmophilum* than in *B. minutum*; this difference allowed for rapid species identification, which we confirmed by genotyping. In host tissues, both species featured distinct cell cycle populations compared to cultures. S-phase populations were elevated in symbiont samples isolated from hosts compared to both culture conditions (two-sample *t* tests, *P* < 0.05) (Fig. 5A). In addition, *B. psygmophilum* had a distinct S-phase peak that was absent from *B. minutum* (Fig. 5C). In both species, symbionts isolated from hosts had G_2_-phase populations that were not significantly different from stationary culture conditions and decreased G_2_/M-phase populations compared to their respective log-phase algal cultures (two-sample *t* tests, all *P* < 0.05) (Fig. 5A to C; Table S2).

**FIG 6:**
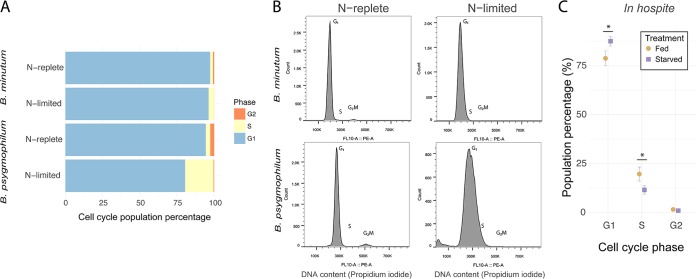
Cell cycles of *B. minutum* and *B. psygmophilum* in culture and *in hospite*. (A) Stacked horizontal bar graphs represent cell cycle percentages (blue = G_1_, yellow = S, orange = G_2_/M) of *Breviolum* cultures grown under nitrogen-replete and nitrogen-limited conditions. (B) Representative cell cycle profiles of nitrogen-replete and nitrogen-limited cultures are shown for both *Breviolum* cultures. (C) To examine the effect of nutrition on *Breviolum* populations *in hospite*, cell cycle percentages of *B. minutum* symbionts were measured from fed (brown circles) and starved (purple squares) Aiptasia hosts. The population percentages of symbionts are shown for each cell cycle phase (G_1_, S, and G_2_). Asterisks represent significant differences (two-sample *t* test, *P* < 0.05).

We then compared the cell cycle responses of cultured *B. minutum* and *B. psygmophilum* to nitrogen limitation by using f/2 medium with and without added nitrate (NaNO_3_). For both species, nitrogen limitation led to elevated S-phase cell populations and depressed G_2_/M-phase cell populations compared to their respective nitrogen-replete treatments (two-sample *t* tests, *P* < 0.05 in all cases) (Fig. 6A). However, the effect was much stronger in *B. psygmophilum* than in *B. minutum* cultures (e.g., 7-fold versus 2-fold increase in S-phase cell populations). In addition, both species under nitrogen limitation had a G_1_-phase peak distribution coefficient of variation (CV) with a larger width than the nitrogen-replete treatment (two-sample *t* test, all *P* < 0.001) (Fig. 6B). Again, the effect was larger for *B. psygmophilum* (e.g., 2.5-fold versus 1.5-fold increase in CV width). In addition, nitrogen-limited populations in both species exhibited increased forward scatter, a common indicator of cell size (Fig. S2).

To test how supplemental nutrition affected *B. minutum in hospite*, we examined the cell cycles of symbionts isolated from Aiptasia that were either starved for 2 weeks or fed *Artemia* nauplii. Unlike nitrogen-limited cultures, G_1_-phase populations increased and S-phase populations decreased in starved treatments compared to fed treatments (two-sample *t* tests, *P* < 0.05 in all cases) (Fig. 6C). Compared to the major decreases of G_2_/M phase populations in nitrogen-limited cultures, G_2_/M-phase populations *in hospite* only slightly decreased from 1.5% to 0.9% (two-sample *t* test, *P* = 0.18) (Fig. 6C).

## DISCUSSION

### Spatial coordination of anemone and symbiont cell proliferation occurs during recolonization.

We report the first empirical evidence in support of locally coordinated cell cycle regulation between cnidarian gastrodermal cells and dinoflagellate symbionts. In our spatial examination of recolonizing anemone tissues, the nearest-neighbor (NN) distributions (Fig. 3A to C) and *z*-axis coordinates (Fig. 4F to H) locate a majority (54.2%) of proliferating host nuclei within 13 μm of a symbiont center of mass, and an unusually large proportion (41.7%) of proliferating host nuclei within the gastrodermal layer. Upregulation of host cell proliferation during recolonization is therefore inferred to be primarily occurring within the gastrodermal layer. Indeed, the proportion of gastrodermal host cells undergoing proliferation is much higher than levels previously found in fully symbiotic Aiptasia and more closely resembles short-term bleaching recovery time points (47, 49, 63). In addition to this generalized tissue-level upregulation, a majority of these proliferating gastrodermal host cells have a strong chance of containing symbionts: 22% of proliferating host nuclei are located within 8 μm of a symbiont center of mass, which is close considering the average distance of 7 μm from symbiont surface to its center of mass. Similar localized host proliferation has been found in the giant clam Hippopus hippopus symbiosis during periods of symbiont proliferation (64). This colocalized gastrodermal proliferation suggests a direct mechanism of host-symbiont cell communication, possibly through a combination of cell signaling and metabolic exchange. Such an effect would be consistent with previously described gene expression differences between symbiotic and aposymbiotic anemones, including genes involved in the cell cycle and DNA regulation (65, 66).

In other systems, there is evidence that symbiotic organisms can directly manipulate the cell cycles of their partners. For example, in the relationship between animal hosts and parasitic apicomplexans (a sister taxon to dinoflagellates), the intracellular parasite Toxoplasma gondii manipulates its host into cell cycle dysregulation and arrest in the G_2_ stage (67, 68). In another apicomplexan, *Theileria* is able to induce host division via NF-κB pathway activation (69). Plasmodium falciparum takes advantage of melatonin to induce synchronicity with its development and growth inside its host (70, 71). In bacterial symbioses, plants and weevils inhibit division of endosymbiotic bacteria by using specific peptides (10, 72), whereas the bacteria *Algoriphagus* sp. and Aliivibrio fischeri use lipids and enzymes to stimulate cell division and mating, respectively, in the host choanoflagellate Salpingoeca rosetta (73, 74). Future work should attempt to characterize similar pathways in the Aiptasia-Symbiodiniaceae system by monitoring the continuous interactions between host and symbiont cells at high resolution within the gastrodermal layer during coordinated proliferation.

Whereas localized gastrodermal proliferation suggests direct host-symbiont regulation, host epidermal proliferation is likely a result of communication among host cells. Based on the z-stack position of total host nuclei and proliferating host nuclei, a large proportion (58.3%) of proliferating host cells were localized in the epidermal layer of symbiotic hosts. It is likely that this epidermal proliferation is needed to accommodate expanding gastrodermal tissue and growth in the overall size of symbiotic tentacles. Gastrodermal host cells with nutrients provided from symbionts could signal epidermal host cells via growth factors and signaling pathways commonly used to promote localized growth and proliferation. Cnidarians have been found to upregulate Wnt, insulin-like growth factor (IGF), and transforming growth factor β (TGF-β) signaling during colonization by symbionts (75–77).

### Recolonization of anemones by algae is associated with host gastrodermis proliferation.

The positive effect of symbiont presence on host cell proliferation was evident not only between symbiotic and aposymbiotic tentacle regions within a recolonizing anemone (Fig. 3) but also between symbiotic and aposymbiotic organisms (Fig. 4). The positive correlation between symbiont density and the density of proliferative host cells across tentacles suggests that the host and its symbiont promote each other’s growth during this time of colonization. This mechanism is most probably through nutrient exchange, where both partners are able to benefit from alga-produced photosynthate and host waste ammonium. The overall increase in gastrodermal proliferation in symbiotic Aiptasia further supports a targeted expansion of the gastrodermis caused by the presence of symbiont populations. Further experiments could examine the host and symbiont cell proliferation under stable conditions, when host regulation of the symbiont cell cycle would be expected to slow symbiont proliferation as a result of nitrogen limitation (78–81). In contrast to the fast-growing symbiont population densities of partially colonized hosts, these symbiont population densities within fully colonized cnidarian hosts have both elevated C/N ratios and elevated transcripts of genes involved in nitrogen assimilation, which would suggest a population control mechanism after colonization (81).

In our tissue comparisons between aposymbiotic and partially recolonized anemones, we also observed a shift of proliferative host cells toward the epidermis in the aposymbiotic state based on shorter NN distances and shallower z-stack depth (Fig. 4F). This result is consistent with previous studies, which have shown that the epidermal tissue layer in cnidarians proliferates at a higher rate than the gastrodermal layer (24, 47, 49). In Aiptasia, loss of symbionts induced by thermal stress or photosynthesis inhibitors causes elevated epidermal proliferation, possibly to facilitate a switch to a primarily heterotrophic feeding strategy (47, 48).

### Nutritional state mediates symbiont cell cycle dynamics.

Nutrition had a strong effect on the cell cycles of the two *Breviolum* species. In culture, the increased G_1_-phase arrest and cell size of algal cell populations in both nutrient-exhausted media (stationary phase, Fig. 5A) and nitrogen-limited media (Fig. 6A; see also Fig. S2 in the supplemental material) confirm previous work examining cultured Symbiodiniaceae growth rate and cell size in stationary- and log-phase growth (82, 83). Nitrogen limitation causes G_1_-phase arrest and wide CVs in a range of microalgae, including other dinoflagellates (20, 84, 85). Though the similar trends in the two *Breviolum* cultures indicate a common cell cycle response, the difference in magnitude suggests that the cell cycle of *B. psygmophilum* is more sensitive to nutrient dynamics than that of *B. minutum*, providing another example of important functional diversity among Symbiodiniaceae species (86). *B. psygmophilum*’s sensitivity to nitrogen may reflect its evolution in temperate regions, which are nutrient rich (eutrophic), and help explain why it is replaced by *B. minutum* as the primary Aiptasia symbiont in the tropics, which are nutrient poor (oligotrophic). Temperate symbionts can afford to be nutrient sensitive, while tropical symbionts cannot.

*In hospite*, increased G_1_-phase populations of *B. minutum* in starved compared to fed hosts matched the G_1_-phase arrest phenotype found in nutrient-limited cultures (Fig. 6C). These results are similar to previous studies of Aiptasia, where the proportion of mitotic populations of Symbiodiniaceae isolates was greater and the proportion of G_1_-phase-arrested populations lesser when animals were provided with nitrogen or phosphorus (40, 41). Additional aspects of symbiont physiology change with the nutritional state of hosts: starved Aiptasia cnidarians drive Symbiodiniaceae phenotypes that include increased cell sizes and starch and lipid stores (87, 88). Symbiodiniaceae cell cycle arrest phenotypes have been observed as a result of treatment with cerulenin, an inhibitor of free fatty acid synthesis (43). Nutrient balance therefore appears to strongly affect the regulation of symbiont proliferation in cnidarian hosts and is of consequence when considering the effect of nutrient enrichment from agricultural runoff on the vulnerable cnidarian-dinoflagellate symbioses that compose coral reefs (79, 89).

### Decreased G_2_/M phase of *Breviolum* species is associated with symbiotic state.

The cell cycle progression of *Breviolum* in culture was similar to other Symbiodiniaceae (42, 61), whereas when *in hospite*, both species had increased S-phase and decreased G_2_/M-phase populations compared to log-phase cultures (Fig. 5A and C). The observed increases in S-phase populations *in hospite* over *ex hospite* may truly reflect higher levels of DNA replication during the period sampled, i.e., during the light period of the light/dark cycle. Whereas the replicating and mitotic populations of Symbiodiniaceae cultures generally increase during the dark period (42, 61), the increased replicating populations of symbionts *in hospite* may be a result of losing their tight linkage with the dark period. Increased S-phase populations may alternatively be a product of wider CVs in the G_1_ peaks. Though it is possible that these wider CVs observed *in hospite* arose as an artifact from sample preparation of homogenized isolates compared to single cell cultures, the increased G_1_-phase variation could also represent biological variation in DNA content similar to the wider CVs found in nitrogen-depleted cultures (90). Host starvation typically results in nitrogen stress to the symbiont, which reduces metabolism and cell division (41, 80). These decreased symbiont G_2_/M populations *in hospite* lend further support to the idea that nutrient limitation by the host is a strategy for symbiont regulation in nutrient-starved cnidarian hosts (79, 81, 91, 92). Symbiont population growth is thought to rely on the assimilation of nitrogen in the form of waste ammonium from the host environment in order for successful colonization. When there is a stable population of symbionts, however, cnidarian hosts may be able to use symbiont photosynthate to reduce their own ammonium production through nitrogen conservation (93–95). In fed hosts, however, the observed S-phase increase and G_2_/M-phase decrease are more consistent with other mechanisms of premitotic control such as increased expulsion of late S-phase and G_2_/M-phase cell populations (56).

### Symbiodiniaceae cell cycles are regulated in a species-specific manner *in hospite*.

Though both *B. minutum* and *B. psygmophilum* were arrested in G_1_ phase under nutrient-limiting conditions *ex hospite*, the two species differed in their cell cycle states *in hospite*. Compared to their respective cell cycles in culture, *B. psygmophilum* had a more substantial increase in S-phase populations (and therefore greater proliferation) than the small increase observed in *B. minutum* when associating with Aiptasia (Fig. 5C). This interactive effect (symbiont genotype by host cellular environment) on Symbiodiniaceae cell cycle dynamics likely reflects both the species-specific sensitivity of symbionts to nitrogen and the acclimation of temperate holobionts to eutrophic conditions and tropical holobionts to oligotrophic conditions. Although *B. psygmophilum* can be found in the tropics, it typically thrives as a symbiont of cnidarians in temperate regions, in part due to its relative cold tolerance (38, 96). Temperate holobionts are exposed to more nutrients than their tropical counterparts and are therefore less limited by nitrogen (97, 98). Symbiodiniaceae are often nutritionally limited within hosts, but they tend to have higher growth rates in symbiosis with hosts that are exposed to more environmental nutrients (44, 91). Thus, Aiptasia cnidarians from temperate waters may be less “greedy” with their nutrients than those from the tropics, where nutrient exchange is likely optimized for constant limitation. This could explain why even under shared laboratory conditions, we observed more symbiont proliferation in *B. psygmophilum* within temperate anemones than in *B. minutum* within tropical anemones. Further experimentation will be required to directly test this hypothesis.

### Conclusions.

Our data reveal cell cycle modulation by both cnidarian hosts and algal symbionts and support hypothesized interpartner regulation of host and symbiont cell cycles during the establishment and maintenance of cnidarian-dinoflagellate symbiosis. Host and symbiont cells showed coordinated localized proliferation during recolonization of hosts by symbionts. The host cell proliferation rate was higher in recently colonized symbiotic anemones than in aposymbiotic anemones and originated from the expansion of gastrodermal cell proliferation with a growing symbiont population. The local proliferation patterns found within gastrodermal tissue suggest a mechanism for host-enabled rapid symbiont dispersal throughout a symbiotic cnidarian. In addition, we found differences in the cell cycle populations of two different species of *Breviolum in hospite*, indicating species-specific cell cycle regulation among Symbiodiniaceae. A basic understanding of host and symbiont cell cycle dynamics is critical for establishing a more complete picture of the cellular mechanisms that regulate cnidarian-dinoflagellate symbioses under normal circumstances as well as in a changing climate.

## MATERIALS AND METHODS

### Maintenance of anemones and algae.

Symbiotic Aiptasia polyps and Symbiodiniaceae cultures were maintained on a 12-h:12-h light/dark cycle (approximately 40 μmol photons m^−2^ s^−1^) at room temperature. Aposymbiotic anemones were generated by menthol bleaching (99). Menthol was added from a stock 20-g/liter menthol in ethanol to a final concentration of 0.58 mM in filtered artificial seawater (FSW). Anemones were treated for repeated 3-day time periods until no autofluorescence from algal chlorophyll was detected, and they remained in the dark for at least 1 month prior to experiments. All experimental anemones had an oral disc diameter of approximately 0.5 cm, were fed brine shrimp nauplii three times weekly, and were starved 1 week prior to the beginning of experiments unless otherwise indicated. Primary experiments were performed with the clonal host strain H2 symbiotic with *B. minutum* (see Table S1 in the supplemental material). Additional experiments measuring cell cycle phenotypes of symbionts *in hospite* were performed with host strains VWB9 and VWA12 containing *B. minutum* and JK containing *B. psygmophilum*. Cell cultures of *B. minutum* (CCMP830, FLAp2, and Mf1.05b) and *B. psygmophilum* (HIAp) were grown in f/2 medium (Table S1). To confirm symbiont species identity, Symbiodiniaceae were sampled from representative host tissues and cultures and then genotyped following DNA extraction using the ITS2 ribosomal DNA (rDNA) marker as described by LaJeunesse (100).

10.1128/mBio.02626-19.4 TABLE S1 Aiptasia and Symbiodiniaceae strain information. Collection source locations for each clonal strain used are provided as well as the internal transcribed spacer 2 (ITS2) type. Download Table S1, DOCX file, 0.01 MB. Copyright © 2020 Tivey et al. 2020 Tivey et al. This content is distributed under the terms of the Creative Commons Attribution 4.0 International license.

10.1128/mBio.02626-19.5 TABLE S2 Cell cycle distribution in Symbiodiniaceae cultures and isolates. Cell cycle percentages are provided for strains of *B. minutum* and *B. psygmophilum* species under different conditions. All numbers represent averaged cell cycle percentages of cultures and isolates as determined by the Dean-Jet-Fox model (*n* = 3). Numbers in parentheses represent standard deviations. Download Table S2, DOCX file, 0.02 MB. Copyright © 2020 Tivey et al. 2020 Tivey et al. This content is distributed under the terms of the Creative Commons Attribution 4.0 International license.

### Fluorescent labeling of host Aiptasia during recolonization by symbionts.

Previously bleached, aposymbiotic H2 anemones were plated in 24-well plates and inoculated with *B. minutum* (strain Mf1.05b) at a concentration of 1 × 10^5^ cells in 1 ml FSW for 2 days and then moved to fresh plates containing new FSW. Hosts exhibited a low level of symbiont recolonization for 2 months, after which symbiont population growth accelerated. Hosts were monitored for 2 weeks of rapid symbiont growth until recolonization reached 50% of the tentacle area. Hosts were then incubated in 10 μM EdU in FSW for 24 h to measure host cell proliferation (Click-iT EdU Alexa Fluor 555 imaging kit; Life Technologies, Eugene, OR, USA), as EdU binds to replicating DNA only in S phase (Fig. 1A).

In pilot work, shorter EdU incubations were explored to determine S-phase duration in the epidermis and gastrodermis. To visualize cell populations that had exited S phase and entered G_2_/M phase, tentacles incubated in EdU were also labeled with anti-pSer10-H3 conjugated to Alexa Fluor 488. In a 4-h EdU incubation treatment, there were limited S-phase populations within the gastrodermis, limited G_2_/M-phase populations within the epidermis, and no double labeling of individual cells. In 6-h incubations, only a few epidermal nuclei in confocal tentacle sections exhibited double labeling (less than 5% of G_2_/M-phase labeled cells). The highest cell cycle rate from S phase to G_2_/M phase for any host cell population in the tentacle was therefore estimated to be between 4 and 6 h. Given the long duration of this transition, and the fact that M and G_1_ phases need to occur before another S-phase cycle, confounding effects from subsequent rounds of S-phase incorporation were deemed unlikely within a 24-h time span. Thus, we chose to sample anemones after 24-h EdU incubation to examine all proliferating gastrodermal cells, not just those cells undergoing DNA replication at the time of EdU addition. We were not concerned about accidentally labeling replicating symbiont cells, as pilot tests showed that the Click-iT EdU azide AF555 did not label EdU-incubated Symbiodiniaceae cell cultures, likely due to their thick cell walls and nonoptimal conditions for the Click-IT EdU reaction.

Whole polyps destined for host cell proliferation visualization (Fig. 1B) were rinsed in phosphate-buffered saline (1× PBS) prepared from a 10× PBS stock solution (0.02 M NaH_2_PO_4_, 0.077 M Na_2_PO_4_, 1.4 M NaCl, pH 7.4) and fixed in 1× PBS + 4% paraformaldehyde overnight at 4°C. Samples were then rinsed in 1× PBS and blocked in 1× PBST (0.05% Triton X-100) for 30 min. Tentacles were incubated for 1 h in the reaction mixture of the Click-iT EdU Alexa Fluor 555 imaging kit, which labeled only proliferating (EdU^+^) host nuclei. Next, the nuclei of all host cells (both proliferating and nonproliferating) were labeled with Hoechst 33342, which did not penetrate symbiont cells. Finally, tentacles were washed three times in 1× PBS and mounted on slides for confocal microscopy (Prolong antifade diamond mountant; Life Technologies) (Fig. 1C). Samples were imaged on a Zeiss LSM 780 NLO confocal microscope system. Symbiodiniaceae chlorophyll autofluorescence was detected using excitation at 633 nm and an emission of 684 nm (Fig. 1D). All host nuclei labeled with Hoechst strain were detected with excitation at 405 nm and an emission of 443 nm (Fig. 1A and E). Proliferating host nuclei labeled with EdU-AF555 were excited at 555 nm and an emission of 588 nm (Fig. 1A and F). Overlap between emission channels was avoided by using EdU-treated samples with and without Click-iT AF555, Hoechst stain, and symbiont autofluorescence (Fig. S1). Confocal z-stack images were analyzed using Fiji (ImageJ2) (101, 102). In total, 179 confocal images were analyzed (*n* = 3 tentacles per anemone; *n* = 11 symbiotic anemones; *n* = 11 aposymbiotic anemones).

10.1128/mBio.02626-19.1 FIG S1 Fluorescence channel controls used for confocal microscopy. Aposymbiotic and symbiotic tentacles were used in combination with fluorescent channel controls to set excitation and emission channels for host proliferation and location analysis. The four channels and their composite image are displayed in columns: algal autofluorescence, EdU-AF555, Hoechst stain, bright-field, and composite. EdU labeling without AF555 is shown in row 1, with no noticeable background labeling. EdU + AF555 is shown in row 2 and is observed only in the EdU-AF555 channel and the composite. Hoechst stain labeling is shown in row 3 and is present only in its respective Hoechst channel and the composite. Autofluorescent symbionts from an unlabeled symbiotic tentacle are shown in row 4 and are noticeable in both their respective channel and bright field, as well as the composite. Finally, row 5 shows a sample z-stack projection from the tentacle analysis performed in this study. Download FIG S1, JPG file, 1.7 MB. Copyright © 2020 Tivey et al. 2020 Tivey et al. This content is distributed under the terms of the Creative Commons Attribution 4.0 International license.

An image analysis pipeline was developed to quantify the relationship between symbionts and regions of host tissue containing proliferating host nuclei in tentacles (Fig. 2A). Images were split into separate channels, and the three-dimensional (3D) object counter plugin (103) was used to detect the centers of mass for all host nuclei (Hoechst positive) and proliferating host nuclei (EdU-AF555 positive) (Fig. 2B). Likewise, *B. minutum* cells were detected with the same plugin after smoothing objects using a Gaussian blur and background subtraction. The *B. minutum* cells were subjected to two sequential rounds of object segmentation using 3D Watershed that separated overlapping symbiont cell clusters into objects approximating single cells (104). All symbiont objects were then recounted, and their centers of mass were identified. The densities of total host nuclei, proliferating host nuclei, and symbionts were then determined by dividing the number of nuclei by the total tentacle volume similarly to previously used methods (47, 48, 62, 63). To verify that possible differences in proliferative host cell number between treatments did not rely on differences in total tentacle volume, the percentages of total proliferating nuclei were determined by dividing EdU^+^ populations by total host nuclei. Average host cell sizes were estimated by dividing tentacle volume by the number of nuclei and converting to cell diameter.

The Euclidean distances from each symbiont object in a tentacle to its *k*th-nearest neighbor (*k *= 1 to 12) of either all host nuclei or proliferating host nuclei were measured using the R package *spatstat* (105) (Fig. 2C). These nearest-neighbor (NN) distances were then aggregated by host cell type, and two-sample *t* tests were used to determine differences in their distributions (Fig. 2D). To determine whether the observation of larger median NN distances between proliferating and nonproliferating cells in symbiotic anemones relative to aposymbiotic anemones was a result of a change in the proliferation rate in epidermal versus gastrodermal tissue layers, the depths of all cells were examined using the *z* coordinates of each object. For each tentacle, the median *z*-coordinate location of each cell population was calculated and visualized in a density plot. To delineate tissue layers at different depths, the “deeper” peak of the Symbiodiniaceae cell population location was used to approximate the gastrodermal layer, as this is where most symbionts reside. The “shallower” peak of the all-host-cell population location that did not overlap the Symbiodiniaceae peak was used to approximate the epidermis. The median proliferating-host-cell population location was normalized to the median all-host-cell population location for each tentacle, and shifts in the proliferating cell distribution between symbiotic and aposymbiotic anemones were assessed via two-sample *t* test.

As a separate spatial analysis, the locations of symbionts were used to divide the tentacles areas into aposymbiotic and symbiotic regions as follows. After separating symbiont clusters into single cell objects (Fig. 2E), symbiont-containing cell regions were estimated using a minimum bounding box: the smallest three-dimensional box that encloses all of a given symbiont object. The minimum bounding box regions of these symbionts were aggregated and compared to the spatial location of host nucleus centers of mass (Fig. 2F). A Boolean test was used on the *x*, *y*, and *z* coordinates of host nuclei to determine whether they were found inside or outside the bounding box regions containing *B. minutum* cells. To estimate *B. minutum* density, the total volume of these symbiont bounding box regions was calculated and compared to the total volume of each tentacle. Regions of symbiont bounding box overlap were subtracted to avoid overestimation of symbiont density.

### Symbiodiniaceae cell cycle dynamics under different nutritional and symbiosis states.

To assess the effect of nutritional state on Symbiodiniaceae cell cycles *ex hospite*, cultured *B. minutum* strain FLAp2 and cultured *B. psygmophilum* strain HIAp were sampled under stable growth conditions (after 12 weeks in f/2 medium at 1 × 10^6^ cells/ml) or during predicted log-phase growth (after 2 weeks in f/2 medium at 2.5 × 10^5^ cells/ml). To assess nutritional effects *in hospite*, symbiotic polyps of host Aiptasia strain H2 harboring *B. minutum* and host Aiptasia strain JK harboring *B. psygmophilum* were starved or fed, as previously described, for 2 weeks prior to symbiont isolation. To obtain Symbiodiniaceae cells from host anemones, polyps were homogenized in FSW using a BioSpec Tissue-Tearor and centrifuged at 500 × *g* for 5 min to pellet the algae. Pellets were rinsed in 1× PBS twice, forced through a 22-gauge needle, and fixed in 2 ml of ice-cold 70% ethanol, which was slowly added as samples were vortexed. Samples were kept at 4°C for up to 1 week, and then 1 ml of ice-cold 70% ethanol was added and samples were photobleached for 1 h under high light (43, 61). To stain DNA for flow cytometry analysis, samples were rinsed in 1× PBS prior to staining in 500 ml of 1× PBS with 10 μg/ml propidium iodide (PI) (Fig. 1A), 0.01% Triton X-100, and 150 μg/ml RNase A. The cells were analyzed using the CytoFLEX 4-liter flow cytometer at a flow rate of 10 μl/min. Cells were excited at 488 nm and detected using a 585/42 band-pass filter (phycoerythrin [PE] channel). Symbiodiniaceae populations were isolated from cnidarian cell populations using forward and side scatter. Cells were screened for doublet discrimination using forward scatter (FSC)-height and FSC-width channels to avoid overestimating G_2_/M-phase populations (Fig. S2). At least 20,000 cells were collected per sample. Symbiodiniaceae are strongly autofluorescent, and therefore, it was necessary to ensure that autofluorescence was not having a confounding effect on PI labeling in the PE channel. Unlabeled controls of cultures and isolates were used to determine successful photobleaching and labeling of algal cell populations (Fig. S3). Cell cycle analysis was performed using FlowJo software v10 (FlowJo, LLC). Cell cycle gates were determined using the Dean-Jett-Fox model in order to best model S-phase distribution. Once cell proportions of G_1_, S, and G_2_/M phases were identified from each sample, the data were arcsine transformed to stabilize variance. A two-way analysis of variance (ANOVA) was used to identify differences between nutritional and symbiotic states, followed by a Tukey *post hoc* test. All statistical analyses were performed in R.

10.1128/mBio.02626-19.2 FIG S2 Symbiodiniaceae gating. Cultures and isolates were first gated using forward scatter (FSC height × FSC width) to avoid overestimating G_2_-phase cell populations. Gates are drawn around the border of G_1_-phase algal populations which were confirmed via relative genomic DNA content. Doublet populations were located directly above gated populations and confirmed to contain twice the DNA content of the G_1_-phase population from the FSC-H FSC-W gates. Note that forward scatter increased for nitrogen-depleted treatments. Download FIG S2, TIF file, 1.9 MB. Copyright © 2020 Tivey et al. 2020 Tivey et al. This content is distributed under the terms of the Creative Commons Attribution 4.0 International license.

10.1128/mBio.02626-19.3 FIG S3 Flow cytometric controls for Symbiodiniaceae cell populations. Unlabeled Symbiodiniaceae cells were used to assess successful bleaching of chlorophyll and propidium iodide labeling of samples. The plots shown have been previously gated using forward scatter height and width (FSC-H and FSC-W) for doublet discrimination. PE-A is shown on the *x* axis, indicating propidium iodide fluorescence intensity. FSC-width is shown on the *y* axis to verify single populations. Unlabeled cells of both Symbiodiniaceae species cultures and of an anemone isolate had PE fluorescence intensity levels below levels of propidium iodide labeling (dashed line). In addition, negative controls did not display multiple cell populations, indicating relatively uniform photobleaching. Download FIG S3, TIF file, 2.9 MB. Copyright © 2020 Tivey et al. 2020 Tivey et al. This content is distributed under the terms of the Creative Commons Attribution 4.0 International license.

### Data availability.

All image processing pipelines, scripts, and statistical analyses are available in the supplemental material as Data Set S1 and online at GitHub (https://github.com/trtivey).

10.1128/mBio.02626-19.6 DATA SET S1 Data and scripts for image processing and data analysis. Tables for flow data analysis are provided for each Symbiodiniaceae comparison experiment. Scripts are included for Fiji/ImageJ macros to find fluorescent markers in tentacle z stacks and create objects in 3D space. Rmarkdown scripts are included for subsequent data analysis and figure generation. Data files that were used with these scripts can be found at GitHub (https://github.com/trtivey). Download Data Set S1, DOCX file, 0.1 MB. Copyright © 2020 Tivey et al. 2020 Tivey et al. This content is distributed under the terms of the Creative Commons Attribution 4.0 International license.
