# Supplementary material for: Host and Symbiont Cell Cycle Coordination Is Mediated by Symbiotic State, Nutrition, and Partner Identity in a Model Cnidarian-Dinoflagellate Symbiosis
Source: mBio. 2020 Mar 10;11(2):e02626-19. doi: 10.1128/mBio.02626-19 (PMC7064764; doi:10.1128/mBio.02626-19)
Supplement: TABLE S1 [file mBio.02626-19-st001.docx]

Table S1: Aiptasia and Symbiodiniaceae strain information

| **Species/Strain** | **ITS2 Type of symbiont** | **Source** |  |  |
| --- | --- | --- | --- | --- |
| ***E. pallida*** |  |  |  |  |
| H2 | B1 | Collected at Coconut Island in Kaneohe Bay, HI |  |  |
| JK | B2 | Population collected at Wilmington, NC in 2016 |  |  |
| VWA12 | B1 | Clone from population collected from an aquarium in Corvallis, OR |  |  |
| VWB9 | B1 | Clone from population collected at Coconut Island in Kaneohe Bay, HI |  |  |
| ***B. minutum*** |  |  |  |  |
| CCMP830 | B1 | *Exaiptasia pallida* isolate collected at Bermuda Biological Station, Bermuda |  |  |
| FLAp2 | B1 | *Exaiptasia pallida* isolate collected at Long Key, FL |  |  |
| Mf1.05b | B1 | *Orbicella faveolata* isolate collected at the Florida Keys, Florida |  |  |
| ***B. psygmophilum*** |  |  |  |  |
| HIAp | B2 | *Exaiptasia pallida* isolate collected at Kaneohe Bay, Hawaii |  |  |
|  |  |  |  |  |
|  |  |  |  |  |
