# Supplementary material for: Host and Symbiont Cell Cycle Coordination Is Mediated by Symbiotic State, Nutrition, and Partner Identity in a Model Cnidarian-Dinoflagellate Symbiosis
Source: mBio. 2020 Mar 10;11(2):e02626-19. doi: 10.1128/mBio.02626-19 (PMC7064764; doi:10.1128/mBio.02626-19)
Supplement: TABLE S2 [file mBio.02626-19-st002.docx]

Table S2: Cell cycle distribution in Symbiodiniaceae cultures and isolates

|  |  |  |  |  | **Log-Growth Phase** | | | **Stationary Phase** | | |
| --- | --- | --- | --- | --- | --- | --- | --- | --- | --- | --- |
| **Species Name** | **ITS2**  **Type** | **Strain (ID)** | **Source Organism** | **Culture or isolate** | **G1**  **(%)** | **S**  **(%)** | **G2**  **(%)** | **G1**  **(%)** | **S**  **(%)** | **G2**  **(%)** |
| *B. minutum* | B1 | CCMP 830 | *Exaiptasia pallida* | Culture | 73.4  (2.3) | 10.0  (2.3) | 14.4  (0.7) | 93.6  (1.1) | 3.7  (0.8) | 2.3  (0.3) |
|  |  | Ap2 | *Exaiptasia pallida* | Culture | 79.5  (0.7) | 4.1  (0.7) | 15.4  (0.3) | 93.6  (0.3) | 2.2  (1.3) | 2.1  (1.9) |
|  |  | FLAp2 | *Exaiptasia pallida* | Culture | 76.0  (1.3) | 8.4  (1.0) | 14.9  (0.5) | 94.2  (3.1) | 4.1  (2.1) | 0.7  (0.6) |
|  |  | Mf 1.05b | *Orbicella faveolata* | Culture | 89.3  (6.4) | 2.8  (1.2) | 7.4  (7.1) | 94.6  (0.2) | 3.2  (2.7) | 0.1  (0.1) |
| *B. psygmophilum* | B2 | HIAp | *Exaiptasia pallida* | Culture | 81.1  (3.5) | 7.5  (3.0) | 11.1  (2.9) | 96.0  (0.4) | 1.4  (0.2) | 2.5  (0.4) |
|  |  |  |  |  | **Nitrogen-replete** | | | **Nitrogen-limited** | | |
| *B. minutum* | B1 | Mf 1.05b | *Orbicella faveolata* | Culture | 98.7  (1.1) | 1.9  (0.5) | 0.8  (0.3) | 94.3  (1.0) | 3.6  (0.2) | 0.0  (0.0) |
| *B. psygmophilum* | B2 | HIAp | *Exaiptasia pallida* | Culture | 91  (1.6) | 2.9  (0.6) | 2.5  (1.1) | 81.2  (2.2) | 19.3  (1.5) | 0.4  (0.1) |
|  |  |  |  |  | **In Symbiosis** | | |  |  |  |
| *B. minutum* | B1 | H2 isolate | *Exaiptasia pallida* | Freshly isolated | 72.0  (4.3) | 21.2  (5.24) | 0.8  (0.3) |  |  |  |
|  |  | VWA12 isolate | *Exaiptasia pallida* | Freshly isolated | 77.9  (11.9) | 16.6  (7.8) | 3.7  (3.1) |  |  |  |
|  |  | VWB9 isolate | *Exaiptasia pallida* | Freshly isolated | 82.2  (2.2) | 13.0  (3.2) | 4.0  (0.6) |  |  |  |
| *B. psygmophilum* | B2 | JK isolate | *Exaiptasia pallida* | Freshly isolated | 54.1  (7.2) | 36.5  (7.7) | 2.3  (0.7) |  |  |  |
|  |  |  |  |  |  |  |  |  |  |  |
|  |  |  |  |  |  |  |  |  |  |  |

All numbers represent averaged cell cycle percentages of cultures and isolates as determined by the Dean Jet Fox model (n = 3). Numbers in parentheses represent standard deviation.
