# Supplementary figures and images for: Host and Symbiont Cell Cycle Coordination Is Mediated by Symbiotic State, Nutrition, and Partner Identity in a Model Cnidarian-Dinoflagellate Symbiosis
Source: mBio. 2020 Mar 10;11(2):e02626-19. doi: 10.1128/mBio.02626-19 (PMC7064764; doi:10.1128/mBio.02626-19)

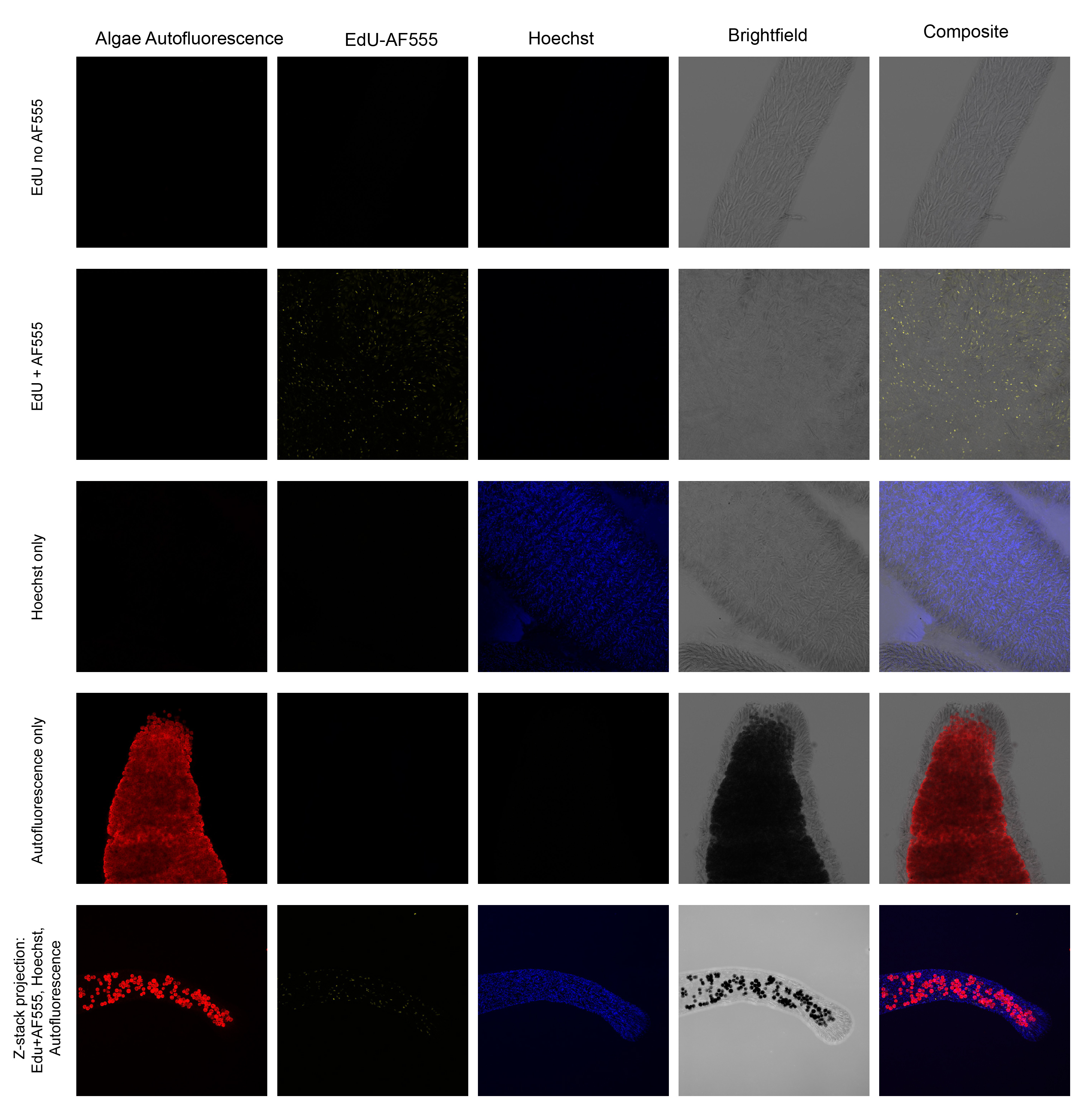

Supplement: FIG S1 [file mBio.02626-19-sf001.jpg]

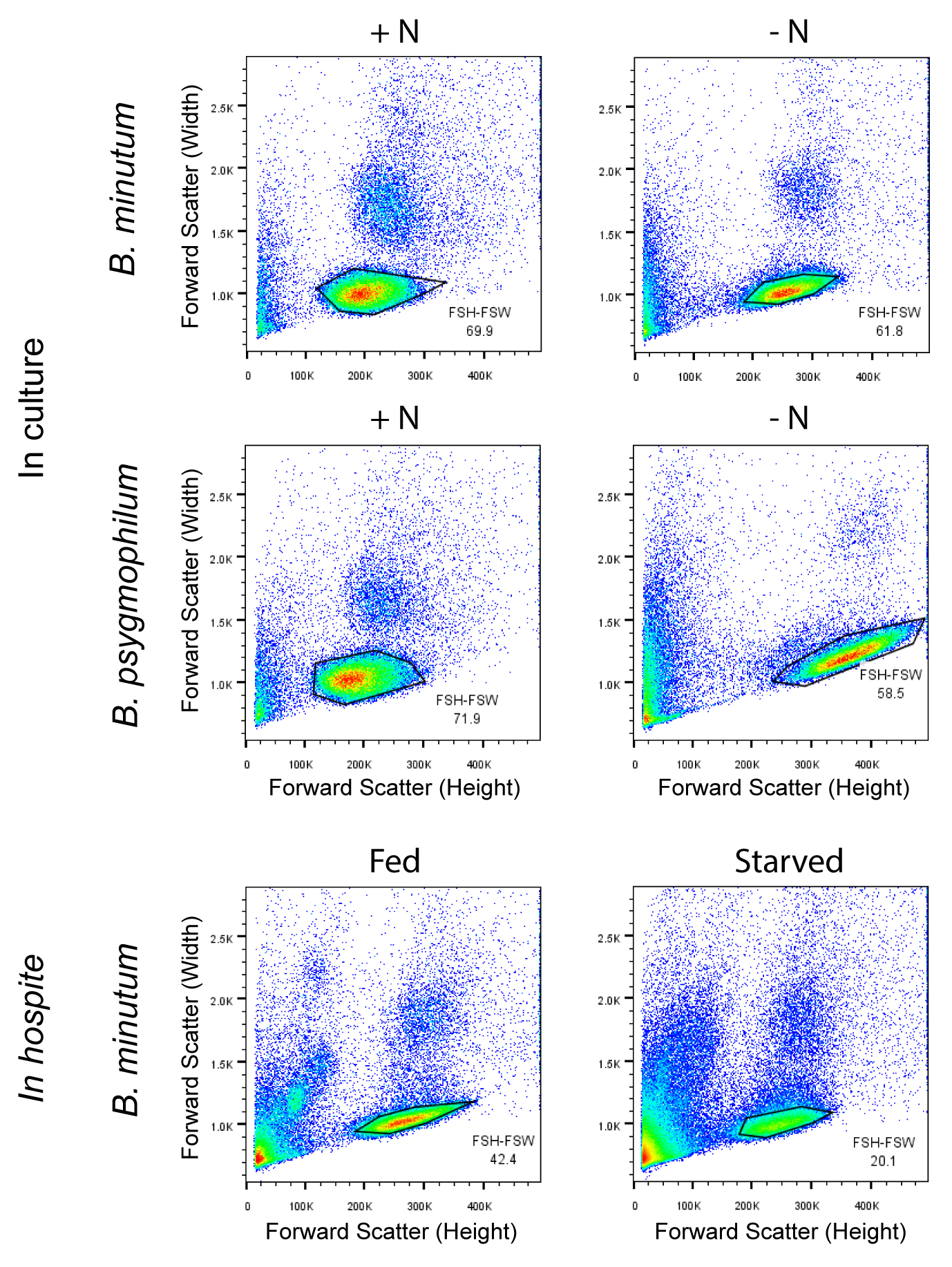

Supplement: FIG S2 [file mBio.02626-19-sf002.tif]

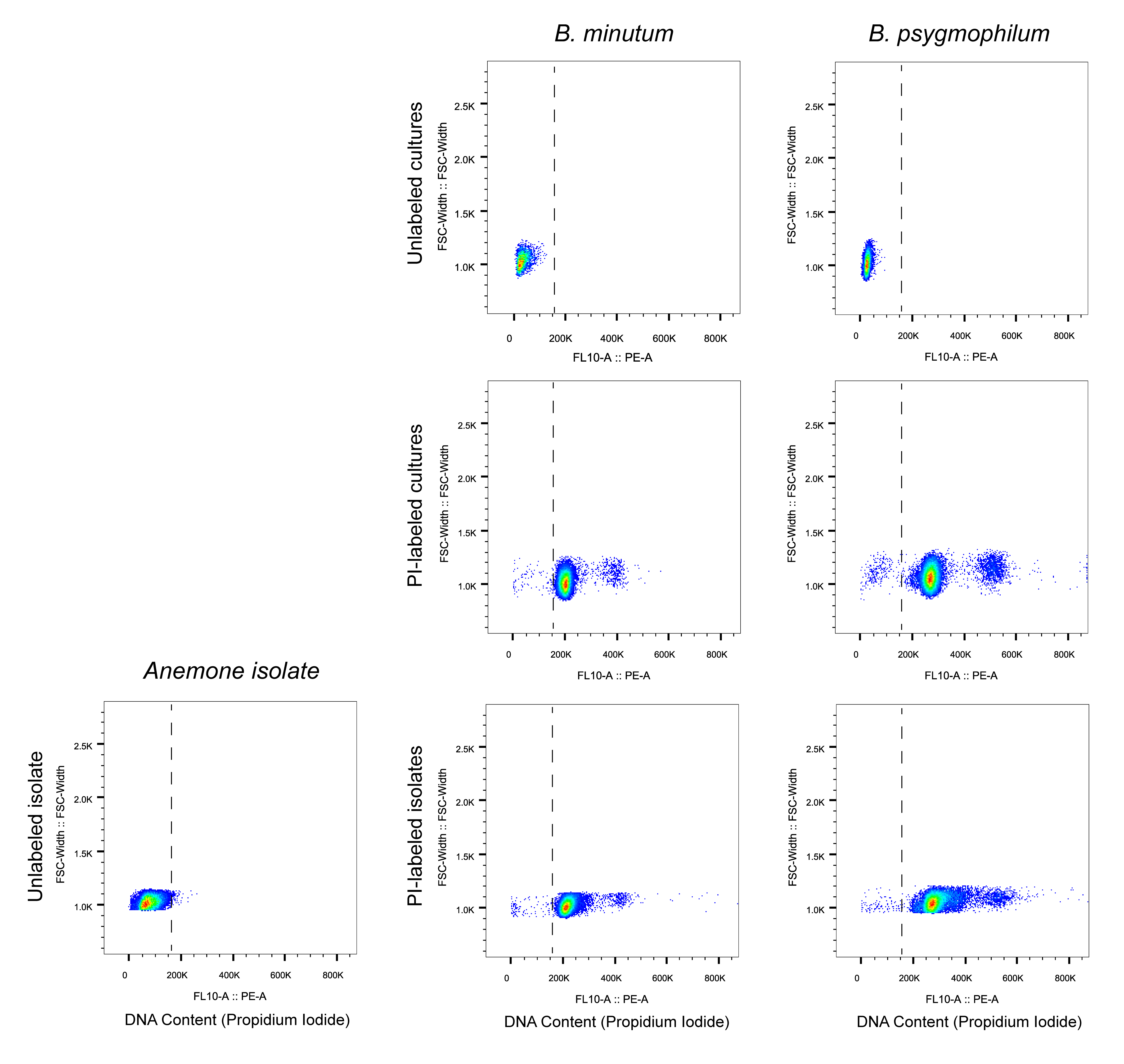

Supplement: FIG S3 [file mBio.02626-19-sf003.tif]
