# Supplementary material for: Host and Symbiont Cell Cycle Coordination Is Mediated by Symbiotic State, Nutrition, and Partner Identity in a Model Cnidarian-Dinoflagellate Symbiosis
Source: mBio. 2020 Mar 10;11(2):e02626-19. doi: 10.1128/mBio.02626-19 (PMC7064764; doi:10.1128/mBio.02626-19)
Supplement: DATA SET S1 [file mBio.02626-19-sd001.docx]

**Data S1**

**Data and scripts for image processing and data analysis**

**Flow data analysis 2**

*B. minutum* stable vs. log-growth 2

*B. psygmophilum* stable vs. log-growth 2

*B. minutum* and *B. psygmophilum* *in hospite* 2

*B. minutum* fed and starved *in hospite* 2

Nitrogen comparisons (+N. -N in culture) 3

**FIJI/ImageJ macro scripts for finding 3D objects 4**

3d_obj_count_3dvolume.ijm 4

3d_obj_count_edu.ijm 5

3d_obj_count_hoechst.ijm 7

symbiont_part_1_gaussian_blur_3d_object_count.ijm 8

symbiont_part_2_segmentation.ijm 10

symbiont_part_2_segmentation.ijm 10

symbiont_part_4_final_3d_object_count.ijm 11

**Rmarkdown scripts for image and data analysis 12**

symbiont_flow_analysis 12

NN_list_apo 15

NN_list_sym 17

Spatial_analyses_plots 21

Spatial_analyses_symbiont_location 35

Symbiont_bounding_box_analysis 46

*B. minutum* stable vs. log-growth

| **Species** | **Strain** | **Treatment** | **G1**  **(%)** | **G1**  **(pop)** | **S (%)** | **S**  **(pop)** | **G2 (%)** | **G2**  **(pop)** |  |  | **G1**  **(%)** | **G1**  **(pop)** | **S**  **(%)** | **S**  **(pop)** | **G2**  **(%)** | **G2**  **(pop)** |
| --- | --- | --- | --- | --- | --- | --- | --- | --- | --- | --- | --- | --- | --- | --- | --- | --- |
| B. minutum | FLAp2 | log-phase | 74.7 | 35350 | 9.19 | 4345 | 15.4 | 7290 |  | **Average (log-phase)** | 76 | 35994 | 8.36 | 3958 | 14.9 | 7058 |
| B. minutum | FLAp2 | log-phase | 76.1 | 36020 | 8.64 | 4091 | 14.5 | 6846 |  | **Average (stable)** | 94.2 | 40910 | 4.14 | 1807 | 0.67 | 294 |
| B. minutum | FLAp2 | log-phase | 77.2 | 36612 | 7.25 | 3439 | 14.8 | 7039 |  |  |  |  |  |  |  |  |
| B. minutum | FLAp2 | stable | 97.7 | 41299 | 1.87 | 790 | 0 | 0 |  | **t-test** | 0.0007 | 0.008 | 0.035 | 0.022 | 5.2E-06 | 4.3E-06 |
| B. minutum | FLAp2 | stable | 91.7 | 39152 | 6.05 | 2585 | 1.14 | 486 |  | **fold difference** | 1.24 | 1.14 | 0.495 | 0.457 | 0.045 | 0.042 |
| B. minutum | FLAp2 | stable | 93.2 | 42280 | 4.51 | 2046 | 0.87 | 395 |  | **fold difference** | 0.807 | 0.880 | 2.017 | 2.19 | 22.23 | 24.03 |

*B. psygmophilum* stable vs. log-growth

| **Species** | **Strain** | **Treatment** | **G1 (%)** | **G1 (pop)** | **S (%)** | **S**  **(pop)** | **G2 (%)** | **G2 (pop)** |  |  | **G1**  **(%)** | **G1**  **(pop)** | **S**  **(%)** | **S**  **(pop)** | **G2**  **(%)** | **G2**  **(pop)** |
| --- | --- | --- | --- | --- | --- | --- | --- | --- | --- | --- | --- | --- | --- | --- | --- | --- |
| B. psygmophilum | HIAp | log-phase | 84.3 | 43874 | 7.51 | 3911 | 7.89 | 4108 |  | **Average (log-phase)** | 81.1 | 41741 | 7.46 | 3848 | 11.06 | 5673 |
| B. psygmophilum | HIAp | log-phase | 77.3 | 39588 | 10.4 | 5350 | 11.8 | 6024 |  | **Average (stable)** | 96 | 71167 | 1.37 | 1016 | 2.52 | 1836 |
| B. psygmophilum | HIAp | log-phase | 81.7 | 41762 | 4.46 | 2282 | 13.5 | 6888 |  |  |  |  |  |  |  |  |
| B. psygmophilum | HIAp | stable | 96.4 | 83938 | 1.47 | 1279 | 2.07 | 1799 |  | **t-test** | 0.002 | 0.012 | 0.024 | 0.034 | 0.007 | 0.010 |
| B. psygmophilum | HIAp | stable | 96 | 67915 | 1.17 | 827 | 2.61 | 1844 |  | **fold difference** | 1.18 | 1.70 | 0.18 | 0.26 | 0.23 | 0.33 |
| B. psygmophilum | HIAp | stable | 95.6 | 61649 | 1.46 | 941 | 2.89 | 1866 |  | **fold difference** | 0.84 | 0.59 | 5.466 | 3.79 | 4.39 | 3.09 |

*B. minutum* and *B. psygmophilum* *in hospite*

| **Species** | **Strain** | **Treatment** | **G1 (%)** | **S (%)** | **G2 (%)** | **G1 CV** |  |  | **G1**  **(%)** | **S**  **(%)** | **G2**  **(%)** | **G1 CV** |
| --- | --- | --- | --- | --- | --- | --- | --- | --- | --- | --- | --- | --- |
| B. minutum | H2 | fresh isolate (in hospite) | 74.8 | 18.5 | 0.45 | 5.46 |  | **Average (log-phase)** | 72 | 21.18 | 0.757 | 5.49 |
| B. minutum | H2 | fresh isolate (in hospite) | 74.1 | 17.8 | 0.92 | 5.94 |  | **Average (stable)** | 54.13 | 36.47 | 2.25 | 6.88 |
| B. minutum | H2 | fresh isolate (in hospite) | 67.1 | 27.2 | 0.9 | 5.06 |  |  |  |  |  |  |
| B. psygmophilum | JK | fresh isolate (in hospite) | 61.9 | 28 | 1.68 | 7.46 |  | **t-test** | 0.021 | 0.046 | 0.0228 | 0.039 |
| B. psygmophilum | JK | fresh isolate (in hospite) | 47.8 | 42.9 | 2.1 | 7.04 |  | **fold difference** | 0.75 | 1.72 | 2.97 | 1.25 |
| B. psygmophilum | JK | fresh isolate (in hospite) | 52.7 | 38.5 | 2.97 | 6.15 |  | **fold difference** | 1.33 | 0.58 | 0.34 | 0.80 |

*B. minutum* fed and starved *in hospite*

| **Species** | **Strain** | **Treatment** | **G1 (%)** | **S (%)** | **G2 (%)** |  |  | **G1**  **(%)** | **S**  **(%)** | **G2**  **(%)** |
| --- | --- | --- | --- | --- | --- | --- | --- | --- | --- | --- |
| B. minutum | H2 | Fed | 73.6 | 17 | 0.91 |  | **Average (Fed)** | 72.83 | 18.22 | 1.45 |
| B. minutum | H2 | Fed | 74.5 | 15.3 | 1.68 |  | **Average (Starved)** | 85.2 | 11.25 | 0.93 |
| B. minutum | H2 | Fed | 70.4 | 22.4 | 1.76 |  |  |  |  |  |
| B. minutum | H2 | Starved | 82.9 | 13.5 | 1.23 |  | **t-test** | 0.002 | 0.047 | 0.18 |
| B. minutum | H2 | Starved | 86.9 | 9.25 | 0.62 |  | **fold difference** | 1.17 | 0.62 | 0.64 |
| B. minutum | H2 | Starved | 85.8 | 11 | 0.94 |  | **fold difference** | 0.85 | 1.62 | 1.56 |

Nitrogen comparisons (+N. -N in culture)

| **Species** | **Strain** | **Treatment** | **rms** | **%G1** | **%S** | **%G2** | **% <G1** | **%> G2** | **g1cv** |
| --- | --- | --- | --- | --- | --- | --- | --- | --- | --- |
| B. minutum | Mf1.05b | Nitrogen | 10.9 | 98.4 | 2.45 | 0.58 | 1.62 | -0.013 | 6.52 |
| B. minutum | Mf1.05b | Nitrogen | 10.8 | 100 | 1.5 | 0.63 | 0.84 | -0.0088 | 6.72 |
| B. minutum | Mf1.05b | Nitrogen | 11.3 | 97.8 | 1.86 | 1.1 | 2.4 | -0.019 | 5.97 |
| B. minutum | Mf1.05b | No Nitrogen | 5.61 | 93.8 | 3.57 | 0.02 | -0.51 | 0.0046 | 9.13 |
| B. minutum | Mf1.05b | No Nitrogen | 5.42 | 93.6 | 3.88 | 0.047 | -0.73 | -0.012 | 8.67 |
| B. minutum | Mf1.05b | No Nitrogen | 6.13 | 95.5 | 3.39 | 0.038 | -1.65 | -0.019 | 9.16 |
|  |  |  |  |  |  |  |  |  |  |
| **Species** | **Strain** | **Treatment** | **rms** | **%G1** | **%S** | **%G2** | **% <G1** | **%> G2** | **g1cv** |
| B. psygmophilum | HIAp | Nitrogen | 3.66 | 91.5 | 2.94 | 1.37 | 2.89 | 0.18 | 5.22 |
| B. psygmophilum | HIAp | Nitrogen | 7.85 | 91.7 | 2.25 | 2.49 | 4.53 | 0.036 | 6.94 |
| B. psygmophilum | HIAp | Nitrogen | 4 | 88.8 | 3.43 | 3.6 | 3.07 | 0.31 | 5.46 |
| B. psygmophilum | HIAp | No Nitrogen | 3.4 | 78.8 | 21 | 0.29 | -0.59 | -0.043 | 13.9 |
| B. psygmophilum | HIAp | No Nitrogen | 3.84 | 83 | 18.1 | 0.47 | -1.48 | -0.13 | 14.2 |
| B. psygmophilum | HIAp | No Nitrogen | 3.62 | 81.9 | 18.8 | 0.45 | -1.72 | -0.085 | 14.2 |

|  | **B. minutum** | **G1 (%)** | **S (%)** | **G2 (%)** | **G1 CV** |
| --- | --- | --- | --- | --- | --- |
| **t-test** | N v No N | 0.008 | 0.006 | 0.011 | 0.0007 |
|  |  |  |  |  |  |
|  |  |  |  |  |  |
|  | **B. psygmophilum** | **G1 (%)** | **S (%)** | **G2 (%)** | **G1 CV** |
| **t-test** | N v No N | 0.004 | 6.25E-05 | 0.032 | 0.0001 |
|  |  |  |  |  |  |
|  | **B. minutum** | **G1** | **S** | **G2** | **G1 CV** |
| **Average** | Nitrogen | 98.73 | 1.94 | 0.77 | 6.40 |
| **Average** | No Nitrogen | 94.3 | 3.61 | 0.035 | 8.99 |
|  | Fold Change | 1.05 | 1.87 | 22 | 1.40 |
|  |  |  |  |  |  |
|  | **B. psygmophilum** | **G1** | **S** | **G2** | **G1 CV** |
| **Average** | Nitrogen | 90.67 | 2.87 | 2.49 | 5.87 |
| **Average** | No Nitrogen | 81.23 | 19.3 | 0.40 | 14.1 |
|  | Fold Change | 1.12 | 6.72 | 6.17 | 2.40 |

**FIJI/ImageJ macro scripts for finding 3D objects**

**3d_obj_count_3dvolume.ijm**

input = "/Users/ttivey/Desktop/DataAnalysis/sym/";

output = "/Users/ttivey/Desktop/DataAnalysis/sym_volume/";

setBatchMode(true);

list = getFileList(input);

for (i = 0; i <list.length; i++)

action(input, output, list[i]);

setBatchMode(false);

function action(input, output, filename) {

open(input + filename);

run("Split Channels");

//hoecsht channel 5

close();

// EdU channel 4

close();

// Brightfield

run("3D Objects Counter", "threshold=1000 slice=1 min.=10 statistics summary");

selectWindow("Results");

saveAs("Text", output + filename);

run("Close");

//algal channel

close();

selectWindow("Log");

saveAs("Text", output + "/volume-log.txt");

}

**3d_obj_count_edu.ijm**

input = "/Users/ttivey/Desktop/DataAnalysis/apo/";

output = "/Users/ttivey/Desktop/DataAnalysis/apoout/";

setBatchMode(true);

list = getFileList(input);

for (i = 0; i <list.length; i++)

action(input, output, list[i]);

setBatchMode(false);

function action(input, output, filename) {

open(input + filename);

run("Split Channels");

//hoecsht channel

close();

//EdU channel

run("3D Objects Counter", "slice=1 min.=10 max.=500 statistics summary centroids surfaces centres_of_masses objects");

selectWindow("Results");

saveAs("Text", output + filename);

run("Close");

selectWindow("Centroids map of C3-" + filename);

t=getTitle;

saveAs("Tiff", output + t);

selectWindow("Centroids map of C3-" + filename);

close();

selectWindow("Centres of mass map of C3-" + filename);

t=getTitle;

saveAs("Tiff", output + t);

selectWindow("Centres of mass map of C3-" + filename);

close();

selectWindow("Surface map of C3-" + filename);

t=getTitle;

saveAs("Tiff", output + t);

selectWindow("Surface map of C3-" + filename);

close();

selectWindow("Objects map of C3-" + filename);

t=getTitle;

saveAs("Tiff", output + t);

selectWindow("Objects map of C3-" + filename);

close();

// Brightfield channel

close();

// Algal channel

close();

selectWindow("Log");

saveAs("Text", output + "/edu-log.txt");

}

**3d_obj_count_hoechst.ijm**

input = "/Users/ttivey/Desktop/DataAnalysis/apo/";

output = "/Users/ttivey/Desktop/DataAnalysis/apoout/";

setBatchMode(true);

list = getFileList(input);

for (i = 0; i <list.length; i++)

action(input, output, list[i]);

setBatchMode(false);

function action(input, output, filename) {

open(input + filename);

run("Split Channels");

//hoecsht channel 5

run("3D Objects Counter", "slice=1 min.=5 max.=500 statistics summary centres_of_masses objects");

selectWindow("Results");

saveAs("Text", output + filename);

run("Close");

selectWindow("Centres of mass map of C4-" + filename);

t=getTitle;

saveAs("Tiff", output + t);

selectWindow("Centres of mass map of C4-" + filename);

close();

selectWindow("Objects map of C4-" + filename);

t=getTitle;

saveAs("Tiff", output + t);

selectWindow("Objects map of C4-" + filename);

close();

//EdU channel

close();

//brightfield

close();

//last algal channel

close();

selectWindow("Log");

saveAs("Text", output + "/hoechst-log.txt");

}

**symbiont_part_1_gaussian_blur_3d_object_count.ijm**

input = "/Users/ttivey/Desktop/DataAnalysis/sym/";

output = "/Users/ttivey/Desktop/DataAnalysis/symc1out/";

setBatchMode(true);

list = getFileList(input);

for (i = 0; i <list.length; i++)

action(input, output, list[i]);

setBatchMode(false);

function action(input, output, filename) {

open(input + filename);

run("Split Channels");

//hoecsht channel 5

close();

//edu

close();

//brightfield

close();

//gaussian blur

run("Gaussian Blur 3D...", "x=1 y=1 z=1");

//subtract background

run("Subtract Background...", "rolling=50 sliding stack");

//symbiont channel 4

run("3D Objects Counter", "slice=1 min.=150 statistics summary centroids surfaces centres_of_masses objects");

selectWindow("Results");

saveAs("Text", output + filename);

run("Close");

selectWindow("Centroids map of C1-" + filename);

t=getTitle;

saveAs("Tiff", output + t);

selectWindow("Centroids map of C1-" + filename);

close();

selectWindow("Centres of mass map of C1-" + filename);

t=getTitle;

saveAs("Tiff", output + t);

selectWindow("Centres of mass map of C1-" + filename);

close();

selectWindow("Surface map of C1-" + filename);

t=getTitle;

saveAs("Tiff", output + t);

selectWindow("Surface map of C1-" + filename);

close();

selectWindow("Objects map of C1-" + filename);

t=getTitle;

saveAs("Tiff", output + t);

selectWindow("Objects map of C1-" + filename);

close();

selectWindow("Log");

saveAs("Text", output + "/edu-log.txt");

}

**symbiont_part_2_segmentation.ijm**

input = "/Users/ttivey/Desktop/DataAnalysis/sym_c1_step2_subset_objects_in/";

output = "/Users/ttivey/Desktop/DataAnalysis/sym_c1_watershed1_out/";

setBatchMode(true);

list = getFileList(input);

for (i = 0; i <list.length; i++)

action(input, output, list[i]);

setBatchMode(false);

function action(input, output, filename) {

open(input + filename);

run("3D Watershed Split", "binary=Objects seeds=Automatic radius=5");

selectWindow("Split");

//run("3D Watershed Split", "binary=Split seeds=Automatic radius=5");

saveAs("Tiff", output + filename + "_2");

run("Close All");

}

**symbiont_part_3_segmentation.ijm**

input = "/Users/ttivey/Desktop/DataAnalysis/sym_c1_watershed1_out/";

output = "/Users/ttivey/Desktop/DataAnalysis/sym_watershed2/";

setBatchMode(true);

list = getFileList(input);

for (i = 0; i <list.length; i++)

action(input, output, list[i]);

setBatchMode(false);

function action(input, output, filename) {

open(input + filename);

run("3D Watershed Split", "binary=Objects seeds=Automatic radius=5");

selectWindow("Split");

//run("3D Watershed Split", "binary=Split seeds=Automatic radius=5");

saveAs("Tiff", output + filename + "_2");

run("Close All");

}

**symbiont_part_4_final_3d_object_count.ijm**

input = "/Users/ttivey/Desktop/DataAnalysis/sym_watershed2/";

output = "/Users/ttivey/Desktop/DataAnalysis/sym_c1_final/";

setBatchMode(true);

list = getFileList(input);

for (i = 0; i <list.length; i++)

action(input, output, list[i]);

setBatchMode(false);

function action(input, output, filename) {

open(input + filename);

//algae

run("3D Objects Counter", "threshold=1 slice=1 min.=150 statistics summary centroids surfaces centres_of_masses objects");

selectWindow("Results");

saveAs("Text", output + filename);

run("Close");

selectWindow("Centroids map of " + filename);

t=getTitle;

saveAs("Tiff", output + t);

selectWindow("Centroids map of " + filename);

close();

selectWindow("Centres of mass map of " + filename);

t=getTitle;

saveAs("Tiff", output + t);

selectWindow("Centres of mass map of " + filename);

close();

selectWindow("Surface map of " + filename);

t=getTitle;

saveAs("Tiff", output + t);

selectWindow("Surface map of " + filename);

close();

selectWindow("Objects map of " + filename);

t=getTitle;

saveAs("Tiff", output + t);

selectWindow("Objects map of " + filename);

close();

selectWindow("Log");

saveAs("Text", output + "/symbiont-log.txt");

}

**Rmarkdown scripts for image and data analysis**

**symbiont_flow_analysis.rmd**

---

title: "symbiont_flow_analysis"

author: "Trevor Tivey"

date: "12/19/2019"

output: html_document

---

```{r setup, include=FALSE}

knitr::opts_chunk$set(echo = TRUE)

#setwd("/local/path/to/data")

set.seed(123)

#libraries

library(ggplot2)

library(ggsignif)

```

## Plots

```{r, plots, echo = FALSE}

# Plot for Bmin Bpsyg log-phase culture, stable culture, and isolates

BminBpsyg <- read.csv("BminBpsyg.csv", header = T)

BminBpsyg$Phase <- factor(BminBpsyg$Phase, levels = c("G2", "S", "G1"))

BminBpsyg$Treatment <- factor(BminBpsyg$Treatment, levels = c(" In hospite"," Stationary", " Log-phase","In hospite","Stationary","Log-phase"))

g <- ggplot() + geom_bar(aes(y = Number, x = Treatment, group = Species, fill = Phase), data = BminBpsyg, stat = "identity")

k = g + coord_flip() + theme_minimal() + labs(x = "" , y = "Cell cycle population percentage") + scale_fill_brewer(palette = "RdYlBu")

k

ggsave(plot=k, filename="fig5A.pdf", width=5, height=5, units="in")

# Plot for Bmin and Bpsyg under different Nitrogen conditions

nitrogen <- read.csv("nitrogen.csv", header = T)

nitrogen$Phase <- factor(nitrogen$Phase, levels = c("G2", "S", "G1"))

nitrogen$Treatment <- factor(nitrogen$Treatment, levels = c(" N-limited "," N-replete ", "N-limited ","N-replete"))

h <- ggplot() + geom_bar(aes(y = Number, x = Treatment, group = Species, fill = Phase), data = nitrogen, stat = "identity")

j <- h + coord_flip() + theme_minimal() + labs(x = "" , y = "Cell cycle population percentage") + scale_fill_brewer(palette = "RdYlBu")

j

ggsave(plot=j, filename="fig6A.pdf", width=5, height=3.5, units="in")

# Plot for Bmin cell cycle isolated within fed and starved host anemones

fedstarveccfig <- read.csv("fedstarvecellcyclefigure.csv", header = T)

fedstarveccfig$Phase <- factor(fedstarveccfig$Phase, levels = c("G1","S","G2"))

l <- ggplot(aes(Phase, Average, color = Treatment, shape = Treatment), data = fedstarveccfig) +

geom_errorbar(aes(ymin = Average - Stdev, ymax = Average + Stdev, width = 0.2),

color = "dark grey", position = position_dodge(width = 0.3)) +

geom_point(size = 3, position = position_dodge(width = 0.3)) +

theme_minimal() + labs(title = "", x = " ", y = " ") +

theme(axis.text.x = element_text(angle=0, size=12), axis.text.y = element_text(angle=0, size=12)) +

scale_color_manual(values=c("#d8b365", "#998ec3")) +

scale_shape_manual(values=c(19,15))

l

ggsave(plot=l, filename="fig6C.eps", width=4, height=5, units="in")

```

**NN_list_apo.rmd**

---

title: "NN_list_apo"

author: "Trevor Tivey"

date: "2/20/2019"

output: html_document

---

```{r, libraries, include=FALSE}

knitr::opts_chunk$set(echo = TRUE)

#setwd("/local/path/to/data")

set.seed(123)

#libraries

library(data.table)

library(ggplot2)

library(spatstat)

library(plyr)

library(dplyr)

```

## create lists

```{r, apo lists, echo = FALSE}

# Set directory to folder containing x,y,z coordinates of cell populations

setwd("./input apo")

# Create subset lists that are organized in corresponding order to each other

# Files containing object coordinates of host EdU-labeled nuclei

edulist = list.files(pattern = " [1-3].txt")

edulist

# Files containing object coordinates of all host nuclei (Hoechst-labeled)

nuclist = list.files(pattern = "_2.txt")

nuclist

```

## Loop for apos

You can also embed plots, for example:

```{r, apo for loop, echo=FALSE}

# Loop to find k=1-12 nearest neighbors between symbionts and edu, symbionts and nuclei, and edu and nuclei

for(i in 1:length(edulist)) {

setwd("./input apo")

edu <- read.csv(edulist[i], header = T, sep = "\t")

nuclei <- read.csv(nuclist[i], header = T, sep = "\t")

filenmEN <- sub("txt", "csv", edulist)

print(filenmEN[i])

filenmNS <- sub("txt", "csv", nuclist)

print(filenmNS[i])

nucleix <- range(nuclei[,11])

nucleiy <- range(nuclei[,12])

nucleiz <- range(nuclei[,13])

pnuclei <- ppp(nuclei[,11],nuclei[,12], nucleix, nucleiy)

pnuclei3 <- pp3(nuclei[,11],nuclei[,12],nuclei[,13],nucleix, nucleiy, nucleiz)

edux <- range(edu[,12])

eduy <- range(edu[,13])

eduz <- range(edu[,14])

pedu <- ppp(edu[,12],edu[,13], nucleix, nucleiy)

pedu3 <- pp3(edu[,12],edu[,13],edu[,14],nucleix, nucleiy, nucleiz)

edunuclei <- nncross(pedu3,pnuclei3,k=1:12)

nucleiedu <- nncross(pnuclei3,pedu3,k=1:12)

edunuclei2d <- nncross(pedu,pnuclei,k=1:12)

nucleiedu2d <- nncross(pnuclei,pedu,k=1:12)

setwd("../output apo/3d nn test")

write.csv(edunuclei, filenmEN[i])

write.csv(nucleiedu, filenmNS[i])

setwd("../2d nn test")

write.csv(edunuclei2d, filenmEN[i])

write.csv(nucleiedu2d, filenmNS[i])

setwd("../..")

}

```

**NN_list_sym.rmd**

---

title: "NN_list_sym"

author: "Trevor Tivey"

date: "2/20/2019"

output: html_document

---

```{r, libraries, include=FALSE}

knitr::opts_chunk$set(echo = TRUE)

#setwd("/local/path/to/data")

set.seed(123)

#libraries

library(data.table)

library(ggplot2)

library(spatstat)

library(plyr)

library(dplyr)

```

## create lists

```{r, apo lists, echo = FALSE}

# Set directory to folder containing x,y,z coordinates of cell populations

setwd("./input sym")

# Create subset lists that are organized in corresponding order to each other

# Files containing object coordinates of all symbiont cluster centers of mass (autofluorescence)

s_symlist = list.files(pattern = "C1-")

s_symlist

# Files containing object coordinates of host EdU-labeled nuclei

s_edulist = list.files(pattern = " [1-3].txt")

s_edulist

# Files containing object coordinates of host nuclei (Hoechst labeled)

s_nuclist = list.files(pattern = "C4-")

s_nuclist

```

## Loop for apos

You can also embed plots, for example:

```{r, apo for loop, echo=FALSE}

# Loop to find k=1-12 nearest neighbors between symbionts and edu, symbionts and nuclei, and edu and nuclei

#loop to find k=12 nearest neighbors between symbionts and edu, symbionts and nuclei, and edu and nuclei

for(i in 1:length(s_edulist)) {

setwd("./input sym")

s_edu <- read.csv(s_edulist[i], header = T, sep = "\t")

s_symbionts <- read.csv(s_symlist[i], header = T, sep = "\t")

s_nuclei <- read.csv(s_nuclist[i], header = T, sep = "\t")

s_filenmEN <- sub("txt", "csv", s_edulist)

print(s_filenmEN[i])

s_filenmES <- sub("txt", "csv", s_symlist)

print(s_filenmES[i])

s_filenmNS <- sub("txt", "csv", s_nuclist)

print(s_filenmNS[i])

s_nucleix <- range(s_nuclei[,11])

s_nucleiy <- range(s_nuclei[,12])

s_nucleiz <- range(s_nuclei[,13])

s_pnuclei <- ppp(s_nuclei[,11], s_nuclei[,12], s_nucleix, s_nucleiy)

s_pnuclei3 <- pp3(s_nuclei[,11], s_nuclei[,12], s_nuclei[,13], s_nucleix, s_nucleiy, s_nucleiz)

s_symbiontsx <- range(s_symbionts[,12])

s_symbiontsy <- range(s_symbionts[,13])

s_symbiontsz <- range(s_symbionts[,14])

s_psymbionts <- ppp(s_symbionts[,12], s_symbionts[,13], s_nucleix, s_nucleiy)

s_psymbionts3 <- pp3(s_symbionts[,12], s_symbionts[,13], s_symbionts[,14], s_nucleix, s_nucleiy, s_nucleiz)

s_edux <- range(s_edu[,12])

s_eduy <- range(s_edu[,13])

s_eduz <- range(s_edu[,14])

s_pedu <- ppp(s_edu[,12], s_edu[,13], s_nucleix, s_nucleiy)

s_pedu3 <- pp3(s_edu[,12], s_edu[,13], s_edu[,14], s_nucleix, s_nucleiy, s_nucleiz)

s_edunuclei <- nncross(s_pedu3, s_pnuclei3, k=1:12)

s_edusym <- nncross(s_pedu3, s_psymbionts3, k=1:12)

s_nucsym <- nncross(s_pnuclei3, s_psymbionts3, k=1:12)

s_nucleiedu <- nncross(s_pnuclei3, s_pedu3, k=1:12)

s_symedu <- nncross(s_psymbionts3, s_pedu3, k=1:12)

s_symnuc <- nncross(s_psymbionts3, s_pnuclei3, k=1:12)

s_edunuclei2D <- nncross(s_pedu, s_pnuclei, k=1:12)

s_edusym2D <- nncross(s_pedu, s_psymbionts, k=1:12)

s_nucsym2D <- nncross(s_pnuclei, s_psymbionts, k=1:12)

s_nucleiedu2D <- nncross(s_pnuclei, s_pedu, k=1:12)

s_symedu2D <- nncross(s_psymbionts, s_pedu, k=1:12)

s_symnuc2D <- nncross(s_psymbionts, s_pnuclei, k=1:12)

setwd("../output sym/3d nn test")

write.csv(s_edunuclei, s_filenmEN[i])

write.csv(s_edusym, s_filenmES[i])

write.csv(s_nucsym, s_filenmNS[i])

setwd("../3d nn test reverse")

write.csv(s_nucleiedu, s_filenmEN[i])

write.csv(s_symedu, s_filenmES[i])

write.csv(s_symnuc, s_filenmNS[i])

setwd("../2d nn test")

write.csv(s_edunuclei2D, s_filenmEN[i])

write.csv(s_edusym2D, s_filenmES[i])

write.csv(s_nucsym2D, s_filenmNS[i])

setwd("../2d nn test reverse")

write.csv(s_nucleiedu2D, s_filenmEN[i])

write.csv(s_symedu2D, s_filenmES[i])

write.csv(s_symnuc2D, s_filenmNS[i])

setwd("../..")

}

```

**Spatial_analyses_plots.rmd**

---

title: "Spatial_analyses_plots"

author: "Trevor Tivey"

date: "2/20/2019"

output: html_document

---

```{r, libraries, include=FALSE}

knitr::opts_chunk$set(echo = TRUE)

#setwd("/local/path/to/data")

set.seed(123)

#libraries

library(data.table)

library(ggplot2)

library(spatstat)

library(plyr)

library(dplyr)

library(tidyr)

#library(aacc)

```

## Create list and data table of NN distances for aposymbiotic tentacles

```{r, apo lists, echo = FALSE}

# create list of NN csv files and combine into one large data frame

setwd("./apo_analysis/output apo/3d nn test")

#files containing NN distances of all host EdU+ nuclei to closest total nuclei

edulistapo2 = list.files(pattern = " [1-3].csv")

edulistapo2

# Create list

eduapofiles = lapply(edulistapo2, read.csv, header=T, stringsAsFactors = F)

# Create data table

eduapofiles2 <- rbindlist(eduapofiles, fill = T)

colnames(eduapofiles2) <- paste("enapo", colnames(eduapofiles2), sep = "_")

```

## Create list and data frames of NN distances for symbiotic tentacles

```{r, apo lists, echo = FALSE}

# Create list of NN csv files and combine into three large data frames for each comparison:

# 1. EdU points to nearest symbiont

# 2. Nuclei points to nearest symbiont

# 3. EdU points to nearest nuclei

setwd("./sym_analysis/output sym/3d nn test")

# lists

#files containing NN dists between host EdU+ nuclei and symbionts

edusymlist2 = list.files(pattern = "split.tif_2.csv")

edusymlist2

#files containing object coordinates of total nuclei and symbionts

nucsymlist2 = list.files(pattern = " [1-3].tif_2.csv")

nucsymlist2

#files containing object coordinates of host EdU+ nuclei and total nuclei

edunucslist2 = list.files(pattern = " [1-3].csv")

edunucslist2

# apply lists and create data tables

# Distances from EdU to symbionts

edusymfiles = lapply(edusymlist2, read.csv, header=T, stringsAsFactors = F)

edusymfiles2 <- rbindlist(edusymfiles, fill = T)

colnames(edusymfiles2) <- paste("es", colnames(edusymfiles2), sep = "_")

# Distances from nuclei to symbionts

nucsymfiles = lapply(nucsymlist2, read.csv, header=T, stringsAsFactors = F)

nucsymfiles2 <- rbindlist(nucsymfiles, fill = T)

colnames(nucsymfiles2) <- paste("ns", colnames(nucsymfiles2), sep = "_")

# Distances from EdU to nuclei

edunucsfiles = lapply(edunucslist2, read.csv, header=T, stringsAsFactors = F)

edunucsfiles2 <- rbindlist(edunucsfiles, fill = T)

colnames(edunucsfiles2) <- paste("en", colnames(edunucsfiles2), sep = "_")

```

## Test NN distance distribution for normality

```{r, apo lists, echo = FALSE}

# test for normality

qqnorm(edusymfiles2$es_dist.1)

qqline(edusymfiles2$es_dist.1)

qqnorm(nucsymfiles2$ns_dist.1)

qqline(nucsymfiles2$ns_dist.1)

qqnorm(edunucsfiles2$en_dist.1)

qqline(edunucsfiles2$en_dist.1)

qqnorm(edusymfiles2$es_dist.12)

qqline(edusymfiles2$es_dist.12)

qqnorm(nucsymfiles2$ns_dist.12)

qqline(nucsymfiles2$ns_dist.12)

qqnorm(edunucsfiles2$en_dist.12)

qqline(edunucsfiles2$en_dist.12)

qqnorm(eduapofiles2$enapo_dist.1)

qqline(eduapofiles2$enapo_dist.1)

qqnorm(eduapofiles2$enapo_dist.12)

qqline(eduapofiles2$enapo_dist.12)

# Skew in data, analyze distribution using nonparametric tests

```

## Combine all files into a single NN file for comparison

```{r, apo lists, echo = FALSE}

# Combine files

combinedfiles <- cbind(edusymfiles2, edunucsfiles2)

len <- max(nrow(combinedfiles), nrow(nucsymfiles2))

#len#delete

#nrow(combinedfiles) #delete

#nrow(nucsymfiles2)#delete

nrow(nucsymfiles2) - nrow(combinedfiles) # difference is 207834

temprow <- matrix(c(rep.int(NA,length(combinedfiles))),nrow=207834,ncol=length(combinedfiles))

# Create a data.frame and give cols the same names as data

newrow <- data.frame(temprow)

colnames(newrow) <- colnames(combinedfiles)

# rbind the empty row to data

combinedsamelength <- rbind(combinedfiles,newrow)

combinedfiles2 <- cbind(combinedsamelength, nucsymfiles2)

#combinedfiles <- cbind(combinedfiles, nucfiles2) #delete

## Combine apo and sym lists

len <- max(nrow(eduapofiles2), nrow(combinedfiles2))

#len#delete

#nrow(eduapofiles2) #delete

#nrow(combinedfiles2)#delete

nrow(combinedfiles2) - nrow(eduapofiles2) # difference is 207653

temprow <- matrix(c(rep.int(NA,length(eduapofiles2))),nrow=207653,ncol=length(eduapofiles2))

# Create a data.frame and give cols the same names as data

newrow <- data.frame(temprow)

colnames(newrow) <- colnames(eduapofiles2)

# rbind the empty row to data

eduapofiles2samelengthapo <- rbind(eduapofiles2,newrow)

combined_nn <- cbind(eduapofiles2samelengthapo, combinedfiles2)

#combinedfiles <- cbind(combinedfiles, nucfiles2)#delete

```

## Fig 4D and 3A: Mann-Whitney U tests: is distance of EdU -> Symbiont greater than EdU --> nuclei

```{r, apo lists, echo = FALSE}

# Comparison between EdU-->symbiont distance and EdU-->host nuclei distance distributions

# EdU to symbiont distance is greater than EdU to host nuclei distances

wilcox.test(x = combined_nn$es_dist.1, y = combined_nn$en_dist.1, data = combined_nn, alternative = "greater")

wilcox.test(x = combined_nn$es_dist.2, y = combined_nn$en_dist.2, data = combined_nn, alternative = "greater")

wilcox.test(x = combined_nn$es_dist.3, y = combined_nn$en_dist.3, data = combined_nn, alternative = "greater")

wilcox.test(x = combined_nn$es_dist.4, y = combined_nn$en_dist.4, data = combined_nn, alternative = "greater")

wilcox.test(x = combined_nn$es_dist.5, y = combined_nn$en_dist.5, data = combined_nn, alternative = "greater")

wilcox.test(x = combined_nn$es_dist.6, y = combined_nn$en_dist.6, data = combined_nn, alternative = "greater")

wilcox.test(x = combined_nn$es_dist.7, y = combined_nn$en_dist.7, data = combined_nn, alternative = "greater")

wilcox.test(x = combined_nn$es_dist.8, y = combined_nn$en_dist.8, data = combined_nn, alternative = "greater")

wilcox.test(x = combined_nn$es_dist.9, y = combined_nn$en_dist.9, data = combined_nn, alternative = "greater")

wilcox.test(x = combined_nn$es_dist.10, y = combined_nn$en_dist.10, data = combined_nn, alternative = "greater")

wilcox.test(x = combined_nn$es_dist.11, y = combined_nn$en_dist.11, data = combined_nn, alternative = "greater")

wilcox.test(x = combined_nn$es_dist.12, y = combined_nn$en_dist.12, data = combined_nn, alternative = "greater")

t.test(x = combined_nn$es_dist.1, y = combined_nn$en_dist.1, data = combined_nn)

t.test(x = combined_nn$es_dist.2, y = combined_nn$en_dist.2, data = combined_nn, alternative = "greater")

wilcox.test(x = combined_nn$es_dist.3, y = combined_nn$en_dist.3, data = combined_nn, alternative = "greater")

wilcox.test(x = combined_nn$es_dist.4, y = combined_nn$en_dist.4, data = combined_nn, alternative = "greater")

wilcox.test(x = combined_nn$es_dist.5, y = combined_nn$en_dist.5, data = combined_nn, alternative = "greater")

wilcox.test(x = combined_nn$es_dist.6, y = combined_nn$en_dist.6, data = combined_nn, alternative = "greater")

wilcox.test(x = combined_nn$es_dist.7, y = combined_nn$en_dist.7, data = combined_nn, alternative = "greater")

wilcox.test(x = combined_nn$es_dist.8, y = combined_nn$en_dist.8, data = combined_nn, alternative = "greater")

wilcox.test(x = combined_nn$es_dist.9, y = combined_nn$en_dist.9, data = combined_nn, alternative = "greater")

wilcox.test(x = combined_nn$es_dist.10, y = combined_nn$en_dist.10, data = combined_nn, alternative = "greater")

wilcox.test(x = combined_nn$es_dist.11, y = combined_nn$en_dist.11, data = combined_nn, alternative = "greater")

wilcox.test(x = combined_nn$es_dist.12, y = combined_nn$en_dist.12, data = combined_nn, alternative = "greater")

```

## Fig 3A: Mann-Whitney U tests: is distance of EdU -> Symbiont less than Nuclei -> symbiont

```{r, apo lists, echo = FALSE}

# Comparison between EdU-->symbiont distance and nuclei-->symbiont distance distributions

# EdU to symbiont distance is less than nuclei to symbiont distances

wilcox.test(x = combined_nn$es_dist.1, y = combined_nn$ns_dist.1, data = combined_nn, alternative = "less", conf.int = T)

wilcox.test(x = combined_nn$es_dist.2, y = combined_nn$ns_dist.2, data = combined_nn, alternative = "less", conf.int = T)

wilcox.test(x = combined_nn$es_dist.3, y = combined_nn$ns_dist.3, data = combined_nn, alternative = "less", conf.int = T)

wilcox.test(x = combined_nn$es_dist.4, y = combined_nn$ns_dist.4, data = combined_nn, alternative = "less", conf.int = T)

wilcox.test(x = combined_nn$es_dist.5, y = combined_nn$ns_dist.5, data = combined_nn, alternative = "less", conf.int = T)

wilcox.test(x = combined_nn$es_dist.6, y = combined_nn$ns_dist.6, data = combined_nn, alternative = "less", conf.int = T)

wilcox.test(x = combined_nn$es_dist.7, y = combined_nn$ns_dist.7, data = combined_nn, alternative = "less", conf.int = T)

wilcox.test(x = combined_nn$es_dist.8, y = combined_nn$ns_dist.8, data = combined_nn, alternative = "less", conf.int = T)

wilcox.test(x = combined_nn$es_dist.9, y = combined_nn$ns_dist.9, data = combined_nn, alternative = "less", conf.int = T)

wilcox.test(x = combined_nn$es_dist.10, y = combined_nn$ns_dist.10, data = combined_nn, alternative = "less", conf.int = T)

wilcox.test(x = combined_nn$es_dist.11, y = combined_nn$ns_dist.11, data = combined_nn, alternative = "less", conf.int = T)

wilcox.test(x = combined_nn$es_dist.12, y = combined_nn$ns_dist.12, data = combined_nn, alternative = "less", conf.int = T)

# All highly significant differences between distributions. EdU objects are much further away to symbionts than to host nuclei, as expected.

# Positive control

# All tests indicate significant location shift in distributions.

# All tests indicate edu-symbiont distance is less than nuclei-symbiont distance.

```

# Fig 3C: Medians of NN dists, Percentages of EdU+ inside median distance to symbiont

```{r, apo lists, echo = FALSE}

# Find medians of NN distributions for EdU-symbiont and Nuc-symbiont

median(combined_nn$es_dist.1, na.rm = T)

median(combined_nn$ns_dist.1, na.rm = T)

median(combined_nn$es_dist.2, na.rm = T)

median(combined_nn$ns_dist.2, na.rm = T)

median(combined_nn$es_dist.3, na.rm = T)

median(combined_nn$ns_dist.3, na.rm = T)

median(combined_nn$es_dist.4, na.rm = T)

median(combined_nn$ns_dist.4, na.rm = T)

median(combined_nn$es_dist.5, na.rm = T)

median(combined_nn$ns_dist.5, na.rm = T)

median(combined_nn$es_dist.6, na.rm = T)

median(combined_nn$ns_dist.6, na.rm = T)

median(combined_nn$es_dist.7, na.rm = T)

median(combined_nn$ns_dist.7, na.rm = T)

median(combined_nn$es_dist.8, na.rm = T)

median(combined_nn$ns_dist.8, na.rm = T)

median(combined_nn$es_dist.9, na.rm = T)

median(combined_nn$ns_dist.9, na.rm = T)

median(combined_nn$es_dist.10, na.rm = T)

median(combined_nn$ns_dist.10, na.rm = T)

median(combined_nn$es_dist.11, na.rm = T)

median(combined_nn$ns_dist.11, na.rm = T)

median(combined_nn$es_dist.12, na.rm = T)

median(combined_nn$ns_dist.12, na.rm = T)

# Estimate percentage of EdU+ host nuclei inside median distance to symbiont (7 µm)

count_edu_total <- subset(combined_nn$es_dist.1, combined_nn$es_dist.1 > 0)

count_edu_in <- subset(combined_nn$es_dist.1, combined_nn$es_dist.1 < 7)

count_edu_out <- subset(combined_nn$es_dist.1, combined_nn$es_dist.1 > 7)

perc_edu_in <- length(count_edu_in)/length(count_edu_total)

perc_edu_out <-length(count_edu_out)/length(count_edu_total)

perc_edu_in

perc_edu_out

# Estimate percentage of total host nuclei inside median distance to symbiont (7 µm)

count_nuc_total <- subset(combined_nn$ns_dist.1, combined_nn$ns_dist.1 > 0)

count_nuc_in <- subset(combined_nn$ns_dist.1, combined_nn$ns_dist.1 < 7)

count_nuc_out <- subset(combined_nn$ns_dist.1, combined_nn$ns_dist.1 > 7)

perc_nuc_in <- length(count_nuc_in)/length(count_nuc_total)

perc_nuc_out <-length(count_nuc_out)/length(count_nuc_total)

perc_nuc_in

perc_nuc_out

# Estimate percentage of total host nuclei inside distance to symbiont (8 µm)

count_edu_total <- subset(combined_nn$es_dist.1, combined_nn$es_dist.1 > 0)

count_edu_in <- subset(combined_nn$es_dist.1, combined_nn$es_dist.1 < 8)

count_edu_out <- subset(combined_nn$es_dist.1, combined_nn$es_dist.1 > 8)

perc_edu_in <- length(count_edu_in)/length(count_edu_total)

perc_edu_out <-length(count_edu_out)/length(count_edu_total)

perc_edu_in

perc_edu_out

# Estimate percentage of total host nuclei inside distance to symbiont (8 µm)

count_nuc_total <- subset(combined_nn$ns_dist.1, combined_nn$ns_dist.1 > 0)

count_nuc_in <- subset(combined_nn$ns_dist.1, combined_nn$ns_dist.1 < 8)

count_nuc_out <- subset(combined_nn$ns_dist.1, combined_nn$ns_dist.1 > 8)

perc_nuc_in <- length(count_nuc_in)/length(count_nuc_total)

perc_nuc_out <-length(count_nuc_out)/length(count_nuc_total)

perc_nuc_in

perc_nuc_out

count_edu_total <- subset(combined_nn$es_dist.1, combined_nn$es_dist.1 > 0)

count_edu_in <- subset(combined_nn$es_dist.1, combined_nn$es_dist.1 < 13)

count_edu_out <- subset(combined_nn$es_dist.1, combined_nn$es_dist.1 > 13)

perc_edu_in <- length(count_edu_in)/length(count_edu_total)

perc_edu_out <-length(count_edu_out)/length(count_edu_total)

perc_edu_in

perc_edu_out

count_nuc_total <- subset(combined_nn$ns_dist.1, combined_nn$ns_dist.1 > 0)

count_nuc_in <- subset(combined_nn$ns_dist.1, combined_nn$ns_dist.1 < 13)

count_nuc_out <- subset(combined_nn$ns_dist.1, combined_nn$ns_dist.1 > 13)

perc_nuc_in <- length(count_nuc_in)/length(count_nuc_total)

perc_nuc_out <-length(count_nuc_out)/length(count_nuc_total)

perc_nuc_in

perc_nuc_out

# Estimate percentage of total host nuclei inside distance to symbiont (30 µm)

count_edu_total <- subset(combined_nn$es_dist.1, combined_nn$es_dist.1 > 0)

count_edu_in <- subset(combined_nn$es_dist.1, combined_nn$es_dist.1 < 30)

count_edu_out <- subset(combined_nn$es_dist.1, combined_nn$es_dist.1 > 30)

perc_edu_in <- length(count_edu_in)/length(count_edu_total)

perc_edu_out <-length(count_edu_out)/length(count_edu_total)

perc_edu_in

perc_edu_out

# Estimate percentage of total host nuclei inside distance to symbiont (30 µm)

count_nuc_total <- subset(combined_nn$ns_dist.1, combined_nn$ns_dist.1 > 0)

count_nuc_in <- subset(combined_nn$ns_dist.1, combined_nn$ns_dist.1 < 30)

count_nuc_out <- subset(combined_nn$ns_dist.1, combined_nn$ns_dist.1 > 30)

perc_nuc_in <- length(count_nuc_in)/length(count_nuc_total)

perc_nuc_out <-length(count_nuc_out)/length(count_nuc_total)

perc_nuc_in

perc_nuc_out

```

# Fig 3B t-test comparisons

```{r, apo lists, echo = FALSE}

# t-tests describing the means of distributions (previously used Mann-Whitney to find differences between same distributions)

t.test(x = combined_nn$es_dist.1, y = combined_nn$ns_dist.1, data = combined_nn)

t.test(x = combined_nn$es_dist.2, y = combined_nn$ns_dist.2, data = combined_nn)

t.test(x = combined_nn$es_dist.3, y = combined_nn$ns_dist.3, data = combined_nn)

t.test(x = combined_nn$es_dist.4, y = combined_nn$ns_dist.4, data = combined_nn)

t.test(x = combined_nn$es_dist.5, y = combined_nn$ns_dist.5, data = combined_nn)

t.test(x = combined_nn$es_dist.6, y = combined_nn$ns_dist.6, data = combined_nn)

t.test(x = combined_nn$es_dist.7, y = combined_nn$ns_dist.7, data = combined_nn)

t.test(x = combined_nn$es_dist.8, y = combined_nn$ns_dist.8, data = combined_nn)

t.test(x = combined_nn$es_dist.9, y = combined_nn$ns_dist.9, data = combined_nn)

t.test(x = combined_nn$es_dist.10, y = combined_nn$ns_dist.10, data = combined_nn)

t.test(x = combined_nn$es_dist.11, y = combined_nn$ns_dist.11, data = combined_nn)

t.test(x = combined_nn$es_dist.12, y = combined_nn$ns_dist.12, data = combined_nn)

```

## Fig 4E Mann-Whitney U tests: is distance of EdU -> nuclei shorter in apos compared to syms

```{r, apo lists, echo = FALSE}

## Wilcox test for compared NN distances between apos and syms

# Test to show 1st nearest neighbor has a smaller distance than 2nd nearest neighbor.

wilcox.test(x = combined_nn$enapo_dist.1, y = combined_nn$enapo_dist.2, data = combined_nn, alternative = "less")

# Test differences between magnitude of NN distances at k = 1,2,...,12

wilcox.test(x = combined_nn$enapo_dist.1, y = combined_nn$en_dist.1, data = combined_nn, alternative = "less", conf.int = T)

wilcox.test(x = combined_nn$enapo_dist.2, y = combined_nn$en_dist.2, data = combined_nn, alternative = "less", conf.int = T)

wilcox.test(x = combined_nn$enapo_dist.3, y = combined_nn$en_dist.3, data = combined_nn, alternative = "less", conf.int = T)

wilcox.test(x = combined_nn$enapo_dist.4, y = combined_nn$en_dist.4, data = combined_nn, alternative = "less", conf.int = T)

wilcox.test(x = combined_nn$enapo_dist.5, y = combined_nn$en_dist.5, data = combined_nn, alternative = "less", conf.int = T)

wilcox.test(x = combined_nn$enapo_dist.6, y = combined_nn$en_dist.6, data = combined_nn, alternative = "less", conf.int = T)

wilcox.test(x = combined_nn$enapo_dist.7, y = combined_nn$en_dist.7, data = combined_nn, alternative = "less", conf.int = T)

wilcox.test(x = combined_nn$enapo_dist.8, y = combined_nn$en_dist.8, data = combined_nn, alternative = "less", conf.int = T)

wilcox.test(x = combined_nn$enapo_dist.9, y = combined_nn$en_dist.9, data = combined_nn, alternative = "less", conf.int = T)

wilcox.test(x = combined_nn$enapo_dist.10, y = combined_nn$en_dist.10, data = combined_nn, alternative = "less", conf.int = T)

wilcox.test(x = combined_nn$enapo_dist.11, y = combined_nn$en_dist.11, data = combined_nn, alternative = "less", conf.int = T)

wilcox.test(x = combined_nn$enapo_dist.12, y = combined_nn$en_dist.12, data = combined_nn, alternative = "less", conf.int = T)

# Apo distances from edu to nuclei are closer in apo anemones compared to edu-nuclei distances in sym anemones

```

# Fig 4E median comparisons

```{r, apo lists, echo = FALSE}

# Medians for edu-nuclei comparisons for apo and sym

median(combined_nn$enapo_dist.1, na.rm = T)

median(combined_nn$en_dist.1, na.rm = T)

median(combined_nn$enapo_dist.2, na.rm = T)

median(combined_nn$en_dist.2, na.rm = T)

median(combined_nn$enapo_dist.3, na.rm = T)

median(combined_nn$en_dist.3, na.rm = T)

median(combined_nn$enapo_dist.4, na.rm = T)

median(combined_nn$en_dist.4, na.rm = T)

median(combined_nn$enapo_dist.5, na.rm = T)

median(combined_nn$en_dist.5, na.rm = T)

median(combined_nn$enapo_dist.6, na.rm = T)

median(combined_nn$en_dist.6, na.rm = T)

median(combined_nn$enapo_dist.7, na.rm = T)

median(combined_nn$en_dist.7, na.rm = T)

median(combined_nn$enapo_dist.8, na.rm = T)

median(combined_nn$en_dist.8, na.rm = T)

median(combined_nn$enapo_dist.9, na.rm = T)

median(combined_nn$en_dist.9, na.rm = T)

median(combined_nn$enapo_dist.10, na.rm = T)

median(combined_nn$en_dist.10, na.rm = T)

median(combined_nn$enapo_dist.11, na.rm = T)

median(combined_nn$en_dist.11, na.rm = T)

median(combined_nn$enapo_dist.12, na.rm = T)

median(combined_nn$en_dist.12, na.rm = T)

```

# Fig 4E t-test comparisons

```{r, apo lists, echo = FALSE}

# t-tests describing the means of distributions (previously used Mann-Whitney to find differences between same distributions)

t.test(x = combined_nn$enapo_dist.1, y = combined_nn$en_dist.1, data = combined_nn)

t.test(x = combined_nn$enapo_dist.2, y = combined_nn$en_dist.2, data = combined_nn)

t.test(x = combined_nn$enapo_dist.3, y = combined_nn$en_dist.3, data = combined_nn)

t.test(x = combined_nn$enapo_dist.4, y = combined_nn$en_dist.4, data = combined_nn)

t.test(x = combined_nn$enapo_dist.5, y = combined_nn$en_dist.5, data = combined_nn)

t.test(x = combined_nn$enapo_dist.6, y = combined_nn$en_dist.6, data = combined_nn)

t.test(x = combined_nn$enapo_dist.7, y = combined_nn$en_dist.7, data = combined_nn)

t.test(x = combined_nn$enapo_dist.8, y = combined_nn$en_dist.8, data = combined_nn)

t.test(x = combined_nn$enapo_dist.9, y = combined_nn$en_dist.9, data = combined_nn)

t.test(x = combined_nn$enapo_dist.10, y = combined_nn$en_dist.10, data = combined_nn)

t.test(x = combined_nn$enapo_dist.11, y = combined_nn$en_dist.11, data = combined_nn)

t.test(x = combined_nn$enapo_dist.12, y = combined_nn$en_dist.12, data = combined_nn)

```

## plot: Figure 3A

```{r, apo lists, echo = FALSE}

#equal alpha

equal_alpha_plot <- ggplot() +

geom_density(aes(x=es_dist.1), alpha = 0.8, fill = "#d95f02", data=edusymfiles2) +

geom_density(aes(x=ns_dist.1), alpha = 0.8, fill = "#7570b3", linetype = 6, data=nucsymfiles2) +

geom_density(aes(x=es_dist.2), alpha = 0.8, fill = "#d95f02", data=edusymfiles2) +

geom_density(aes(x=ns_dist.2), alpha = 0.8, fill = "#7570b3", linetype = 6, data=nucsymfiles2) +

geom_density(aes(x=es_dist.4), alpha = 0.8, fill = "#d95f02", data=edusymfiles2) +

geom_density(aes(x=ns_dist.4), alpha = 0.8, fill = "#7570b3", linetype = 6, data=nucsymfiles2) +

geom_density(aes(x=es_dist.6), alpha = 0.8, fill = "#d95f02", data=edusymfiles2) +

geom_density(aes(x=ns_dist.6), alpha = 0.8, fill = "#7570b3", linetype = 6, data=nucsymfiles2) +

geom_density(aes(x=es_dist.8), alpha = 0.8, fill = "#d95f02", data=edusymfiles2) +

geom_density(aes(x=ns_dist.8), alpha = 0.8, fill = "#7570b3", linetype = 6, data=nucsymfiles2) +

geom_density(aes(x=es_dist.12), alpha = 0.8, fill = "#d95f02", data=edusymfiles2) +

geom_density(aes(x=ns_dist.12), alpha = 0.8, fill = "#7570b3", linetype = 6, data=nucsymfiles2) +

geom_vline(xintercept=7, linetype="dotted", size = .8) +

xlim(0,100) + theme_minimal() + xlab("nearest neighbor distance (microns)")

#ggsave("equalalpha.pdf", height = 5, width = 6.5)

equal_alpha_plot

#gradient alpha

gradient_plot <- ggplot() +

geom_density(aes(x=es_dist.1), alpha = 1, fill = "#d95f02", data=edusymfiles2) +

geom_density(aes(x=ns_dist.1), alpha = 1, fill = "#7570b3", linetype = 6, data=nucsymfiles2) +

geom_density(aes(x=es_dist.2), alpha = 0.9, fill = "#d95f02", data=edusymfiles2) +

geom_density(aes(x=ns_dist.2), alpha = 0.9, fill = "#7570b3", linetype = 6, data=nucsymfiles2) +

geom_density(aes(x=es_dist.4), alpha = 0.8, fill = "#d95f02", data=edusymfiles2) +

geom_density(aes(x=ns_dist.4), alpha = 0.8, fill = "#7570b3", linetype = 6, data=nucsymfiles2) +

geom_density(aes(x=es_dist.6), alpha = 0.7, fill = "#d95f02", data=edusymfiles2) +

geom_density(aes(x=ns_dist.6), alpha = 0.7, fill = "#7570b3", linetype = 6, data=nucsymfiles2) +

geom_density(aes(x=es_dist.8), alpha = 0.6, fill = "#d95f02", data=edusymfiles2) +

geom_density(aes(x=ns_dist.8), alpha = 0.6, fill = "#7570b3", linetype = 6, data=nucsymfiles2) +

geom_density(aes(x=es_dist.12), alpha = 0.5, fill = "#d95f02", data=edusymfiles2) +

geom_density(aes(x=ns_dist.12), alpha = 0.5, fill = "#7570b3", linetype = 6, data=nucsymfiles2) +

geom_vline(xintercept=7, linetype="dotted", size = .8) +

xlim(0,100) + theme_minimal() + xlab("nearest neighbor distance (microns)")

#ggsave("gradientalpha.pdf", height = 5, width = 6.5)

gradient_plot

```

## figure 3 sym-apo t-test compoarison

```{r, apo lists, echo = FALSE}

apo_sym_mean <- combined_nn %>% summarise_all(funs(mean(., na.rm = TRUE)))

apo_sym_sd <- combined_nn %>% summarise_all(funs(sd(., na.rm = TRUE)))

apo_sym_median <- combined_nn %>% summarise_all(funs(median(., na.rm = TRUE)))

apo_sym_IQR <- combined_nn %>% summarise_all(funs(IQR(., na.rm = TRUE)))

apo_sym_mad <- combined_nn %>% summarise_all(funs(mad(., na.rm = TRUE)))

apo_sym_N <- combined_nn %>% summarise_all(funs(n_distinct(., na.rm = TRUE)))

apo_sym <- bind_rows(apo_sym_mean, apo_sym_sd, apo_sym_median, apo_sym_IQR, apo_sym_mad, apo_sym_N)

apo_sym_sum <- transpose(apo_sym)

colnames(apo_sym_sum) <- c("mean","sd", "median", "IQR", "mad", "n")

rownames(apo_sym_sum) <- colnames(apo_sym)

NNname <- rownames(apo_sym_sum)

apo_sym_sum <- cbind(apo_sym_sum,NNname)

apo_sym_sum_sub <- apo_sym_sum[rownames(apo_sym_sum) %like% "dist.", ]

apo_sym_final <- separate(apo_sym_sum_sub, NNname, c("name","dist","NN"))

apo_sym_final$NN <- as.numeric(apo_sym_final$NN)

apo_sym_final$NN <- as.factor(apo_sym_final$NN)

#apo_sym_final_reordered <- apo_sym_final %>% arrange(NN,name)

```

## plot figure 3B,3C t-test

```{r, apo lists, echo = FALSE}

sym_only <- apo_sym_final[rownames(apo_sym_final) %like% "es|ns" ,]

sym_en_mean_plot <- ggplot(sym_only, aes(x = NN, y=mean, shape = name, color = name, group = name)) +

geom_errorbar(aes(ymin=mean-sd, ymax=mean+sd), color = "dark gray", width = 0.4, position=position_dodge(width=.5)) +

geom_point(position=position_dodge(width=.5)) +

geom_signif(y_position = c(46,52,59,65,70,76,81,86,90,94,97,100),

xmin = c(0.75,1.75,2.75,3.75,4.75,5.75,6.75,7.75,8.75,9.75,10.75,11.75),

xmax = c(1.25,2.25,3.25,4.25,5.25,6.25,7.25,8.25,9.25,10.25,11.25,12.25),

annotation = c("*"), tip_length = 0,color = "black") +

theme_minimal() + xlab(expression(paste("Nearest Neighbor (", italic("k"), " 1-12 closest objects)"))) +

ylab("mean NN distance (microns)") +

scale_y_continuous(limits = c(-5, 100)) +

#scale_fill_discrete(name = "State", labels = c("Apo","Sym")) +

theme(legend.position = c(0.89, 0.12)) +

labs(color = "State", shape = "State") +

scale_color_manual(labels = c("EdU+", "Hoechst"), values = c("#d95f02", "#7570b3")) +

scale_shape_manual(labels = c("EdU+", "Hoechst"), values = c(17, 19))

sym_en_mean_plot

#ggsave("sym_en_mean_plot.eps", height = 6, width = 9.5)

sym_en_median_plot <- ggplot(sym_only, aes(x = NN, y=median, shape = name, color = name, group = name)) +

geom_errorbar(aes(ymin=median-IQR, ymax=median+IQR), color = "dark gray", width = 0.4, position=position_dodge(width=.5)) +

geom_point(position=position_dodge(width=.5)) +

geom_signif(y_position = c(29,35,43,48,53,57,61,64,68,71,75,79),

xmin = c(0.75,1.75,2.75,3.75,4.75,5.75,6.75,7.75,8.75,9.75,10.75,11.75),

xmax = c(1.25,2.25,3.25,4.25,5.25,6.25,7.25,8.25,9.25,10.25,11.25,12.25),

annotation = c("*"), tip_length = 0,color = "black") +

theme_minimal() + xlab(expression(paste("Nearest Neighbor (", italic("k"), " 1-12 closest objects)"))) +

ylab("median NN distance (microns)") +

scale_y_continuous(limits = c(0, 100)) +

#scale_fill_discrete(name = "State", labels = c("Apo","Sym")) +

theme(legend.position = c(0.89, 0.12)) +

labs(color = "State", shape = "State") +

scale_color_manual(labels = c("EdU+", "Hoechst"), values = c("#d95f02", "#7570b3")) +

scale_shape_manual(labels = c("EdU+", "Hoechst"), values = c(17, 19))

sym_en_median_plot

#ggsave("sym_en_median_plot.pdf", height = 6, width = 9.5)

#ggsave("sym_en_median_plot.eps", height = 6, width = 9.5)

```

## plot: Figure 4D

```{r, apo lists, echo = FALSE}

# EDU

edu_nuc_nn_plot <- ggplot() +

geom_density(aes(x=enapo_dist.1), alpha = 0.8, fill = "pink", data=eduapofiles2) +

geom_density(aes(x=en_dist.1), alpha = 0.8, fill = "#1b9e77", data=edunucsfiles2) +

geom_density(aes(x=enapo_dist.2), alpha = 0.8, fill = "pink", data=eduapofiles2) +

geom_density(aes(x=en_dist.2), alpha = 0.8, fill = "#1b9e77", data=edunucsfiles2) +

geom_density(aes(x=enapo_dist.4), alpha = 0.8, fill = "pink", data=eduapofiles2) +

geom_density(aes(x=en_dist.4), alpha = 0.8, fill = "#1b9e77", data=edunucsfiles2) +

geom_density(aes(x=enapo_dist.6), alpha = 0.8, fill = "pink", data=eduapofiles2) +

geom_density(aes(x=en_dist.6), alpha = 0.8, fill = "#1b9e77", data=edunucsfiles2) +

geom_density(aes(x=enapo_dist.8), alpha = 0.8, fill = "pink", data=eduapofiles2) +

geom_density(aes(x=en_dist.8), alpha = 0.8, fill = "#1b9e77", data=edunucsfiles2) +

geom_density(aes(x=enapo_dist.12), alpha = 0.8, fill = "pink", data=eduapofiles2) +

geom_density(aes(x=en_dist.12), alpha = 0.8, fill = "#1b9e77", data=edunucsfiles2) +

xlim(0,35) + theme_minimal() + xlab("Nearest Neighbor distance (microns)")

edu_nuc_nn_plot

#ggsave("edu-nuc-nn.pdf", height = 4, width = 7.5)

```

## plot figure 4E

```{r, apo lists, echo = FALSE}

apo_sym_only <- apo_sym_final[rownames(apo_sym_final) %like% "en", ]

apo_sym_only$name <- as.factor(apo_sym_only$name)

apo_sym_only$name <- factor(apo_sym_only$name,levels=c("enapo","en"))

apo_sym_en_median_plot <- ggplot(apo_sym_only, aes(x = NN, y=median, shape = name, color = name, group = name)) +

geom_errorbar(aes(ymin=median-mad, ymax=median+mad), color = "dark gray", width = 0.4, position=position_dodge(width=.5)) +

geom_point(position=position_dodge(width=.5)) +

geom_signif(y_position = c(9,11,13,14.5,16,17,18,19,20,21,22,23),

xmin = c(0.75,1.75,2.75,3.75,4.75,5.75,6.75,7.75,8.75,9.75,10.75,11.75),

xmax = c(1.25,2.25,3.25,4.25,5.25,6.25,7.25,8.25,9.25,10.25,11.25,12.25),

annotation = c("*"), tip_length = 0,color = "black") +

theme_minimal() + xlab(expression(paste("Nearest Neighbor (", italic("k"), " 1-12 closest objects)"))) +

ylab("median NN distance (microns)") +

scale_y_continuous(limits = c(0, 25)) +

#scale_fill_discrete(name = "State", labels = c("Apo","Sym")) +

theme(legend.position = c(0.85, 0.25)) +

labs(color = "State", shape = "State") +

scale_color_manual(labels = c("Apo", "Sym"), values = c("pink","#1b9e77")) +

scale_shape_manual(labels = c("Apo", "Sym"), values = c(19,17))

apo_sym_en_median_plot

#ggsave("apo_sym_en_median_plot_new.eps", height = 4, width = 8.5)

#ggsave("apo_sym_en_median_plot_new.pdf", height = 4, width = 8.5)

```

**Spatial_analyses_symbiont_location.rmd**

---

title: "Spatial_analyses_symbiont_location"

author: "Trevor Tivey"

date: "1/14/2020"

output: html_document

---

```{r setup, include=FALSE}

knitr::opts_chunk$set(echo = TRUE)

#setwd("/local/path/to/data")

set.seed(123)

#libraries

library(data.table)

library(ggplot2)

library(spatstat)

library(plyr)

library(dplyr)

library(tidyr)

library(ggsignif)

library(scales)

scientific_10 <- function(x) {

parse(text=gsub("e", " %*% 10^", scientific_format()(x)))

}

```

```{r, echo=FALSE}

### Create Apo Lists

setwd("./apo_analysis/apoout-h-r")

hrfiles <- list.files(pattern = ".txt")

hrfiles

apohrcombined <- adply(hrfiles, 1, read.csv, header = T, sep = "\t")

setwd("../apoout-v-r")

vrfiles <- list.files(pattern = ".txt")

vrfiles

apovrcombined <- adply(vrfiles, 1, read.csv, header = T, sep = "\t")

setwd("../apoout-e-r")

erfiles <- list.files(pattern = ".txt")

erfiles

apoercombined <- adply(erfiles, 1, read.csv, header = T, sep = "\t")

setwd("../..")

#write.csv(apohrcombined, "apo_combined_nuclei.csv")

#write.csv(apoercombined, "apo_combined_edu.csv")

#write.csv(apovrcombined, "apo_combined_volumes.csv")

### Create Sym Lists

setwd("./sym_analysis/nuclei")

hsfiles <- list.files(pattern = ".txt")

hsfiles

hscombined <- adply(hsfiles, 1, read.csv, header = T, sep = "\t")

setwd("../volume")

vsfiles <- list.files(pattern = ".txt")

vsfiles

vscombined <- adply(vsfiles, 1, read.csv, header = T, sep = "\t")

setwd("../edu")

esfiles <- list.files(pattern = ".txt")

esfiles

escombined <- adply(esfiles, 1, read.csv, header = T, sep = "\t")

setwd("../symbionts")

sfiles <- list.files(pattern = ".txt")

sfiles

scombined <- adply(sfiles, 1, read.csv, header = T, sep = "\t")

setwd("../..")

#write.csv(hscombined, "sym_combined_nuclei.csv")

#write.csv(escombined, "sym_combined_edu.csv")

#write.csv(scombined, "sym_combined_symbionts.csv")

#write.csv(vscombined, "sym_combined_volumes.csv")

```

### Summary Statistics

```{r, echo=FALSE}

# apo summary statistics

# hoechst apo

apohrsum <- ddply(apohrcombined, c("X1"), summarise,

hN = length(Volume..pixel.3.),

hmean = mean(Volume..pixel.3.),

hsd = sd(Volume..pixel.3.),

hse = hsd / sqrt(hN)

)

apohrsum

# edu apo

apoersum <- ddply(apoercombined, c("X1"), summarise,

eN = length(Volume..micron.3.),

emean = mean(Volume..micron.3.),

esd = sd(Volume..micron.3.),

ese = esd / sqrt(eN)

)

apoersum

# sym summary statistics

# hoechst symbiotic

symhssum <- ddply(hscombined, c("X1"), summarise,

hN = length(Volume..pixel.3.),

hmean = mean(Volume..pixel.3.),

hsd = sd(Volume..pixel.3.),

hse = hsd / sqrt(hN)

)

symhssum

# edu symbiotic

symessum <- ddply(escombined, c("X1"), summarise,

eN = length(Volume..micron.3.),

emean = mean(Volume..micron.3.),

esd = sd(Volume..micron.3.),

ese = esd / sqrt(eN)

)

symessum

# symbiont symbiotic

symsymsum <- ddply(scombined, c("X1"), summarise,

sN = length(Volume..pixel.3.),

smean = mean(Volume..pixel.3.),

ssd = sd(Volume..pixel.3.),

sse = ssd / sqrt(sN)

)

symsymsum

```

### Combining Files

```{r, echo=FALSE}

symsymcom <- cbind(vscombined,symsymsum)

symcom <- cbind(vscombined,symessum)

symcom <- cbind(symcom,symhssum)

apocom <- cbind(apovrcombined,apoersum)

apocom <- cbind(apocom,apohrsum)

len_sym <- length(symcom$eN) #95

symcom$state <- rep("sym", len_sym)

len_apo <- length(apocom$eN)

apocom$state <- rep("apo", len_apo)

symapocom <- rbind(symcom,apocom)

```

### Calculate densities

```{r, echo=FALSE}

# Calculate densities

symsymcom$symsymdensity = symsymcom$sN/symsymcom$Volume..micron.3.

symsymcom$symsymdensitymm3 = symsymcom$symsymdensity * 1000000000

mean(symsymcom$symsymdensity)

mean(symsymcom$symsymdensitymm3) # mm3 5.0e5/mm3

sd(symsymcom$symsymdensitymm3) # mm3 2.3e5/mm3

mean(symsymcom$symsymdensitymm3^(2/3)) # mm2 6.4e3/mm2

sd(symsymcom$symsymdensitymm3^(2/3)) # mm2 1.8e3/mm2

range(symsymcom$symsymdensitymm3^(2/3)) # mm2 3.2e3/mm2 to 1.2e4/mm2

182401^(2/3)

1297735^(2/3)

symapocom$edudensity = symapocom$eN/symapocom$Volume..micron.3.

symapocom$edudensitymm3 = symapocom$edudensity * 1000000000

symapocom$edudensity1000 = symapocom$edudensity * 1000000000

symapocom$edudensity2 = symapocom$eN/symapocom$Surface..micron.2.

symapocom$edudensity2mm2 = symapocom$edudensity2 * 1000000

symapocom$hdensity = symapocom$hN/symapocom$Volume..micron.3.

symapocom$hdensitymm3 = symapocom$hdensity * 1000000000

symapocom$percentedu = symapocom$eN/symapocom$hN * 100

symapocom$cellsize = symapocom$Volume..micron.3./symapocom$hN

radiusofhostcell <- (3/(4*pi) * symapocom$cellsize)^(1/3)

symapocom$cellsizediameter = 2*(radiusofhostcell)

```

### T-tests comparing EdU Density, Percent EdU, Hoecsht Density by symbiotic state.

```{r, echo=FALSE}

t.test(edudensity ~ state, data = symapocom) #0.0232

t.test(edudensitymm3 ~ state, data = symapocom) #3.5e5 v 3.9e5

t.test(percentedu ~ state, data = symapocom) #8.747e-5 8.6 v 10.2

t.test(hdensity ~ state, data = symapocom) #0.002189 0.0041 v 0.0038

t.test(hdensitymm3 ~ state, data = symapocom) #4.1e6 v 3.8e6

t.test(cellsizediameter ~ state, data = symapocom) #0.002113 7.80 v 7.95

wilcox.test(edudensity ~ state, data = symapocom) #0.0256

wilcox.test(percentedu ~ state, data = symapocom) #0.0005

wilcox.test(hdensity ~ state, data = symapocom) #0.0015

qqnorm(symapocom$edudensitymm3)

qqline(symapocom$edudensitymm3)

qqnorm(symapocom$hdensitymm3)

qqline(symapocom$hdensitymm3)

qqnorm(symapocom$percentedu)

qqline(symapocom$percentedu)

#apos have higher nuclei density but lower edu density

#apos have lower percent density of edu as well

```

### Summarizing statistics for plotting data

```{r, echo=FALSE}

symapocom_clean <- symapocom[, !duplicated(colnames(symapocom))]

symapocom_ed <- ddply(symapocom_clean, c("state"), summarise,

N = length(edudensitymm3),

mean = mean(edudensitymm3),

sd = sd(edudensitymm3),

se = sd / sqrt(N),

median = median(edudensitymm3),

mad = mad(edudensitymm3)

)

symapocom_ed

symapocom_ep <- ddply(symapocom_clean, c("state"), summarise,

N = length(percentedu),

mean = mean(percentedu),

sd = sd(percentedu),

se = sd / sqrt(N),

median = median(percentedu),

mad = mad(percentedu))

symapocom_ed

symapocom_hd <- ddply(symapocom_clean, c("state"), summarise,

N = length(hdensitymm3),

mean = mean(hdensitymm3),

sd = sd(hdensitymm3),

se = sd / sqrt(N),

median = median(hdensitymm3),

mad = mad(hdensitymm3))

symapocom_hd

```

### Plots comparing (1) EdU density (2) EdU percentage and (3) Nuclei Density

```{r, echo=FALSE}

symapocom$state <- factor(symapocom$state, levels=c("apo","sym"),

labels=c("Apo","Sym"))

ggplot(data = symapocom, aes(x = state, y = edudensitymm3, fill = state)) +

geom_boxplot(aes(fill = state)) +

scale_y_continuous(label= function(x) {

ifelse(x==0, "0", parse(text=gsub("[+]", "", gsub("e", " %*% 10^", scientific_format()(x)))))

},limits = c(0, 1000000),breaks = seq(0,1000000,100000)) +

theme_minimal() +

theme(axis.text.x = element_text(angle=0, size=10 ), legend.position="none") +

scale_fill_manual(labels=c("Apo","Sym"),values=c("pink", "#1b9e77")) +

labs(x="State",y = expression(Proliferative ~ nuclei ~ density ~ ~ (nuclei ~ mm^{-3})))

#ggsave("edudensity_fig3.pdf", height = 5, width = 2)

ggplot(data = symapocom, aes(x = state, y = percentedu, fill = state)) +

geom_boxplot(aes(group = state, fill = state)) +

scale_y_continuous(limits = c(0, 25),breaks = seq(0,25,5)) +

theme_minimal() +

theme(axis.text.x = element_text(angle=0, size=10 ), legend.position="none") +

scale_fill_manual(labels=c("Apo","Sym"),values=c("pink", "#1b9e77")) +

labs(x="State",y = expression("% " ~ Proliferative ~ nuclei))

#ggsave("edupercent_fig3b.pdf", height = 5, width = 2)

ggplot(data = symapocom, aes(x = state, y = hdensitymm3)) +

geom_boxplot(aes(group = state, fill = state)) +

scale_y_continuous(label= function(x) {

ifelse(x==0, "0", parse(text=gsub("[+]", "", gsub("e", " %*% 10^", scientific_format()(x)))))

}, limits = c(0, 8000000),breaks = seq(0,5e7,1e6)) +

theme_minimal() +

theme(axis.text.x = element_text(angle=0, size=10 ), legend.position="none") +

scale_fill_manual(labels=c("Apo","Sym"),values=c("pink", "#1b9e77")) +

labs(x="State",y = expression(Total ~ nuclei ~ density ~ ~ (nuclei ~ mm^{-3})))

#ggsave("hdensity_fig3c.pdf", height = 5, width = 2)

```

### Find where nuclei are located on z-axis to determine whether gastrodermal or epidermal

```{r, echo=FALSE}

#escombined

#hscombined

#scombined

#apoercombined

#apohrcombined

#stat summary on Z for each tentacle imaged.

#stat summary on

zhssum <- ddply(hscombined, c("X1"), summarise,

hN = length(Z),

hmean = mean(Z),

hsd = sd(Z),

hse = hsd / sqrt(hN),

hmedian = median(Z)

)

zssum <- ddply(scombined, c("X1"), summarise,

sN = length(Z),

smean = mean(Z),

ssd = sd(Z),

sse = ssd / sqrt(sN),

smedian = median(Z)

)

zessum <- ddply(escombined, c("X1"), summarise,

eN = length(Z),

emean = mean(Z),

esd = sd(Z),

ese = esd / sqrt(eN),

emedian = median(Z)

)

zahsum <- ddply(apohrcombined, c("X1"), summarise,

ahN = length(Z),

ahmean = mean(Z),

ahsd = sd(Z),

ahse = ahsd / sqrt(ahN),

ahmedian = median(Z)

)

zaesum <- ddply(apoercombined, c("X1"), summarise,

aeN = length(Z),

aemean = mean(Z),

aesd = sd(Z),

aese = aesd / sqrt(aeN),

aemedian = median(Z)

)

```

```{r, echo=FALSE}

# average z-axis location per tentacle

zaesum #apo edu

zahsum #apo hoechst

zessum #sym edu

zssum #sym sym

zhssum #sym hoechst

#test mean z of EdU vs hoechst

t.test(zaesum$aemean, zahsum$ahmean) # 0.1551 no difference, approx 9.5, 9.8

t.test(zessum$emean, zhssum$hmean) # 0.1642 no difference, approx 8.6m 8.3

t.test(zssum$smean, zhssum$hmean) # 0.0545 difference 8.8 8.3

#test difference between edu and hoechst paired per tentacle

t.test(zaesum$aemean, zahsum$ahmean,paired = T) # 2.2e-16 sig difference edu -.3

t.test(zessum$emean, zhssum$hmean, paired = T) # 4.9e-9 sig difference edu + 0.4

t.test(zssum$smean, zhssum$hmean, paired = T) # 3.4e-14 sig difference + 0.5

t.test(zssum$smean, zessum$emean, paired = T) # 0.037 sig difference + 0.1

#for each tentacle, EdU points are closer to mean symbionts than to hoechst

t.test(zaesum$aemedian, zahsum$ahmedian) # .196 9.3 9.6 enhanced in epidermis in apos

t.test(zaesum$aemedian, zahsum$ahmedian,paired = T) # 7.0e-5 sig difference edu -.3

t.test(zessum$emedian, zhssum$hmedian) # 0.249 7.8 7.5 EdU is found throughout in thicker gastrodermis

t.test(zessum$emedian, zhssum$hmedian, paired = T) # 0.001 sig difference edu + 0.3

t.test(zssum$smedian, zhssum$hmedian, paired = T) # 2.2e-16 sig difference + 1.25

t.test(zssum$smedian, zessum$emedian, paired = T) # 1.7e-10 sig difference + 0.94

#test differences between apo and sym distribution

apodistz = zahsum$ahmean - zaesum$aemean

symdistz = zhssum$hmean - zessum$emean

symdistedusymz = zssum$smean - zessum$emean

apodistmedz = zahsum$ahmedian - zaesum$aemedian

symdistmedz = zhssum$hmedian - zessum$emedian

symdistedusymmedz = zssum$smedian - zessum$emedian

symdistedusymmedz

symdistmedz

symnormalizeddf <- data.frame(symdistz,symdistedusymz, symdistmedz,symdistedusymmedz)

t.test(apodistz,symdistz) # 2.2e-16 different distributions, apo is

t.test(apodistmedz,symdistmedz) # 8.1e-7 different distributions, apo is

```

### Create summary dataframe for plotting distribution differences

```{r, echo=FALSE}

prolif_nuc_norm_apo_mean <- zaesum$aemean - zahsum$ahmean

prolif_nuc_norm_apo_med <- zaesum$aemedian - zahsum$ahmedian

prolif_nuc_norm_apo <- data.frame(prolif_nuc_norm_apo_mean, prolif_nuc_norm_apo_med)

prolif_nuc_norm_apo.tidy <- gather(prolif_nuc_norm_apo)

prolif_nuc_norm_sym <- zessum$emean - zhssum$hmean

prolif_nuc_norm_sym_mean <- zessum$emean - zhssum$hmean

prolif_nuc_norm_sym_med <- zessum$emedian - zhssum$hmedian

prolif_nuc_norm_sym <- data.frame(prolif_nuc_norm_sym_mean, prolif_nuc_norm_sym_med)

prolif_nuc_norm_sym.tidy <- gather(prolif_nuc_norm_sym)

combined_normalizeddf <- rbind(prolif_nuc_norm_apo.tidy, prolif_nuc_norm_sym.tidy)

combined_normalizeddf$key <- factor(combined_normalizeddf$key,

levels=c("prolif_nuc_norm_apo_med","prolif_nuc_norm_sym_med","prolif_nuc_norm_apo_mean","prolif_nuc_norm_sym_mean"))

combined_normalizeddf

combined_normalizeddf_med <- combined_normalizeddf[grep("med", (combined_normalizeddf$key)), ]

combined_normalizeddf_med$key <- factor(combined_normalizeddf_med$key,

levels=c("prolif_nuc_norm_apo_med","prolif_nuc_norm_sym_med"),

labels=c("Apo","Sym"))

```

```{r, echo=FALSE}

ggplot(data = combined_normalizeddf_med, aes(x = key, y = value)) +

geom_boxplot(aes(group = key, fill = key)) +

scale_y_continuous(limits = c(-3,4),breaks = seq(-3,4,0.5)) +

theme_minimal() +

theme(axis.text.x = element_text(angle=0, size=10 ), legend.position="none") +

scale_fill_manual(labels=c("Apo","Sym"), values=c("pink", "#1b9e77")) +

xlab("State") + ylab("Normalized Z-axis location") + labs("")

#ggsave("aposymzttest_fig3f.pdf", height = 5, width = 2)

#median

ggplot(symnormalizeddf, aes(x = symdistmedz, y = symdistedusymmedz)) +

geom_point() +

geom_density_2d() + theme_minimal() +

ylab("Distance from median symbiont center to EdU+ nuclei") +

xlab("Distance from median host nuclei to EdU+ nuclei") +

xlim(-4,4) + ylim(-4,4) +

geom_vline(xintercept=0, size = .8) +

geom_hline(yintercept=0, size = .8)

#mean

ggplot(symnormalizeddf, aes(x = symdistz, y = symdistedusymz)) +

geom_point() +

geom_density_2d() + theme_minimal() +

ylab("Distance from median symbiont center to EdU+ nuclei") +

xlab("Distance from median host nuclei to EdU+ nuclei") +

xlim(-4,4) + ylim(-4,4) +

geom_vline(xintercept=0, size = .8) +

geom_hline(yintercept=0, size = .8)

```

### Finding differences in distributions between apo, sym, hoechst, edu, symbiont combinations

```{r, echo=FALSE}

#escombined

#hscombined

#scombined

#apoercombined

#apohrcombined

t.test(vscombined$Volume..micron.3., apovrcombined$Volume..micron.3.)

t.test(apohrcombined$Z,apoercombined$Z) #apoh 10, apoe 9.6

t.test(escombined$Z,apoercombined$Z) #sym 9.2, apo 9.6

t.test(hscombined$Z,apohrcombined$Z) #sym 8.6, apo 9.9

t.test(escombined$Z,hscombined$Z) #sym 9.2, sym 8.6

t.test(escombined$Z,scombined$Z) #sym 9.2, sym 9.4

wilcox.test(vscombined$Volume..micron.3., apovrcombined$Volume..micron.3.)

wilcox.test(apohrcombined$Z,apoercombined$Z) #apoh 10, apoe 9.6

wilcox.test(escombined$Z,apoercombined$Z) #sym 9.2, apo 9.6

wilcox.test(hscombined$Z,apohrcombined$Z) #sym 8.6, apo 9.9

wilcox.test(escombined$Z,hscombined$Z) #sym 9.2, sym 8.6

wilcox.test(escombined$Z,scombined$Z) #sym 9.2, sym 9.4

wilcox.test(vscombined$Volume..micron.3., apovrcombined$Volume..micron.3.)

wilcox.test(apohrcombined$Z,apoercombined$Z) #apoh 10, apoe 9.6

wilcox.test(escombined$Z,apoercombined$Z) #sym 9.2, apo 9.6

wilcox.test(hscombined$Z,apohrcombined$Z) #sym 8.6, apo 9.9

wilcox.test(escombined$Z,hscombined$Z) #sym 9.2, sym 8.6

wilcox.test(escombined$Z,scombined$Z) #sym 9.2, sym 9.4

wilcox.test(x = combinedfiles2$es_dist.1, y = combinedfiles2$ns_dist.1, data = combinedfiles2, alternative = "less")

```

```{r, echo=FALSE}

ggplot() +

geom_density(aes(x=ahmedian), alpha = 0.5, fill = "blue", linetype = 2, data=zahsum) +

geom_density(aes(x=aemedian), alpha = 0.5, fill = "yellow", data=zaesum) +

xlim(0,20) + theme_minimal() + xlab("Z-axis location in tentacle (microns)")

#ggsave("line-ryb-apo-median.pdf", height = 5, width = 6.5)

ggplot() +

geom_density(aes(x=smedian), alpha = 0.5, fill = "red", linetype = 3, data=zssum) +

geom_density(aes(x=hmedian), alpha = 0.5, fill = "blue", linetype = 2, data=zhssum) +

geom_density(aes(x=emedian), alpha = 0.5, fill = "yellow", data=zessum) +

xlim(0,20) + theme_minimal() + xlab("Z-axis location in tentacle (microns)")

#ggsave("line-ryb-sym-median.pdf", height = 5, width = 6.5)

```

**Symbiont_bounding_box_analysis.rmd**

---

title: "Symbiont_bounding_box_analysis"

author: "Trevor Tivey"

date: "1/14/2020"

output: html_document

---

```{r setup, include=FALSE}

knitr::opts_chunk$set(echo = TRUE)

library(ggplot2)

library(data.table)

library(plyr)

library(scales)

```

### Step 1:

## Name file lists of symbiont bounding boxes (boxlist) and host total nuclei (pointlist)

```{r, echo=FALSE}

# Set working directory to folder with input files generated from FIJI 3D Object Counter. Files should be lists of symbiont and host objects.

# Symbiont object counter files (suffix = "_2.txt") and host total nuclei object counter files (suffix = " [1-3.txt]")

# Set working directory to folder containing x,y,z coordinates of cell populations

setwd("./sym_analysis/input sym")

# Create subset lists that are organized in corresponding order to each other

# Files containing object coordinates of all symbiont cluster centers of mass (autofluorescence)

boxlist = list.files(pattern = "C1-")

boxlist

# Files containing object coordinates of host EdU-labeled nuclei

edulist = list.files(pattern = " [1-3].txt")

edulist

# Files containing object coordinates of host nuclei (Hoechst labeled)

nuclist = list.files(pattern = "C4-")

nuclist

# Create output filenname list ahead of time (to be written over at the end)

filenm <- sub("txt", "csv", nuclist)

filenm

# pointlist and boxlist samples must correspond to each other. Check the list files to make sure samples are arranged correctly.

nuclist

boxlist

#######

####### Step 2:

####### For loop to determine if (x,y,z) EdU points are within (x,y,z) bounding boxes of symbionts

#######

# For each file read object counter text file as a tab separated csv file.

# points = all total host nuclei in one tentacle

# boxes = all symbiont objects in one tentacle

for(i in 1:length(nuclist)) {

setwd("./../input sym")

nucpoints <- read.csv(nuclist[i], header = T, sep = "\t")

boxes <- read.csv(boxlist[i], header = T, sep = "\t")

# define filenm within for loop

filenm <- sub("txt", "csv", nuclist)

# print to output

print(filenm[i])

# define X,Y,Z coordinates as numeric

nucpoints$X = as.numeric(as.character(nucpoints$X))

nucpoints$Y = as.numeric(as.character(nucpoints$Y))

nucpoints$Z = as.numeric(as.character(nucpoints$Z))

# define X,Y,Z coordinates of each symbiont bounding box corner as numeric

boxes$BX = as.numeric(as.character(boxes$BX))

boxes$BY = as.numeric(as.character(boxes$BY))

boxes$BZ = as.numeric(as.character(boxes$BZ))

boxes$B.width = as.numeric(as.character(boxes$B.width))

boxes$B.height = as.numeric(as.character(boxes$B.height))

boxes$B.depth = as.numeric(as.character(boxes$B.depth))

# define explicit X,Y,Z coordinates of box by adding width, height and depth to original X,Y,Z coordinate of each box.

boxes$BXmax <- boxes$BX + boxes$B.width

boxes$BYmax <- boxes$BY + boxes$B.height

boxes$BZmax <- boxes$BZ + boxes$B.depth

# turn dataframe into data table

nucpoints = as.data.table(nucpoints)

boxes = as.data.table(boxes)

# Create a boolean vector with length of "points": for each point in points, are X,Y,Z coordinates of points found between BX and BXmax, BY and BYmax, BZ and BZmax?

# It is necessary to do this in one step, if broken up there are many false positives.

boolw = apply(nucpoints, 1, function(x) {nrow(boxes[(as.numeric(x[11])>=BX) & (as.numeric(x[11])<=BXmax) & (as.numeric(x[12])>=BY) & (as.numeric(x[12])<=BYmax) & (as.numeric(x[13])>=BZ) & (as.numeric(x[13])<=BZmax),])>0})

nucpoints <- cbind(nucpoints, boolw)

boxes$Bxhalf <- 0.5 * (boxes$B.width / (2^(1/3)))

boxes$Byhalf <- 0.5 * (boxes$B.height / (2^(1/3)))

boxes$Bzhalf <- 0.5 * (boxes$B.depth / (2^(1/3)))

boxes$halfxmin <- boxes$X - boxes$Bxhalf

boxes$halfxmax <- boxes$X + boxes$Bxhalf

boxes$halfymin <- boxes$Y - boxes$Byhalf

boxes$halfymax <- boxes$Y + boxes$Byhalf

boxes$halfzmin <- boxes$Z - boxes$Bzhalf

boxes$halfzmax <- boxes$Z + boxes$Bzhalf

boolhalf = apply(nucpoints, 1, function(x) {nrow(boxes[(as.numeric(x[11])>=halfxmin) & (as.numeric(x[11])<=halfxmax) & (as.numeric(x[12])>=halfymin) & (as.numeric(x[12])<=halfymax) & (as.numeric(x[13])>=halfzmin) & (as.numeric(x[13])<=halfzmax),])>0})

nucpoints <- cbind(nucpoints, boolhalf)

# write new nuclei points file: now contains original nuclei points file with the last column as a boolean vector of whether or not the point is found within a symbiont cluster bounding box.

setwd("./../nuclei points")

write.csv(nucpoints, filenm[i])

}

```

```{r, echo=FALSE}

#edu point list

########################################################################

########################################################################

########################################################################

#######

####### Step 1:

####### Name file lists of symbiont bounding boxes (boxlist) and host total nuclei (pointlist)

#######

# Set working directory to folder with input files generated from FIJI 3D Object Counter. Files should be lists of symbiont and host objects.

# Symbiont object counter files (suffix = "_2.txt") and EdU+ nuclei object counter files (suffix = " [1-3.txt]")

setwd("./sym_analysis/input sym")

filenm <- sub("txt", "csv", edulist)

filenm

boxlist

edulist

# For loop to find if (x,y,z) EdU points are within (x,y,z) bounding boxes of symbionts

for(i in 1:length(edulist)) {

setwd("./../input sym")

points <- read.csv(edulist[i], header = T, sep = "\t")

boxes <- read.csv(boxlist[i], header = T, sep = "\t")

filenm <- sub("txt", "csv", edulist)

print(filenm[i])

points$X = as.numeric(as.character(points$X))

points$Y = as.numeric(as.character(points$Y))

points$Z = as.numeric(as.character(points$Z))

boxes$BX = as.numeric(boxes$BX)

boxes$BY = as.numeric(boxes$BY)

boxes$BZ = as.numeric(boxes$BZ)

boxes$B.width = as.numeric(boxes$B.width)

boxes$B.height = as.numeric(boxes$B.height)

boxes$B.depth = as.numeric(boxes$B.depth)

boxes$BXmax <- boxes$BX + boxes$B.width

boxes$BYmax <- boxes$BY + boxes$B.height

boxes$BZmax <- boxes$BZ + boxes$B.depth

points = as.data.table(points)

boxes = as.data.table(boxes)

boolw = apply(points, 1, function(x) {nrow(boxes[(as.numeric(x[12])>=BX) & (as.numeric(x[12])<=BXmax) & (as.numeric(x[13])>=BY) & (as.numeric(x[13])<=BYmax) & (as.numeric(x[14])>=BZ) & (as.numeric(x[14])<=BZmax),])>0})

points <- cbind(points, boolw)

boxes$Bxhalf <- 0.5 * (boxes$B.width / (2^(1/3)))

boxes$Byhalf <- 0.5 * (boxes$B.height / (2^(1/3)))

boxes$Bzhalf <- 0.5 * (boxes$B.depth / (2^(1/3)))

boxes$halfxmin <- boxes$X - boxes$Bxhalf

boxes$halfxmax <- boxes$X + boxes$Bxhalf

boxes$halfymin <- boxes$Y - boxes$Byhalf

boxes$halfymax <- boxes$Y + boxes$Byhalf

boxes$halfzmin <- boxes$Z - boxes$Bzhalf

boxes$halfzmax <- boxes$Z + boxes$Bzhalf

boolhalf = apply(points, 1, function(x) {nrow(boxes[(as.numeric(x[12])>=halfxmin) & (as.numeric(x[12])<=halfxmax) & (as.numeric(x[13])>=halfymin) & (as.numeric(x[13])<=halfymax) & (as.numeric(x[14])>=halfzmin) & (as.numeric(x[14])<=halfzmax),])>0})

points <- cbind(points, boolhalf)

setwd("./../edu points")

write.csv(points, filenm[i])

}

```

## INPUT and COMBINED FILES ##

```{r, echo=FALSE}

########################################################################

########################################################################

########################################################################

# Create subset lists that are organized in corresponding order to each other

# Files containing object coordinates of all symbiont cluster centers of mass (autofluorescence)

boxlist = list.files(pattern = "C1-")

boxlist

# Files containing object coordinates of host EdU-labeled nuclei

edulist = list.files(pattern = " [1-3].txt")

edulist

# Files containing object coordinates of host nuclei (Hoechst labeled)

nuclist = list.files(pattern = "C4-")

nuclist

# EdU combined csv file original (no test to see if inside or outside boxes)

setwd("./sym_analysis/input sym")

files <- list.files(pattern = " [1-3].txt")

files

combinededu <- adply(files, 1, read.csv, header = T, sep = "\t")

setwd("./../fig2")

write.csv(combinededu, "combined_edu.csv")

# EdU combined csv file with boolean test

setwd("./../edu points")

files <- list.files(pattern = ".csv")

files

combined <- adply(files, 1, read.csv, header = T)

setwd("./../fig2")

write.csv(combined, "combined.csv")

# Symbiont boxes combined csv file

setwd("./../input sym")

files <- list.files(pattern = "C1-")

files

combinedboxes <- adply(files, 1, read.csv, header = T, sep = "\t")

setwd("./../fig2")

write.csv(combinedboxes, "combined_boxes.csv")

# volumes for each tentacle image combined csv file

setwd("./../volume")

volumelist <- list.files(pattern = ".txt")

volumelist

combinedvolumes <- adply(volumelist, 1, read.csv, header = T, sep = "\t")

setwd("./../fig2")

write.csv(combinedvolumes, "combined_volumes.csv")

# nuclei for each tentacle image combined csv file

setwd("./../input sym")

files <- list.files(pattern = "C4-")

files

combinednuc <- adply(files, 1, read.csv, header = T, sep = "\t")

setwd("./../fig2")

write.csv(combinednuc, "combined_nuclei.csv")

# combined nuclei with booleans combined csv file

setwd("./../nuclei points")

files <- list.files(pattern = "csv")

files

combinednucb <- adply(files, 1, read.csv, header = T)

setwd("./../fig2")

write.csv(combinednucb, "combined_nuclei_bool.csv")

#read them all out again

combinedvolumes <- read.csv("combined_volumes.csv", header = T)

combinedboxes <- read.csv("combined_boxes.csv", header = T)

combined <- read.csv("combined.csv", header = T)

combinednuc <- read.csv("combined_nuclei.csv", header = T)

combinednucb <- read.csv("combined_nuclei_bool.csv", header = T, sep = ",")

```

```{r, echo=FALSE}

# estimated volume per tentacle of overlapping symbiont boxes, from another analysis.

setwd("./sym_analysis/fig2")

combined_redundant <- read.csv("combined_redundant.csv", header = T)

```

```{r, echo=FALSE}

# add column of X*Y*Z volume to symbiont cluster dataframe: combined volumes of symbionts in individual tentacles

combinedboxes$AABBvolume <- combinedboxes$B.width * combinedboxes$B.height * combinedboxes$B.depth

#indtents <- aggregate(Volume..micron.3. ~ X1, combinedboxes, sum)

# create summary data table of total symbiont volumes per tentacle

indtents <- aggregate(AABBvolume ~ X1, combinedboxes, sum)

# create summary data table of total symbiont volume per tentacle, defined directly by aggregated volume of symbiont objects (in pixels^3)

symvol <- aggregate(Volume..pixel.3. ~ X1, combinedboxes, sum)

# summary data table of total symbiont volume per tentacle (in voxels) #same as symvol

symvolvox <- aggregate(Nb.of.obj..voxels ~ X1, combinedboxes, sum)

# add aggregated symbiont AABB volumes to combined volumes dataframe

#combinedvolumesboxes <- cbind(combinedvolumes, indtents$Volume..pixel.3.)

combinedvolumesboxes <- cbind(combinedvolumes, indtents$AABBvolume)

colnames(combinedvolumesboxes)[29] <- "symbiontvolume"

combinedvolumesboxes <- cbind(combinedvolumesboxes, combined_redundant$x)

colnames(combinedvolumesboxes)[30] <- "symbiontcorrection"

combinedvolumesboxes$correctedsymbiontvolume <- combinedvolumesboxes$symbiontvolume - combinedvolumesboxes$symbiontcorrection

#add ratio

combinedvolumesboxes$symaporatio <- combinedvolumesboxes$correctedsymbiontvolume/combinedvolumesboxes$Volume..micron.3.

#add sample names

combinedvolumesboxes <- cbind(volumelist, combinedvolumesboxes)

combinedvolumesboxes <- cbind(combinedvolumesboxes, volumelist)

#subset samples

combinedvolumesboxes <- cbind(combinedvolumesboxes, symvol)

combinedvolumesboxes$symvolrat <- combinedvolumesboxes$Volume..pixel.3./combinedvolumesboxes$Volume..micron.3.

# subset in case symaporatio makes sense. in this case it doesn't need it.

tentaclesubset <- combinedvolumesboxes[combinedvolumesboxes$symaporatio >0,]

```

### Connecting boolean edu points to tentacles

```{r, echo=FALSE}

########

# aggregate total number of boolean overlaps per sample tentacle

indbools <- aggregate(boolw ~ X1, combined, length)

#select rows containing only true in combined boolean points

comselectedRows <- (combined$boolw == "TRUE")

comselectedRows

combinedreduced <- combined[comselectedRows,]

# number of overlaps (only trues) per tentacle sample

indboolstrue <- aggregate(boolw ~ X1, combinedreduced, length)

indboolstrue

# combined total and true-only lists

indbooltot <- cbind(indbools,indboolstrue$boolw)

indbooltot

#select subset of samples to match tentacle subset from before

selectedRowsind <- (indbooltot$X1 %in% tentaclesubset$X1)

selectedRowsind

indboolred <- indbooltot[selectedRowsind,]

indboolred

#### with half of the sym volume boolhalf

####

# aggregate total number of boolean overlaps per sample tentacle

halfindbools <- aggregate(boolhalf ~ X1, combined, length)

#select rows containing only true in combined boolean points

halfcomselectedRows <- (combined$boolhalf == "TRUE")

halfcomselectedRows

halfcombinedreduced <- combined[halfcomselectedRows,]

# number of overlaps (only trues) per tentacle sample

halfindboolstrue <- aggregate(boolhalf ~ X1, halfcombinedreduced, length)

halfindboolstrue

row24 <- c(24,0)

halfindboolstrueall <- rbind(halfindboolstrue[1:23,], row24, halfindboolstrue[24:94,])

# combined total and true-only lists

halfindbooltot <- cbind(halfindbools,halfindboolstrueall$boolhalf)

halfindbooltot

#select subset of samples to match tentacle subset from before

halfselectedRowsind <- (halfindbooltot$X1 %in% tentaclesubset$X1)

halfselectedRowsind

halfindboolred <- halfindbooltot[halfselectedRowsind,]

halfindboolred

#combined tentacle subset and boolean subsets

tentaclesubsetall <- cbind(tentaclesubset, indboolred[2],indboolred[3], halfindboolred[3])

# rename columns

colnames(tentaclesubsetall)[38] <- "totalpoints"

colnames(tentaclesubsetall)[39] <- "overlappoints"

colnames(tentaclesubsetall)[40] <- "halfoverlappoints"

# add columns for chi-square analysis

tentaclesubsetall$pointratio <- tentaclesubsetall$overlappoints/tentaclesubsetall$totalpoints

tentaclesubsetall$nonoverlappoints <- tentaclesubsetall$totalpoints - tentaclesubsetall$overlappoints

tentaclesubsetall$overlapexpected <- tentaclesubsetall$symvolrat * tentaclesubsetall$totalpoints # redo

tentaclesubsetall$pointspervolume = tentaclesubsetall$totalpoints/tentaclesubsetall$Volume..micron.3.

tentaclesubsetall$observed_expected <- tentaclesubsetall$overlappoints - tentaclesubsetall$overlapexpected # redo

tentaclesubsetall <- cbind(tentaclesubsetall, symvolvox$Nb.of.obj..voxels) #equals the same as symvolume, so instead of comparing to volume, compare to volume voxels

tentaclesubsetall$symvolrat2 <- tentaclesubsetall$Volume..pixel.3./tentaclesubsetall$Nb.of.obj..voxels

```

### Connect boolean nuclei points to tentacles

```{r, echo=FALSE}

#aggregate total number of boolean overlaps per sample tentacle

indboolsn <- aggregate(boolw ~ X1, combinednucb, length)

#select rows containing only false in combined boolean points

comselectedRowsn <- (combinednucb$boolw == "FALSE")

comselectedRowsn

combinedreducedn <- combinednucb[comselectedRowsn,]

# number of overlaps (only false) per tentacle sample

indboolsnfalse <- aggregate(boolw ~ X1, combinedreducedn, length)

indboolsnfalse

#combined total and true-only lists

indboolntot <- cbind(indboolsn,indboolsnfalse$boolw)

indboolntot

### half boolean

###

#aggregate total number of boolean overlaps per sample tentacle

halfindboolsn <- aggregate(boolhalf ~ X1, combinednucb, length)

#select rows containing only false in combined boolean points

halfcomselectedRowsn <- (combinednucb$boolhalf == "FALSE")

halfcomselectedRowsn

halfcombinedreducedn <- combinednucb[halfcomselectedRowsn,]

# number of overlaps (only false) per tentacle sample

halfindboolsnfalse <- aggregate(boolhalf ~ X1, halfcombinedreducedn, length)

halfindboolsnfalse

#combined total and true-only lists

halfindboolntot <- cbind(halfindboolsn,halfindboolsnfalse$boolhalf)

halfindboolntot

```

###combined tentacle subset and boolean subsets

```{r, echo=FALSE}

tentaclesubsetall <- cbind(tentaclesubsetall, indboolntot[2], indboolntot[3],halfindboolntot[3])

colnames(tentaclesubsetall)[48] <- "totalpointnuc"

colnames(tentaclesubsetall)[49] <- "nonoverlapnuc"

colnames(tentaclesubsetall)[50] <- "halfnonoverlapnuc"

```

### find volumes based on object voxels

```{r, echo=FALSE}

vol_n <- aggregate(Nb.of.obj..voxels ~ X1, combinednucb, sum)

vol_e <- aggregate(Nb.of.obj..voxels ~ X1, combined, sum)

vol_s <- aggregate(Nb.of.obj..voxels ~ X1, combinedboxes, sum)

vol_saabb <- aggregate(AABBvolume ~ X1, combinedboxes, sum)

vols_ens <- data.frame()

vols_ens <- cbind(volumelist,vol_n$Nb.of.obj..voxels,vol_e$Nb.of.obj..voxels,vol_s$Nb.of.obj..voxels, vol_saabb$AABBvolume, combinedvolumes$Nb.of.obj..voxels,combinedvolumes$Volume..micron.3.)

vols_ens <- data.frame(vols_ens)

names(vols_ens) <- c("sample","voln","vole","vols","volsaabb","vol","volmicron")

vols_ens$volsaabb <- as.numeric(as.character(vols_ens$volsaabb))

vols_ens$vols <- as.numeric(as.character(vols_ens$vols))

vols_ens$volsymred <- combined_redundant$x

vols_ens$scorrected <- vols_ens$volsaabb - vols_ens$volsymred

vols_ens$sratio <- vols_ens$volsaabb/vols_ens$vols

vols_ens$spercent <- vols_ens$vols/vols_ens$volsaabb *100

vols_ens$scorrectedratio <- vols_ens$scorrected/vols_ens$vols

vols_ens$scorrectedpercent <- vols_ens$vols/vols_ens$scorrected *100

```

### Connecting boolean nuclei points to tentacles

```{r, echo=FALSE}

#####

tentaclesubsetall$overlapnuc <- tentaclesubsetall$totalpointnuc - tentaclesubsetall$nonoverlapnuc

tentaclesubsetall$overlapnucexp <- tentaclesubsetall$symvolrat * tentaclesubsetall$totalpointnuc

tentaclesubsetall$nucpervolume = tentaclesubsetall$totalpointnuc/tentaclesubsetall$Volume..micron.3.

tentaclesubsetall$nuc_observed_expected <- tentaclesubsetall$overlapnuc - tentaclesubsetall$overlapnucexp

tentaclesubsetall$nonoverlapexpected <- tentaclesubsetall$totalpoints - tentaclesubsetall$overlapexpected

tentaclesubsetall$chisquare <- (tentaclesubsetall$overlappoints - tentaclesubsetall$overlapexpected)^2/tentaclesubsetall$overlapexpected +

(tentaclesubsetall$nonoverlappoints - tentaclesubsetall$nonoverlapexpected)^2/tentaclesubsetall$nonoverlapexpected

tentaclesubsetall$nonoverlapnucexpected <- tentaclesubsetall$totalpointnuc - tentaclesubsetall$overlapnucexp

tentaclesubsetall$chisquarenuc <- (tentaclesubsetall$overlapnuc - tentaclesubsetall$overlapnucexp)^2/tentaclesubsetall$overlapnucexp +

(tentaclesubsetall$nonoverlapnuc - tentaclesubsetall$nonoverlapnucexpected)^2/tentaclesubsetall$nonoverlapnucexpected

tentaclesubsetall$symvolrat3 <- tentaclesubsetall$correctedsymbiontvolume/tentaclesubsetall$Nb.of.obj..voxels

tentaclesubsetall$overlapexpected <- tentaclesubsetall$symvolrat3 * tentaclesubsetall$totalpoints

tentaclesubsetall$overlapnucexp <- tentaclesubsetall$symvolrat3 * tentaclesubsetall$totalpointnuc

tentaclesubsetall$nuc_observed_expected <- tentaclesubsetall$overlapnuc - tentaclesubsetall$overlapnucexp

tentaclesubsetall$observed_expected <- tentaclesubsetall$overlappoints - tentaclesubsetall$overlapexpected

tentaclesubsetall$nonoverlapexpected <- tentaclesubsetall$totalpoints - tentaclesubsetall$overlapexpected

tentaclesubsetall$chisquare <- (tentaclesubsetall$overlappoints - tentaclesubsetall$overlapexpected)^2/tentaclesubsetall$overlapexpected +

(tentaclesubsetall$nonoverlappoints - tentaclesubsetall$nonoverlapexpected)^2/tentaclesubsetall$nonoverlapexpected

tentaclesubsetall$nonoverlapnucexpected <- tentaclesubsetall$totalpointnuc - tentaclesubsetall$overlapnucexp

tentaclesubsetall$chisquarenuc <- (tentaclesubsetall$overlapnuc - tentaclesubsetall$overlapnucexp)^2/tentaclesubsetall$overlapnucexp +

(tentaclesubsetall$nonoverlapnuc - tentaclesubsetall$nonoverlapnucexpected)^2/tentaclesubsetall$nonoverlapnucexpected

tentaclesubsetfinal <- subset(tentaclesubsetall[tentaclesubsetall$symvolrat3 < 1,])

tentaclesubsetfinal$csbool <- "False"

tentaclesubsetfinal$csbool[tentaclesubsetfinal$chisquare > 3.84] <- "True"

tentaclesubsetfinal$csbooln <- "False"

tentaclesubsetfinal$csbooln[tentaclesubsetfinal$chisquarenuc > 3.84] <- "True"

```

```{r, echo=FALSE}

##extra half boolean

### observed overlap, observed nonoverlap, expected overlap, expected nonoverlap

# edu halfoverlappoints halfnonoverlapnuc

tentaclesubsetfinal$edu_nonoverlap_half <- tentaclesubsetfinal$totalpoints - tentaclesubsetfinal$halfoverlappoints

tentaclesubsetfinal$nuc_overlap_half <- tentaclesubsetfinal$totalpointnuc - tentaclesubsetfinal$halfnonoverlapnuc

#expected overlaps

tentaclesubsetfinal$halfsymvolrat3 <- tentaclesubsetfinal$symvolrat3 / 2

tentaclesubsetfinal$edu_overlap_exp <- tentaclesubsetfinal$totalpoints * tentaclesubsetfinal$halfsymvolrat3

tentaclesubsetfinal$edu_nonoverlap_exp <- tentaclesubsetfinal$totalpoints - tentaclesubsetfinal$edu_overlap_exp

tentaclesubsetfinal$nuc_overlap_exp <- tentaclesubsetfinal$totalpointnuc * tentaclesubsetfinal$halfsymvolrat3

tentaclesubsetfinal$nuc_nonoverlap_exp <- tentaclesubsetfinal$totalpointnuc - tentaclesubsetfinal$nuc_overlap_exp

tentaclesubsetfinal$halfchisquare <- (tentaclesubsetfinal$halfoverlappoints - tentaclesubsetfinal$edu_overlap_exp)^2/tentaclesubsetfinal$edu_overlap_exp +

(tentaclesubsetfinal$edu_nonoverlap_half - tentaclesubsetfinal$edu_nonoverlap_exp)^2/tentaclesubsetfinal$edu_nonoverlap_exp

tentaclesubsetfinal$halfchisquarenuc <- (tentaclesubsetfinal$nuc_overlap_half - tentaclesubsetfinal$nuc_overlap_exp)^2/tentaclesubsetfinal$nuc_overlap_exp +

(tentaclesubsetfinal$halfnonoverlapnuc - tentaclesubsetfinal$nuc_nonoverlap_exp)^2/tentaclesubsetfinal$nuc_nonoverlap_exp

tentaclesubsetall$halfchisquarenuc <- (tentaclesubsetall$overlapnuc - tentaclesubsetall$overlapnucexp)^2/tentaclesubsetall$overlapnucexp +

(tentaclesubsetall$nonoverlapnuc - tentaclesubsetall$nonoverlapnucexpected)^2/tentaclesubsetall$nonoverlapnucexpected

```

### graphs

```{r, echo=FALSE}

######## graphs

########

library(ggplot2)

# symbiont density on x-axis compared to observed - expected edu+ nuclei

ggplot(data=tentaclesubsetfinal, aes(x=symvolrat3,observed_expected)) + geom_point() + geom_smooth(method = 'glm') +

theme_classic() + labs(y = "# of EdU+ host nuclei within symbiont clusters \n (observed - expected)", x = "ratio of symbiont density \n(total volume of symbionts / total volume of tentacle)")

# host nuclei on x-axis compared to observed - expected edu+ nuclei

ggplot(data=tentaclesubsetfinal, aes(x=symvolrat3,nuc_observed_expected)) + geom_point() + geom_smooth(method = 'glm') +

theme_classic() + labs(y = "Host nuclei in symbiont clusters \n (observed - expected)", x = "ratio of symbiont density ratio of tentacle")

ggplot(data=tentaclesubsetfinal, aes(x=symvolrat3,observed_expected, color = csbool)) + geom_point() + geom_smooth(method = 'glm') +

theme_classic() + labs(y = "# of EdU+ host nuclei in symbiont clusters (observed - expected)", x = "symbiont density ratio of tentacle")

ggplot(data=tentaclesubsetfinal, aes(x=symvolrat3,nuc_observed_expected, color = csbooln)) + geom_point() + geom_smooth(method = 'glm') +

theme_classic() + labs(y = "# of host nuclei in symbiont clusters (observed - expected)", x = "symbiont density ratio of tentacle")

ggplot(data=tentaclesubsetfinal, aes(x=symvolrat3,chisquare)) + geom_point() + geom_smooth(method = 'glm') +

theme_classic() + labs(y = "Chi Squares of EdU+ host nuclei in symbiont clusters", x = "symbiont density ratio of tentacle")

ggplot(data=tentaclesubsetfinal, aes(x=chisquarenuc,chisquare)) + geom_point() + geom_smooth(method = 'glm') +

theme_classic() + labs(y = "Chi Squares of EdU+ host nuclei in symbiont clusters", x = "Chi Squares of host nuclei in symbiont clusters")

```

### Write final datasheets

```{r, echo=FALSE}

setwd("./sym_analysis/fig2")

write.csv(tentaclesubsetall, "tentaclesubsetall.csv")

write.csv(tentaclesubsetfinal, "tentaclesubsetfinal.csv")

tentaclesubsetfinal <- read.csv("tentaclesubsetfinal.csv", header = T)

```

### chi square info and summed chi square tests

```{r, echo=FALSE}

#####

########

#d.o.f = 1 so should be p = 0.05 of 3.84, p = 0.01 of 6.63, p = 0.001 10.8

# only 5 > 0.001 : 1.56, 9.15, 5.96, 0.38,

# tentacle 48 had 4 lowest numbers, along with tentacle 17 (which had only a couple symbionts). Tentacles from 17 were removed from analysis

# summed chi squared tests of nuclei overlapped with symbiont clusters

on <- sum(tentaclesubsetfinal$overlapnuc)

non <- sum(tentaclesubsetfinal$nonoverlapnuc)

one <- sum(tentaclesubsetfinal$overlapnucexp)

none <- sum(tentaclesubsetfinal$nonoverlapnucexpected)

ntcsqt <- (on - one)^2/one + (non - none)^2/none

ntcsqt

# 1273.212 redo = 10069.68

# summed chi squared tests of EdU+ nuclei overlapped with symbiont clusters

op <- sum(tentaclesubsetfinal$overlappoints)

nop <- sum(tentaclesubsetfinal$nonoverlappoints)

oe <- sum(tentaclesubsetfinal$overlapexpected)

noe <- sum(tentaclesubsetfinal$nonoverlapexpected)

pcsqt <- (op - oe)^2/oe + (nop - noe)^2/noe

pcsqt

# 609.975

# the numbers of EdU+ nuclei and total nuclei are significantly enriched in symbiont clusters

```

```{r, echo=FALSE}

######## more statistics

########

#paired t-test is used since numbers correlate to same samples.

t.test(x = tentaclesubsetfinal$overlappoints, y = tentaclesubsetfinal$overlapexpected, data = tentaclesubsetfinal, paired = T)

# p = 4.725 x 10^-12

t.test(x = tentaclesubsetfinal$overlapnuc, y = tentaclesubsetfinal$overlapnucexp, data = tentaclesubsetfinal, paired = T)

# p < 2.2 e-16 240 difference

#paired t-test is used since numbers correlate to same samples.

t.test(x = tentaclesubsetfinal$halfoverlappoints, y = tentaclesubsetfinal$edu_overlap_exp, data = tentaclesubsetfinal, paired = T)

# p = 0.0002 9 difference

t.test(x = tentaclesubsetfinal$nuc_overlap_half, y = tentaclesubsetfinal$nuc_overlap_exp, data = tentaclesubsetfinal, paired = T)

# p < 2.2 e-16 240 difference

#Symbiont density and observed - expected

symbiontdensityoelm <- lm(symvolrat3 ~ observed_expected, data = tentaclesubsetfinal)

summary(symbiontdensityoelm)

# pr = 3.44e-9, R = 0.3184

symbiontdensityedulm <- lm(symvolrat3 ~ totalpoints, data = tentaclesubsetfinal)

summary(symbiontdensityedulm)

# pr = 0.7988, R = -0.01049

# calculate edu+ density

tentaclesubsetfinal$edudensity <- tentaclesubsetfinal$totalpoints/tentaclesubsetfinal$Nb.of.obj..voxels

tentaclesubsetfinal$nucdensity <- tentaclesubsetfinal$totalpointnuc/tentaclesubsetfinal$Nb.of.obj..voxels

#symbiont volume ratio is associated with edudensity

symbiontdensityedudensitylm <- lm(symvolrat3 ~ edudensity, data = tentaclesubsetfinal)

summary(symbiontdensityedudensitylm)

# pr = 0.03683, R = 0.03736 F = 4.493

#symbiont volume ratio is not associated with nucdensity

symbiontdensitynucdensitylm <- lm(symvolrat3 ~ nucdensity, data = tentaclesubsetfinal)

summary(symbiontdensitynucdensitylm)

# pr = 0.3296, R = -0.0004342

```

# Figure 3E and F

```{r, echo=FALSE}

ggplot(data=tentaclesubsetfinal, aes(x=symvolrat3,edudensity)) + geom_point() + geom_smooth(method = 'glm') +

theme_classic() + labs(y = "EdU density", x = "ratio of symbiont density \n(total volume of symbionts / total volume of tentacle)")

setwd("./sym_analysis/fig2")

ggplot(data=tentaclesubsetfinal, aes(x=symvolrat3,totalpoints)) + geom_point() + geom_smooth(method = 'glm') +

theme_classic() + labs(y = "Total EdU points per tentacle", x = "ratio of symbiont density \n(total volume of symbionts / total volume of tentacle)")

ggplot(data=tentaclesubsetfinal, aes(x=symvolrat3,nucdensity)) + geom_point() + geom_smooth(method = 'glm') +

theme_classic() + labs(y = "Total host nuclei density", x = "ratio of symbiont density \n(total volume of symbionts / total volume of tentacle)")

# Figure 3F

ggplot(data=tentaclesubsetfinal, aes(x=symvolrat3,edudensity)) + geom_point() + geom_smooth(method = 'glm') +

theme_minimal() + labs(y = "EdU density", x = "Ratio of symbiont density \n(total volume of symbionts / total volume of tentacle)")

# Figure 3F

ggplot(data=tentaclesubsetfinal, aes(x=symvolrat3,nucdensity)) + geom_point() + geom_smooth(method = 'glm') +

theme_minimal() + labs(y = "Total host nuclei density", x = "Ratio of symbiont density \n(total volume of symbionts / total volume of tentacle)")

# Figure 3F no axes titles

ggplot(data=tentaclesubsetfinal, aes(x=symvolrat3,edudensity)) + geom_point() + geom_smooth(method = 'glm') +

theme_minimal() + labs(y = "", x = "") + scale_y_continuous(label = scientific_format(), limits = c(1e-5,1.6e-4), breaks = c(3e-5,6e-5,9e-5,1.2e-4,1.5e-4))

#ggsave("edu_density_x_ratio.pdf", height = 4, width = 4, useDingbats=FALSE)

# Figure 3F no axes titles

ggplot(data=tentaclesubsetfinal, aes(x=symvolrat3,nucdensity)) + geom_point() + geom_smooth(method = 'glm') +

theme_minimal() + labs(y = "", x = "") + scale_y_continuous(limits = c(4e-4, 9e-4))

#ggsave("hoechst_density_x_ratio.pdf", height = 4, width = 4, useDingbats=FALSE)

# Figure 3E

ggplot(data=tentaclesubsetfinal, aes(x=symvolrat3,overlappoints)) + geom_point() + geom_smooth(method = 'glm') +

theme_minimal() + labs(y = "", x = "")

#ggsave("overlap_x_ratio.pdf", height = 4, width = 4, useDingbats=FALSE)

# Figure 3E

ggplot(data=tentaclesubsetfinal, aes(x=symvolrat3,observed_expected)) + geom_point() + geom_smooth(method = 'glm') +

theme_minimal() + labs(y = "", x = "") + scale_y_continuous(limits = c(-40, 80), breaks = c(-40, 0, 40,80))

#ggsave("o-e_x_ratio.pdf", height = 4, width = 4, useDingbats=FALSE)

```

### Symbiont density comparisons using symbiont volume

```{r, echo=FALSE}

# symbiont density compared to number of proliferating cells within symbiont clusters

symbiontdensityolm <- lm(symvolrat3 ~ overlappoints, data = tentaclesubsetfinal)

summary(symbiontdensityolm)

# pr < 2e-16, R = 0.5986

# symbiont density compared to number of proliferating cells within symbiont clusters

symbiontdensityonuclm <- lm(symvolrat3 ~ nuc_observed_expected, data = tentaclesubsetfinal)

summary(symbiontdensityonuclm)

# pr < 2e-16, R = 0.5986

# symbiont density compared to number of proliferating cells outside of symbiont clusters

symbiontdensitynolm <- lm(symvolrat3 ~ nonoverlappoints, data = tentaclesubsetfinal)

summary(symbiontdensitynolm)

# pr < 7.95e-9, R = 0.3134

# compare symbiont density to density of edu or nuclei within symbiont clusters

# number of edu objects in clusters / total space of symbionts (correctedsymbiontvolume)

tentaclesubsetfinal$educlusterdensity <- tentaclesubsetfinal$overlappoints/tentaclesubsetfinal$correctedsymbiontvolume

tentaclesubsetfinal$nucclusterdensity <- tentaclesubsetfinal$overlapnuc/tentaclesubsetfinal$correctedsymbiontvolume

#

symbiontdensityeduclusterdensity <- lm(symvolrat3 ~ educlusterdensity, data = tentaclesubsetfinal)

summary(symbiontdensityeduclusterdensity)

# negative effect: pr = 0.88, R = -0.011

symbiontdensitynucclusterdensity <- lm(symvolrat3 ~ nucclusterdensity, data = tentaclesubsetfinal)

summary(symbiontdensitynucclusterdensity)

# negative effect: p = 0.38, R = -0.008

# compare symbiont density to density of edu or nuclei outside symbiont clusters

# number of edu objects outside clusters / total space of symbionts (correctedsymbiontvolume)

tentaclesubsetfinal$eduoutclusterdensity <- tentaclesubsetfinal$nonoverlappoints/(tentaclesubsetfinal$Nb.of.obj..voxels - tentaclesubsetfinal$correctedsymbiontvolume)

tentaclesubsetfinal$nucoutclusterdensity <- tentaclesubsetfinal$nonoverlapnuc/(tentaclesubsetfinal$Nb.of.obj..voxels - tentaclesubsetfinal$correctedsymbiontvolume)

# slight negative effects of symbiont density on density of nuclei and proliferating outside symbiont clusters

symbiontdensityeduoutclusterdensity <- lm(symvolrat3 ~ eduoutclusterdensity, data = tentaclesubsetfinal)

summary(symbiontdensityeduoutclusterdensity)

# negative effect: pr = 0.0381, R = 0.04743

symbiontdensitynucclusterdensity <- lm(symvolrat3 ~ nucoutclusterdensity, data = tentaclesubsetfinal)

summary(symbiontdensitynucclusterdensity)

# negative effect: pr = 4.11e-11, R = 0.3886

```

### Fig. 3D

```{r, echo=FALSE}

tentaclesubsetno24anova <- subset(tentaclesubsetfinal, select=c(overlappoints,nonoverlappoints,overlapexpected))

tentaclesubsetno24anova$nonoverlapexpected <- tentaclesubsetno24anova$overlappoints + tentaclesubsetno24anova$nonoverlappoints - tentaclesubsetno24anova$overlapexpected

ts24anovamelt <- melt(tentaclesubsetno24anova)

anovamelt <- lm(value ~ variable, ts24anovamelt)

summary(anovamelt)

melt.aov <- aov(value ~ variable, ts24anovamelt)

summary(melt.aov)

TukeyHSD(melt.aov)

t.test(x = tentaclesubsetfinal$overlappoints, y = tentaclesubsetfinal$overlapexpected, data = tentaclesubsetfinal)

# p = 0.0185 overlap expected and overlap points

qplot(data = ts24anovamelt, x = variable, y = value)

ts_sum <- ddply(ts24anovamelt, "variable", summarise,

tsmean = mean(value),

tsmedian = median(value),

tssd = sd(value),

tsIQR = IQR(value),

tsn = length(value)

)

ts_sum$variable <- as.factor(ts_sum$variable)

ts_sum$variable <- factor(ts_sum$variable,levels=c("overlapexpected","overlappoints","nonoverlapexpected","nonoverlappoints"))

qplot(data = ts_sum, x = variable, y = tsmean)

library(ggsignif)

ggplot(ts_sum, aes(x = variable, y=tsmean, shape = variable, color = variable, fill = variable, group = variable)) +

geom_errorbar(aes(ymin=tsmean-tssd, ymax=tsmean+tssd), color = "dark gray", width = 0.4, position=position_dodge(width=.5)) +

geom_point(position=position_dodge(width=.5), size = 3) +

scale_fill_manual(labels = c("overlappoints", "nonoverlappoints","overlapexpected", "nonoverlapexpected"), values = c("#FF555E", "#FF555E", "white", "white")) +

scale_color_manual(labels = c("overlappoints", "nonoverlappoints","overlapexpected", "nonoverlapexpected"), values = c("black", "black", "black", "black")) +

scale_shape_manual(labels = c("overlappoints", "nonoverlappoints","overlapexpected", "nonoverlapexpected"), values = c(22, 22, 22, 22)) +

theme_minimal() +

geom_signif(y_position = c(170,235),

xmin = c(0.8,2.8),

xmax = c(2.2,4.2),

annotation = c("*"), tip_length = 0,color = "black") +

xlab("") + ylab("") + theme(legend.position = "none")

setwd("./sym_analysis/fig2")

#ggsave("paired-t-tests-exp-obs.pdf", height = 4, width = 3, useDingbats=FALSE)

```

```{r, echo=FALSE}

#paired t-test is used since numbers correlate to same samples.

t.test(x = tentaclesubsetfinal$overlappoints, y = tentaclesubsetfinal$overlapexpected, data = tentaclesubsetfinal, paired = T)

# p = 4.725 x 10^-12

```
